# Supplementary material for: SnCl4 Promoted Efficient Cleavage of Acetal/Ketal Groups with the Assistance of Water in CH2Cl2
Source: Molecules. 2022 Nov 26;27(23):8258. doi: 10.3390/molecules27238258 (PMC9736348; doi:10.3390/molecules27238258)

Supporting Information

Table of Contents

1. **Figure S1**……………..………………………………….. ....**S2**
2. **Synthesis of substrates**……………………………….. ....**S2**
3. **References**...**S7**
4. **Copies of NMR spectra**...**S8**

1. Figure S1. Treatment of methyl 2,3-di-O-pivaloyl-*α*-D-glucopyranoside 8 with 1 equiv of $SnCl_4$ in dry *d*-CH_3Cl.

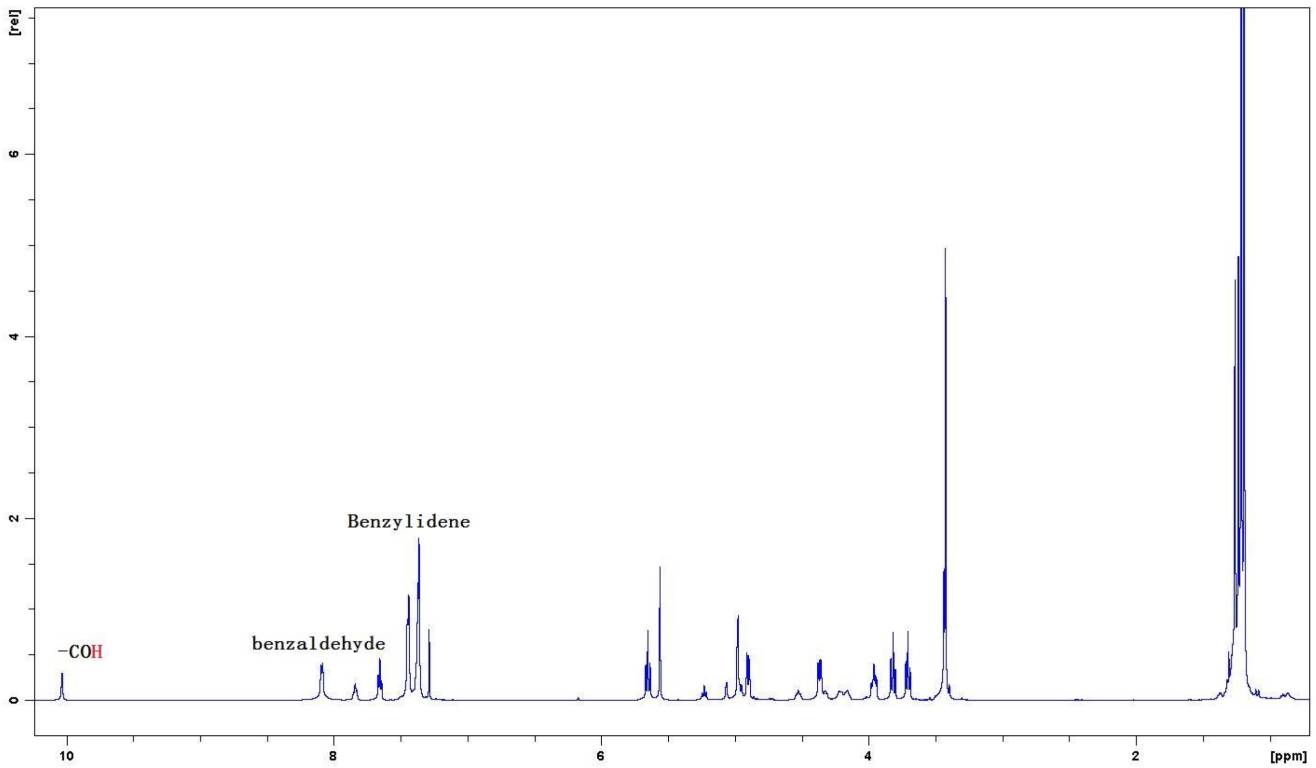

Benzaldehyde instead of dicholorotoluene was observed in the NMR spectrum. The reaction was terminated when the trace amounts of water in the *d*-choloroform is consumed.

2. Synthesis of substrates

Methyl 2,3-di-*O*-benzyl-4,6-*O*-benzylidene-*α*-D-glucopyranoside 1a

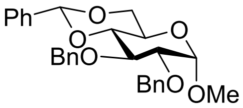

Methyl 2,3-di-*O*-benzyl-4,6-*O*-benzylidene-*α*-D-glucopyranoside **1a** was synthesized as previously reported.[1] ^{1}H NMR (600 MHz, $CDCl_3$) δ 7.56 – 7.43 (m, 2H), 7.43 – 7.22 (m, 13H), 5.55 (s, 1H), 4.91 (d, *J* = 11.3 Hz, 1H), 4.84 (dd, *J* = 11.7, 8.4 Hz, 2H), 4.70 (d, *J* = 12.1 Hz, 1H), 4.60 (d, *J* = 3.6 Hz, 1H), 4.26 (dd, *J* = 10.2, 4.8 Hz, 1H), 4.05 (t, *J* = 9.3 Hz, 1H), 3.83 (td, *J* = 10.0, 4.8 Hz, 1H), 3.71 (t, *J* = 10.3 Hz, 1H), 3.60 (t, *J* = 9.4 Hz, 1H), 3.56 (dd, *J* = 9.3, 3.7 Hz, 1H), 3.40 (s, 3H).

Methyl 2,3-di-*O*-benzyl-4,6-*O*-(*p*-methoxybenzylidene)-*α*-D-glucopyranoside 1b

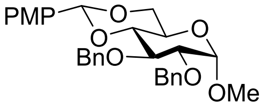

Methyl 2,3-di-*O*-benzyl-4,6-*O*-(*p*-methoxybenzylidene)-*α*-D-glucopyranoside **1b** was synthesized as previously reported.[1] ^{1}H NMR (400 MHz, $CDCl_3$) δ 7.50 – 7.15 (m, 12H), 6.94 – 6.81 (m, 2H), 5.51 (s, 1H), 4.97 – 4.78 (m, 3H), 4.69 (d, *J* = 12.2 Hz, 1H), 4.59 (d, *J* = 3.7 Hz, 1H), 4.24 (dd, *J* = 10.1, 4.7 Hz, 1H), 4.04 (t, *J* = 9.3 Hz, 1H), 3.82 (s, 4H), 3.69 (t, *J* = 10.2 Hz, 1H), 3.64 – 3.49 (m, 2H), 3.40 (s, 3H).

Methyl 2,3-di-*O*-benzyl-4,6-*O*-(*p*-bromobenzylidene)-*α*-D-glucopyranoside 1c

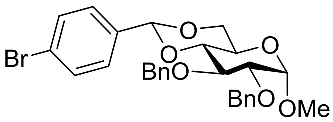

Methyl 2,3-di-*O*-benzyl-4,6-*O*-(*p*-bromobenzylidene)-*α*-D-glucopyranoside **1c** was synthesized as previously reported.[2] ^{1}H NMR (400 MHz, $CDCl_3$) δ 7.54 – 7.48 (m, 2H), 7.40 – 7.27 (m, 12H), 5.48 (s, 1H), 4.91 – 4.81 (m, 3H), 4.70 (d, *J* = 12.2 Hz, 1H), 4.59 (d, *J* = 3.7 Hz, 1H), 4.25 (dd, *J* = 10.1, 4.7 Hz, 1H), 4.02 (t, *J* = 9.3 Hz, 1H), 3.80 (td, *J* = 9.9, 4.7 Hz, 1H), 3.68 (t, *J* = 10.3 Hz, 1H), 3.61 – 3.53 (m, 2H), 3.40 (s, 3H).

Methyl 2,3-di-*O*-methyl-4,6-*O*-benzylidene-*α*-D-glucopyranoside 3

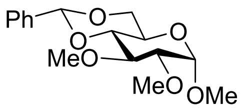

Methyl 2,3-di-*O*-methyl-4,6-*O*-benzylidene-*α*-D-glucopyranoside **3** was synthesized as previously reported.[3] Spectral data of **3**: ^{1}H NMR (400 MHz, $CDCl_3$) δ 7.61 – 7.44 (m, 2H), 7.44 – 7.31 (m, 3H), 5.55 (s, 1H), 4.86 (d, *J* = 3.7 Hz, 1H), 4.29 (dd, *J* = 9.9, 4.5 Hz, 1H), 3.82 (td, *J* = 9.7, 4.5 Hz, 1H), 3.71 (dt, *J* = 15.3, 9.6 Hz, 2H), 3.64 (s, 3H), 3.56 (s, 3H), 3.45 (s, 3H), 3.30 (dd, *J* = 9.2, 3.7 Hz, 1H).

Methyl 2,3-di-*O*-acetyl-4,6-*O*-benzylidene-*α*-D-glucopyranoside 5

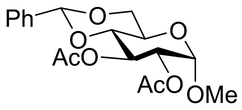

Methyl 2,3-di-*O*-acetyl-4,6-*O*-benzylidene-*α*-D-glucopyranoside **5** was synthesized as previously reported.[1] ^{1}H NMR (400 MHz, $CDCl_3$) δ 7.49 – 7.40 (m, 2H), 7.40 – 7.28 (m, 3H), 5.58 (t, *J* = 9.7 Hz, 1H), 5.50 (s, 1H), 5.01 – 4.86 (m, 2H), 4.30 (dd, *J* = 10.3, 4.8 Hz, 1H), 4.16 – 4.05 (m, 1H), 3.92 (td, *J* = 9.8, 4.6 Hz, 1H), 3.77 (t, *J* = 10.3 Hz, 1H), 3.65 (t, *J* = 9.6 Hz, 1H), 3.41 (s, 3H), 2.09 (s, 3H), 2.05 (s, 3H).

Methyl 2,3-di-*O*-pivaloyl-4,6-*O*-benzylidene-*α*-D-glucopyranoside 7

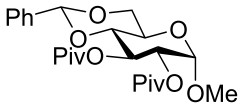

Methyl 2,3-di-*O*-pivaloyl-4,6-*O*-benzylidene-*α*-D-glucopyranoside **7** was synthesized as previously reported.[3,4] ^{1}H NMR (400 MHz, $CDCl_3$) δ 7.41 (dd, *J* = 6.8, 2.9 Hz, 2H), 7.37 – 7.30 (m, 3H), 5.61 (d, *J* = 9.8 Hz, 1H), 5.53 (s, 1H), 4.95 (d, *J* = 3.8 Hz, 1H), 4.86 (dd, *J* = 9.9, 3.7 Hz, 1H), 4.32 (dd, *J* = 10.2, 4.8 Hz, 1H), 3.93 (td, *J* = 9.9, 4.7 Hz, 1H), 3.78 (t, *J* = 10.3 Hz, 1H), 3.67 (t, *J* = 9.6 Hz, 1H), 3.40 (s, 3H), 1.19 (s, 9H), 1.16 (s, 9H).

Methyl 2,3-di-*O*-benzoyl-4,6-*O*-benzylidene-*β*-D-glucopyranoside 9

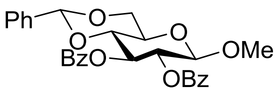

Methyl 2,3-di-*O*-benzoyl-4,6-*O*-benzylidene-*β*-D-glucopyranoside **9** was synthesized as previously reported.[1] ^{1}H NMR (400 MHz, $CDCl_3$) δ 7.96 (t, *J* = 7.6 Hz, 4H), 7.61 – 7.28 (m, 11H), 5.79 (t, *J* = 9.6 Hz, 1H), 5.56 (s, 1H), 5.47 (t, *J* = 8.7 Hz, 1H), 4.72 (d, *J* = 7.9 Hz, 1H), 4.46 (dd, *J* = 10.5, 4.9 Hz, 1H), 3.92 (dt, *J* = 14.3, 9.9 Hz, 2H), 3.71 (td, *J* = 9.7, 4.9 Hz, 1H), 3.54 (s, 3H).

Benzyl 2,3-di-*O*-acetyl-4,6-*O*-benzylidene-1-thio-*β*-D-glucopyranoside 11

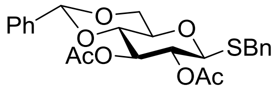

Benzyl 4,6-*O*-benzylidene-1-thio-*β*-D-glucopyranoside (100 mg, 0.27 mmol), pyridine (1.0 mL), and acetic anhydride (0.1 mL, 1.07 mmol) were combined in a round-bottom flask and stirred at room temperature for 2 h. After completion of reaction, the reaction mixture was concentrated under reduced pressure to give the crude compound. Purification on a silica gel column afforded **11** as a white solid (elution with ethyl acetate/petroleum ether =1:4 (v/v); 110 mg, 90% yield). m.p. 214.8-217.4, $[\alpha]_D^{12}$ = -119.5 (c 0.4, $CHCl_3$). ^{1}H NMR (400 MHz, $CDCl_3$) δ 7.49 – 7.27 (m, 11H), 5.50 (s, 1H), 5.27 (t, *J* = 9.4 Hz, 1H), 5.08 (t, *J* = 9.5 Hz, 1H), 4.42 (d, *J* = 10.0 Hz, 1H), 4.35 (dd, *J* = 10.6, 4.9 Hz, 1H), 3.89 (q, *J* = 12.9 Hz, 2H), 3.77 (t, *J* = 10.2 Hz, 1H), 3.68 (t, *J* = 9.6 Hz, 1H), 3.47 (td, *J* = 9.7, 4.9 Hz, 1H), 2.04 (s, 3H), 2.03 (s, 3H). ^{13}C NMR (100 MHz, $CDCl_3$) δ 170.04, 169.67, 136.86, 136.73, 129.16, 129.03, 128.65, 128.26, 127.45, 126.12, 101.49, 83.07, 78.36, 72.71, 70.64, 70.52, 68.45, 34.25, 20.79, 20.72. HRMS (ESI-TOF) m/z $[M + Na]^+$ calcd for $[C_{24}H_{26}O_7SNa]^+$: 481.1297; found: 481.1295.

4-Methylphenyl 2,3-di-*O*-acetyl-4,6-*O*-benzylidene-1-thio-*β*-D-glucopyranoside 13

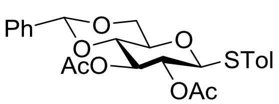

4-Methylphenyl 2,3-di-*O*-acetyl-4,6-*O*-benzylidene-1-thio-D-glucopyranoside **13** was synthesized as previously reported.[5] ^{1}H NMR (400 MHz, $CDCl_3$) δ 7.51 – 7.31 (m, 7H), 7.15 (d, *J* = 7.8 Hz, 2H), 5.49 (s, 1H), 5.33 (t, *J* = 9.4 Hz, 1H), 4.97 (t, *J* = 9.5 Hz, 1H), 4.74 (d, *J* = 10.1 Hz, 1H),

4.38 (dd, J = 10.7, 4.8 Hz, 1H), 3.79 (t, J = 10.2 Hz, 1H), 3.71 – 3.49 (m, 1H), 2.36 (s, 3H), 2.10 (d, J = 5.8 Hz, 3H), 2.03 (s, 3H).

Methyl 2,3-di-*O*-benzyl-4,6-*O*-benzylidene-*α*-D-galactopyranoside 15

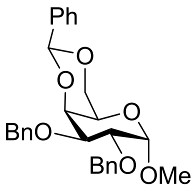

Methyl 2,3-di-*O*-benzyl-4,6-*O*-benzylidene-*α*-D-galactopyranoside **15** was synthesized as previously reported.[1] ^{1}H NMR (400 MHz, $CDCl_3$) δ 7.60 – 7.47 (m, 2H), 7.35 (tdd, J = 27.0, 13.9, 7.4 Hz, 13H), 5.48 (s, 1H), 4.86 (dd, J = 19.5, 12.2 Hz, 2H), 4.79 – 4.62 (m, 3H), 4.25 – 4.14 (m, 2H), 4.07 (dd, J = 10.1, 3.4 Hz, 1H), 3.99 (td, J = 10.1, 2.6 Hz, 2H), 3.58 (s, 1H), 3.38 (s, 3H).

Methyl 2,3-di-*O*-benzyl-4,6-*O*-benzylidene-*β*-D-galactopyranoside 17

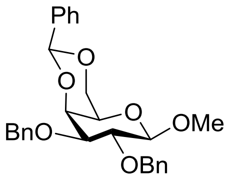

Methyl 2,3-di-*O*-benzyl-4,6-*O*-benzylidene-*β*-D-galactopyranoside **17** was synthesized as previously reported.[6] ^{1}H NMR (400 MHz, $CDCl_3$) δ 7.60 – 7.52 (m, 2H), 7.34 (dd, J = 22.1, 16.2 Hz, 13H), 5.50 (s, 1H), 4.91 (d, J = 10.9 Hz, 1H), 4.84 – 4.70 (m, 3H), 4.37 – 4.26 (m, 2H), 4.11 (dd, J = 3.7, 1.0 Hz, 1H), 4.02 (dd, J = 12.3, 1.8 Hz, 1H), 3.84 (dd, J = 9.7, 7.7 Hz, 1H), 3.59 (s, 3H), 3.56 (dd, J = 9.7, 3.6 Hz, 1H).

Methyl 2,3-di-*O*-acetyl-4,6-*O*-benzylidene-*α*-D-mannopyranoside 19

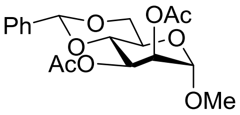

Methyl 2,3-di-*O*-acetyl-4,6-*O*-benzylidene-*α*-D-mannopyranoside **19** was synthesized according to the procedure for the synthesis of **5**.[7] ^{1}H NMR (400 MHz, $CDCl_3$) δ 7.46 (dd, J = 6.8, 2.8 Hz, 2H), 7.36 (dd, J = 5.2, 2.1 Hz, 3H), 5.58 (s, 1H), 5.45 – 5.32 (m, 2H), 4.67 (d, J = 1.6 Hz, 1H), 4.29 (dd, J = 10.0, 4.4 Hz, 1H), 4.05 (t, J = 9.7 Hz, 1H), 3.95 (td, J = 9.6, 4.4 Hz, 1H), 3.86 (t, J = 10.1 Hz, 1H), 3.40 (s, 3H), 2.17 (s, 3H), 2.01 (s, 3H).

Methyl 2,6-di-*O*-benzyl 3,4-*O*-isopropylidene-*α*-D-galactopyranoside 21

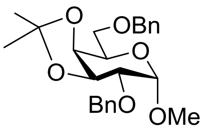

Methyl 2,6-di-*O*-benzyl 3,4-*O*-isopropylidene-*α*-D-galgactopyranoside **21** was synthesized as previously reported.[8] ^{1}H NMR (400 MHz, $CDCl_3$) δ 7.42 – 7.23 (m, 10H), 4.82 (d, J = 12.6 Hz, 1H), 4.76 – 4.61 (m, 3H), 4.53 (d, J = 12.1 Hz, 1H), 4.34 (dd, J = 7.9, 5.4 Hz, 1H), 4.25 – 4.07 (m, 2H), 3.82 – 3.64 (m, 2H), 3.51 (dd, J = 7.9, 3.5 Hz, 1H), 3.40 (s, 3H), 1.38 (s, 3H), 1.33 (s, 3H).

1,2:5,6-Di-*O*-isopropylidene-3-*O*-benzoyl-*α*-D-glucofuranose 23

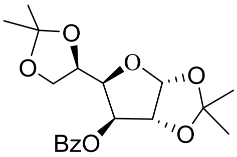

1,2:5,6-Di-*O*-isopropylidene-3-*O*-benzoyl-*α*-D-glucofuranose **23** was prepared using literature procedure.[9] ^{1}H NMR (400 MHz, $CDCl_3$) δ 8.09 – 7.95 (m, 2H), 7.67 – 7.54 (m, 1H), 7.46 (t, J = 7.6 Hz, 2H), 5.95 (d, J = 3.7 Hz, 1H), 5.51 (d, J = 2.7 Hz, 1H), 4.64 (d, J = 3.7 Hz, 1H), 4.35 (tt, J = 7.9, 4.0 Hz, 2H), 4.19 – 4.03 (m, 2H), 1.56 (s, 3H), 1.42 (s, 3H), 1.32 (s, 3H), 1.27 (s, 3H).

1,2:5,6-Di-*O*-isopropylidene-3-*O*-benzyl-*α*-D-glucofuranose 25

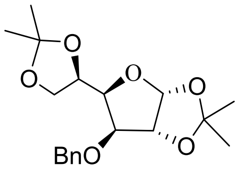

1,2:5,6-Di-*O*-isopropylidene-3-*O*-benzyl-*α*-D-glucofuranose **25** was synthesized as previously reported.[1] ^{1}H NMR (400 MHz, $CDCl_3$) δ 7.37 – 7.22 (m, 5H), 5.89 (d, *J* = 3.7 Hz, 1H), 4.73 – 4.60 (m, 2H), 4.58 (d, *J* = 3.7 Hz, 1H), 4.37 (dt, *J* = 7.7, 6.0 Hz, 1H), 4.15 (dd, *J* = 7.7, 3.1 Hz, 1H), 4.10 (dd, *J* = 8.6, 6.2 Hz, 1H), 4.07 – 3.96 (m, 2H), 1.48 (s, 3H), 1.42 (s, 3H), 1.37 (s, 3H), 1.30 (s, 3H).

Methyl 2,4,6-tri-*O*-acetyl-3-*O*-(*p*-methoxybenzyl)-*β*-D-galactopyranoside 27

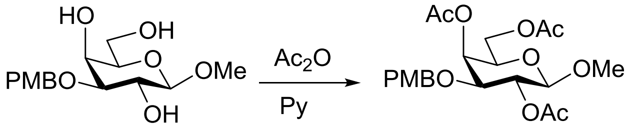

Acetic anhydride (450 µL, 4.8 mmol) was added to a solution of methyl 3-*O*-(4-methoxybenzyl)-*β*-D-galactopyranoside (300 mg, 0.95 mmol) in pyridine (2 mL) at 0 °C. The mixture was stirred at room temperature for 2 h, and then poured onto cold saturated $NaHCO_3$ solution. The aqueous phase was extracted with dichloromethane (3×10 mL). The combined organic phase was washed with saturated $NaHCO_3$ solution (1×15 mL), dried with anhydrous $MgSO_4$, and concentrated in vacuo. The residue was purified by silica gel flash chromatography to afford **27** as colorless oil (elution with ethyl acetate/petroleum ether =1:4 (v/v); 387 mg, 92% yield). ^{1}H NMR (400 MHz, $CDCl_3$) δ 7.19 (d, *J* = 8.6 Hz, 2H, Ar-H), 6.86 (d, *J* = 8.6 Hz, 2H, Ar-H), 5.49 (dd, *J* = 3.5, 1.2 Hz, 1H, H-4), 5.09 (dd, *J* = 10.0, 8.0 Hz, 1H, H-2), 4.62 (d, *J* = 11.9 Hz, 1H, $PhCH_2$), 4.33 (d, *J* = 11.9 Hz, 1H, $PhCH_2$), 4.29 (d, *J* = 8.1 Hz, 1H, H-1), 4.18 (d, *J* = 6.6 Hz, 2H, H-6), 3.81 (s, 4H, OMe, H-5), 3.52 (dd, *J* = 10.0, 3.4 Hz, 1H, H-2), 3.48 (s, 3H, OMe), 2.16 (s, 3H, OAc), 2.09 (s, 3H, OAc), 2.04 (s, 3H, OAc). ^{13}C NMR (100 MHz, $CDCl_3$) δ 170.58, 170.46, 169.48, 159.34, 129.48, 129.46, 113.75, 102.03, 76.11, 70.92, 70.86, 70.37, 65.88, 61.99, 56.73, 55.28, 20.97, 20.87, 20.78. HRMS (ESI-TOF) m/z $[M + Na]^+$ calcd for $[C_{12}H_{28}O_{10}Na]^+$: 463.1580; found: 463.1598.

Methyl 2,4,6-tri-*O*-acetyl-3-*O*-(*p*-methoxybenzyl)-*α*-D-mannopyranoside 29

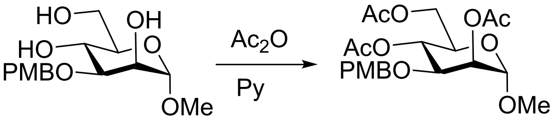

Methyl 2,4,6-tri-*O*-acetyl-3-*O*-(*p*-methoxybenzyl)-*α*-D-mannopyranoside **29** was synthesized according to the same procedure as the synthesis of **27**. $[\alpha]_D^{25}$ = +13.8 (c 0.79, CH_2Cl_2). ^{1}H NMR (400 MHz, $CDCl_3$) δ 7.18 (d, *J* = 8.6 Hz, 2H, Ar-H), 6.90 – 6.82 (m, 2H, Ar-H), 5.35 – 5.30 (m, 1H, H-2), 5.19 (t, *J* = 9.9 Hz, 1H, H-4), 4.73 (d, *J* = 1.8 Hz, 1H, H-1), 4.57 (d, *J* = 11.8 Hz, 1H, $PhCH_2$), 4.34 (d, *J* = 11.8 Hz, 1H, $PhCH_2$), 4.24 (dd, *J* = 12.2, 5.7 Hz, 1H, H-6a), 4.10 (dd, *J* = 12.2, 2.4 Hz, 1H, H-6b), 3.89-3.81 (m, 2H, H-3, H-5), 3.80 (s, 3H, OMe), 3.37 (s, 3H, OMe), 2.15 (s, 3H, OAc), 2.09 (s, 3H, OAc), 2.01 (s, 3H, OAc). ^{13}C NMR (100 MHz, $CDCl_3$) δ 170.74, 170.34, 169.71, 159.27, 129.73, 129.38, 113.73, 98.85, 73.91, 70.95, 68.51, 68.11, 67.41, 62.79, 55.22, 21.00, 20.81, 20.73. HRMS (ESI-TOF) m/z $[M + Na]^+$ calcd for $[C_{12}H_{28}O_{10}Na]^+$: 463.1580; found: 463.1593.

Methyl 2-*O*-(*p*-methoxybenzyl)-3-*O*-benzyl-4,6-*O*-benzylidene-*α*-D-glucopyranoside 31

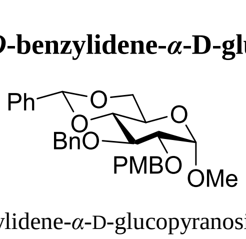

Methyl 2-*O*-(*p*-methoxybenzyl)-3-*O*-benzyl-4,6-di-*O*-benzylidene-*α*-D-glucopyranoside **31** was synthesized as previously reported.[10] ^{1}H NMR (400 MHz, $CDCl_3$) δ 7.49 (dd, *J* = 7.2, 2.3 Hz, 2H), 7.42 – 7.22 (m, 10H), 6.86 (d, *J* = 8.2 Hz, 2H), 5.54 (s, 1H), 4.90 (d, *J* = 11.3 Hz, 1H), 4.87 – 4.75 (m, 2H), 4.63 (d, *J* = 11.9 Hz, 1H), 4.53 (d, *J* = 3.6 Hz, 1H), 4.26 (dd, *J* = 10.0, 4.7 Hz, 1H), 4.02 (t, *J* = 9.3 Hz, 1H), 3.80 (s, 4H), 3.70 (t, *J* = 10.2 Hz, 1H), 3.59 (t, *J* = 9.4 Hz, 1H), 3.53 (dd, *J* = 9.3, 3.7 Hz, 1H), 3.39 (s, 3H).

Methyl 2-*O*-(*p*-methoxybenzyl)-3-*O*-(*tert*-butyl-dimethylsilyl)-4,6-*O*-benzylidene-*α*-D-glucopyranoside 33

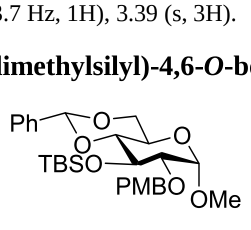

Methyl 3-*O*-(tert-butyl-dimethylsilyl)-4,6-*O*-benzylidene-*α*-D-glucopyranoside (200 mg, 0.5 mmol) was dissolved in DMF (2 mL) and the solution

was cooled to 0 °C. NaH (40 mg, 1.0 mmol) and *p*-methoxybenzyl chloride (102 μL, 0.75 mmol) was added to the mixture slowly. The mixture was then warmed to room temperature and stirred for 3 h. MeOH (1.0 mL) was added to quench the reaction, and the mixture was stirred for a further 10 min. The reaction mixture was diluted with water and extracted with CH_2Cl_2. The organic layers were combined and washed with water and brine sequentially, dried over anhydrous $MgSO_4$, concentrated in vacuo. The residue was purified by silica gel chromatography to give **33** (216 mg, 83%) as a colourless oil. $[\alpha]_D^{12}$ = 75.3 (c 0.15, CH_2Cl_2) ^{1}H NMR (600 MHz, $CDCl_3$) δ 7.45 (dq, *J* = 5.0, 2.8 Hz, 2H, Ar-H), 7.38 – 7.22 (m, 5H, Ar-H), 6.89 – 6.82 (m, 2H, Ar-H), 5.44 (s, 1H, PhCH), 4.75 (d, *J* = 12.0 Hz, 1H, $PhCH_2$), 4.53 – 4.40 (m, 1H, $PhCH_2$), 4.34 (d, *J* = 3.7 Hz, 1H, H-1), 4.19 (dd, *J* = 9.9, 4.6 Hz, 1H, H-6a), 4.05 (t, *J* = 9.0 Hz, 1H, H-4), 3.78 (s, 3H), 3.73 (dt, *J* = 9.7, 5.0 Hz, 1H, H-6b), 3.63 (t, *J* = 10.2 Hz, 1H, H-5), 3.44 – 3.30 (m, 2H, H-2, H-3), 3.31 (s, 3H), 0.86 (s, 9H), 0.08 (s, 3H), -0.00 (s, 3H). ^{13}C NMR (100 MHz, $CDCl_3$) δ 159.44, 137.37, 130.38, 129.92, 128.91, 128.08, 126.26, 113.84, 101.85, 99.40, 82.51, 79.86, 73.70, 71.48, 69.09, 62.13, 55.31, 55.27, 25.88, 18.31, -4.39, -4.49. HRMS (ESI-TOF) m/z $[M + Na]^+$ calcd for $[C_{28}H_{40}O_7SiNa]^+$: 539.2441; found: 539.2420.

1,2:5,6-Di-*O*-isopropylidene-3-*O*-(*p*-methoxybenzyl)-*α*-D-glucofuranose 35

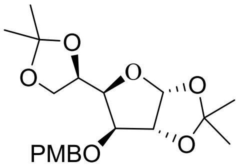

1,2:5,6-Di-*O*-isopropylidene-3-*O*-(*p*-methoxybenzyl)-*α*-D-glucofuranose **35** was prepared using literature procedure.[11] ^{1}H NMR (400 MHz, $CDCl_3$) δ 7.33 – 7.19 (m, 3H), 6.87 (d, *J* = 8.5 Hz, 2H), 5.88 (d, *J* = 3.7 Hz, 1H), 4.70 – 4.47 (m, 3H), 4.34 (dt, *J* = 7.7, 6.1 Hz, 1H), 4.23 – 4.03 (m, 2H), 4.00 (d, *J* = 2.8 Hz, 1H), 3.80 (s, 3H), 1.49 (s, 3H), 1.43 (s, 3H), 1.38 (s, 3H), 1.31 (s, 3H).

1,2:3,4-Di-*O*-isopropylidene-6-*O*-(*p*-methoxybenzyl)-*α*-D-galactopyranose 37

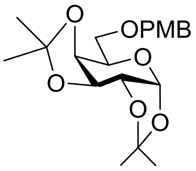

1,2:3,4-Di-*O*-isopropylidene-6-*O*-(*p*-methoxybenzyl)-*α*-D-galactopyranose **37** was synthesized according to the same procedure as the synthesis of **31**.[12] ^{1}H NMR (400 MHz, $CDCl_3$) δ 7.27 (d, *J* = 8.5 Hz, 2H), 6.87 (d, *J* = 8.4 Hz, 2H), 5.54 (d, *J* = 5.0 Hz, 1H), 4.66 – 4.44 (m, 3H), 4.29 (ddd, *J* = 18.2, 6.5, 2.1 Hz, 2H), 3.98 (td, *J* = 6.4, 1.9 Hz, 1H), 3.80 (s, 3H), 3.76 – 3.54 (m, 2H), 1.53 (s, 3H), 1.44 (s, 3H), 1.34 (s, 3H), 1.33 (s, 3H).

Methyl 2,3-di-*O*-benzyl-4,6-*O*-benzylidene-*α*-D-mannopyranoside 39

Methyl 2,3-di-*O*-benzyl-4,6-*O*-benzylidene-*α*-D-mannopyranoside **39** was synthesized according to the same procedure as the synthesis of **1a**.[13] ^{1}H NMR (600 MHz, $CDCl_3$) δ 7.55 – 7.26 (m, 15H), 5.64 (s, 1H), 4.81 (dd, *J* = 12.2, 7.0 Hz, 2H), 4.73 (d, *J* = 12.2 Hz, 1H), 4.69 (d, *J* = 1.6 Hz, 1H), 4.65 (d, *J* = 12.3 Hz, 1H), 4.29 – 4.21 (m, 2H), 3.94 (dd, *J* = 10.0, 3.3 Hz, 1H), 3.88 (t, *J* = 10.3 Hz, 1H), 3.83 (dd, *J* = 3.3, 1.6 Hz, 1H), 3.77 (dt, *J* = 9.9, 5.1 Hz, 1H), 3.31 (s, 3H).

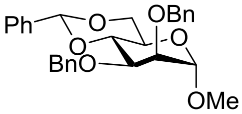

Methyl 2,3-di-*O*-benzyl-4,6-*O*-benzylidene-*β*-D-glucopyranoside 40

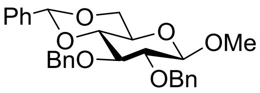

Methyl 2,3-di-*O*-benzyl-4,6-*O*-benzylidene-*β*-D-glucopyranoside **40** was synthesized according to the same procedure as the synthesis of **1a**.[14] ^{1}H NMR (600 MHz, $CDCl_3$) δ 7.57 – 7.23 (m, 15H), 5.57 (s, 1H), 4.89 (dd, *J* = 22.4, 10.9 Hz, 2H), 4.78 (dd, *J* = 23.8, 11.1 Hz, 2H), 4.42 (d, *J* = 7.7 Hz, 1H), 4.39 – 4.33 (m, 1H), 3.77 (dt, *J* = 19.5, 9.4 Hz, 2H), 3.68 (t, *J* = 9.3 Hz, 1H), 3.58 (s, 3H), 3.43 (m, 2H).

3. References

1. Liu, Y.; Zeng, J.; Sun, J. C.; Cai, L.; Zhao, Y. Q.; Fang, J.; Hu, B.; Shu, P. H.; Meng, L. K.; Wan, Q. 1,4-Dithiothreitol Mediated Cleavage of Acetal and Ketal Type of Diol Protecting Groups. *Org. Chem. Front*. **2018**, *5*, 2427−2431.

2. Johnsson, R.; Ohlin, M.; Ellervik, U. Reductive Openings of Benzylidene Acetals Revisited: A Mechanistic Scheme for Regio- and Stereoselectivity, *J. Org. Chem.* **2010**, *75*, 8003−8011.

3. Banerjee, A.; Senthilkumar, S.; Baskaran, S. Benzylidene acetal protecting group as a carboxylic acid surrogate: Synthesis of functionalized uronic acids and sugar amino acids. *Chem. Eur. J.* **2016**, *22*, 902−906.

4. Zhang, Y-J.; Dayoub, W.; Chen, G-R.; Lemaire, M. TMDS as a Dual-Purpose Reductant in the Regioselective Ring Cleavage of Hexopyranosyl Acetals to Ethers. *Eur. J. Org. Chem.* **2012**, 1960–1966.

5. Seitz, A.; Wende, R. C.; Roesner, E.; Niedek, D.; Topp, C. Colgan, A. C. McGarrigle, E. M.; Schreiner, P. R. Site-Selective Acylation of Pyranosides with Oligopeptide Catalysts. *J. Org. Chem*. **2021**, *86*, 3907−3922.

6. van der Vorm, S.; Hansen, T.; Overkleeft, H. S.; van der Marel, G. A.; Codée, J. D. C. The Influence of Acceptor Nucleophilicity on the Glycosylation Reaction Mechanism. *Chem. Sci.* **2017**, *8*, 1867−1875.

7. Maki, Y.; Nomura, K.; Okamoto, R.; Izumi, M.; Mizutani, Y.; Kajihara, Y. Acceleration and Deceleration Factors on the Hydrolysis Reaction of 4,6-*O*-Benzylidene Acetal Group. *J. Org. Chem.* **2020**, *85*, 15849−15856.

8. Emmadi, M.; Kulkarni, S. S. Synthesis of Rare Deoxy Amino Sugar Building Blocks Enabled the Total Synthesis of a Polysaccharide Repeating Unit Analogue from the LPS of *Psychrobacter cryohalolentis* $K5^T$. *J. Org. Chem.* **2018**, *83*, 14323−14337.

9. Ye, D. F.; Liu, Z. Y.; Chen, H.; Sessler, J. L.; Lei, C. H. Cesium Carbonate Catalyzed Esterification of N-Benzyl-N-Bocamides under Ambient Conditions. *Org. Lett*. **2019**, *21*, 6888−6892.

10. Bauder, C. A convenient synthesis of orthogonally protected 2-deoxystreptamine (2-DOS) as an aminocyclitol scaffold for the development of novel aminoglycoside antibiotic derivatives against bacterial resistance. *Org. Biomol. Chem.* **2008**, *6*, 2952−2960.

11. Raunak, Babu, B. R.；Sørensen, M. D. Parmar, V. S.; Harrit, N. H.; Wengel, J. Oligodeoxynucleotides containing α-L-ribo configured LNA-type C-aryl nucleotides. *Org. Biomol. Chem.* **2004**, *2*, 80−89.

12. Viuffa, A. H.; Heuckendorfa, M.; Jensen, H. H. *p*-Chlorobenzyl Ether: A *p*-Methoxybenzyl Ether in Disguise. *Org. Lett.* **2016**, *18*, 5773−5775.

13. Crich, D.; Banerjee, A.; Yao, Q-J. Direct Chemical Synthesis of the β-d-Mannans: The *β*-(1→2) and *β*-(1→4) Series.*J. Am. Chem. Soc.* **2004**, *126*, *45*, 14930–14934

14. Yoneda, Y.; Kawada, T.; Rosenau, T.; Kosma, P. Synthesis of methyl 4′-*O*-methyl-$^{13}C_{12}$-β-D-cellobioside from $^{13}C_6$-D-glucose. Part 1: Reaction optimization and synthesis. *Carbohydr. Res.* **2005**, *340*, 2428–2435

4. Copies of NMR spectra

Methyl 2,3-di-*O*-benzyl-4,6-*O*-benzylidene-*α*-D-glucopyranoside 1a

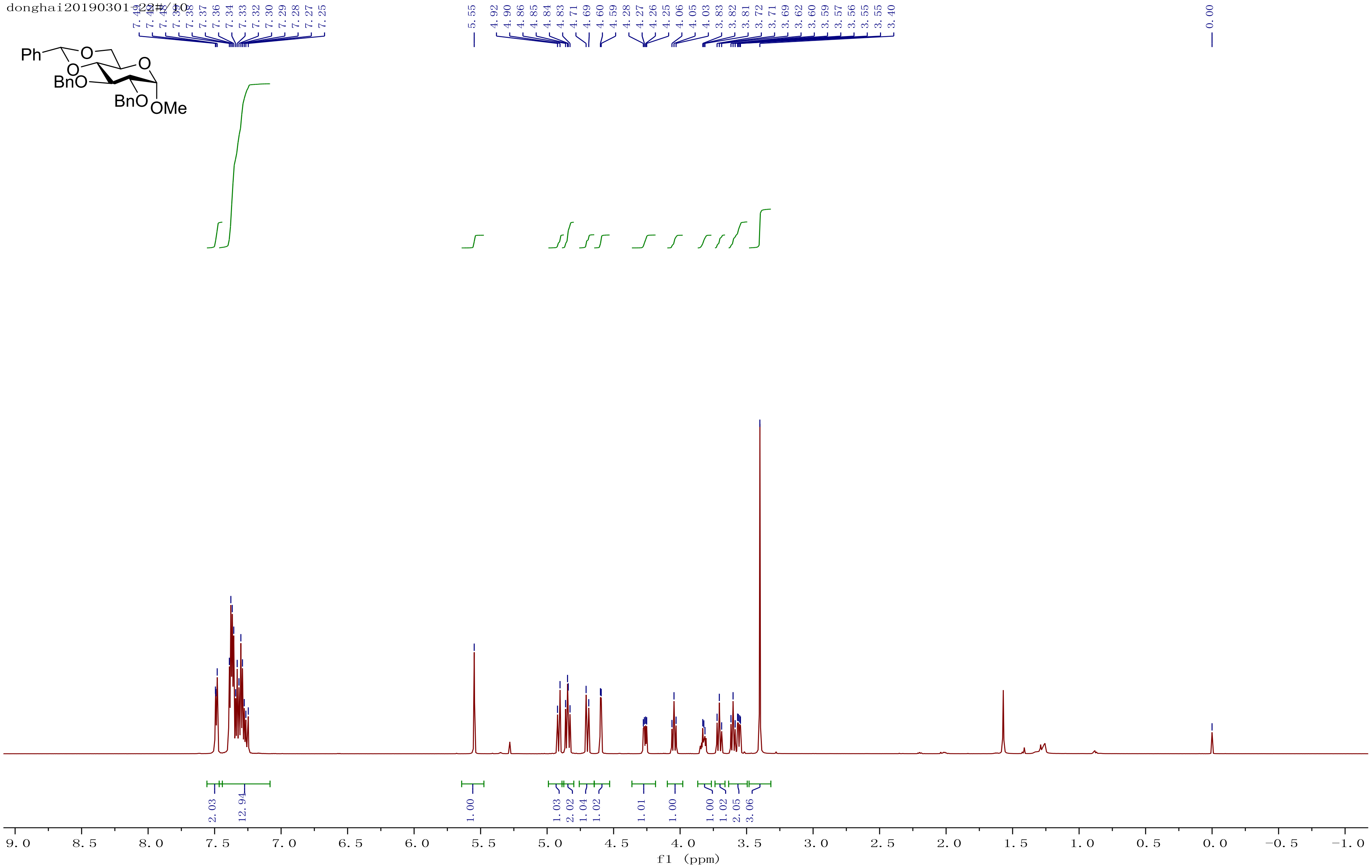

Methyl 2,3-di-*O*-benzyl-4,6-*O*-(*p*-methoxybenzylidene)-*α*-D-glucopyranoside 1b

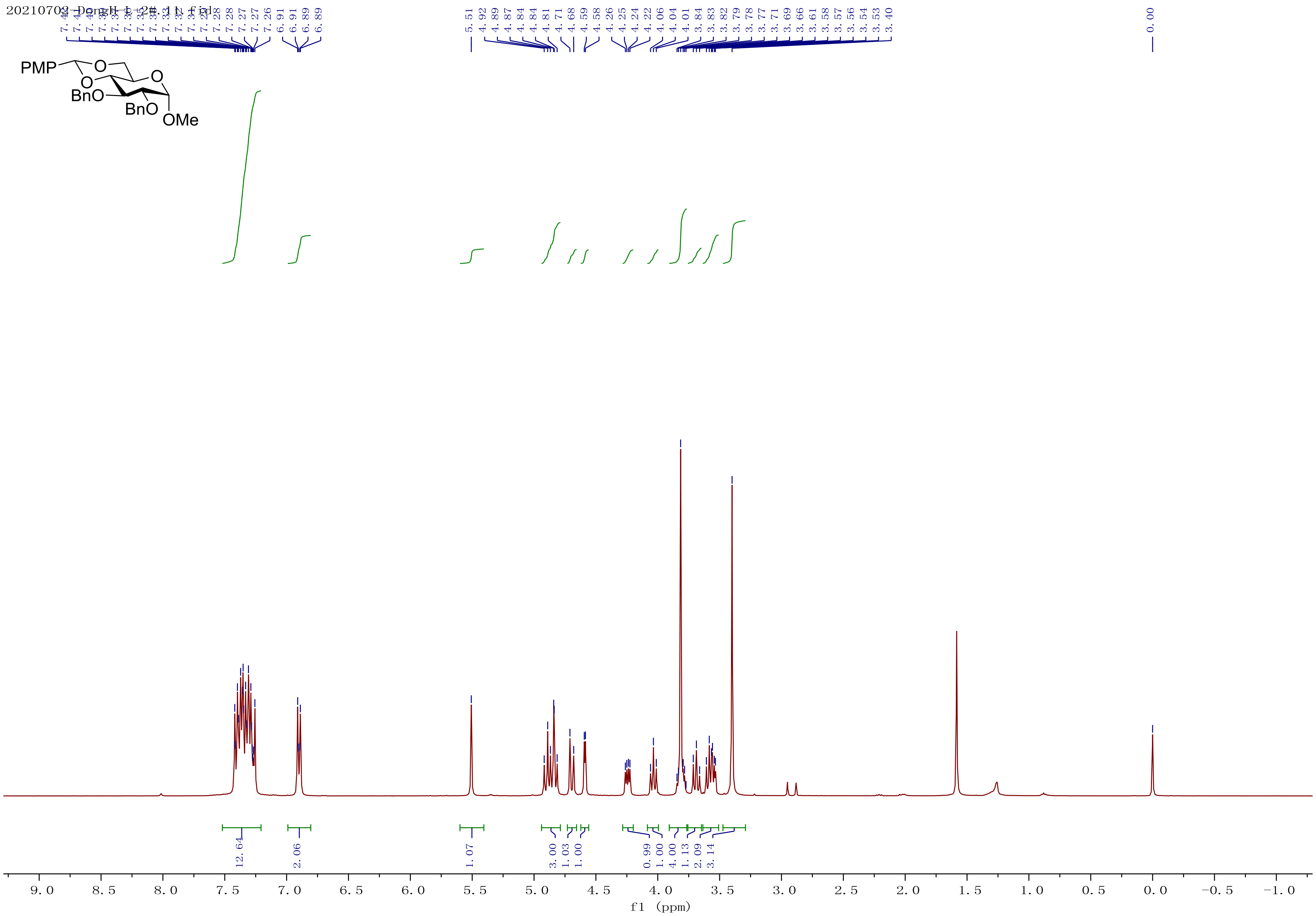

Methyl 2,3-di-*O*-benzyl-4,6-*O*-(*p*-bromobenzylidene)-*α*-D-glucopyranoside 1c

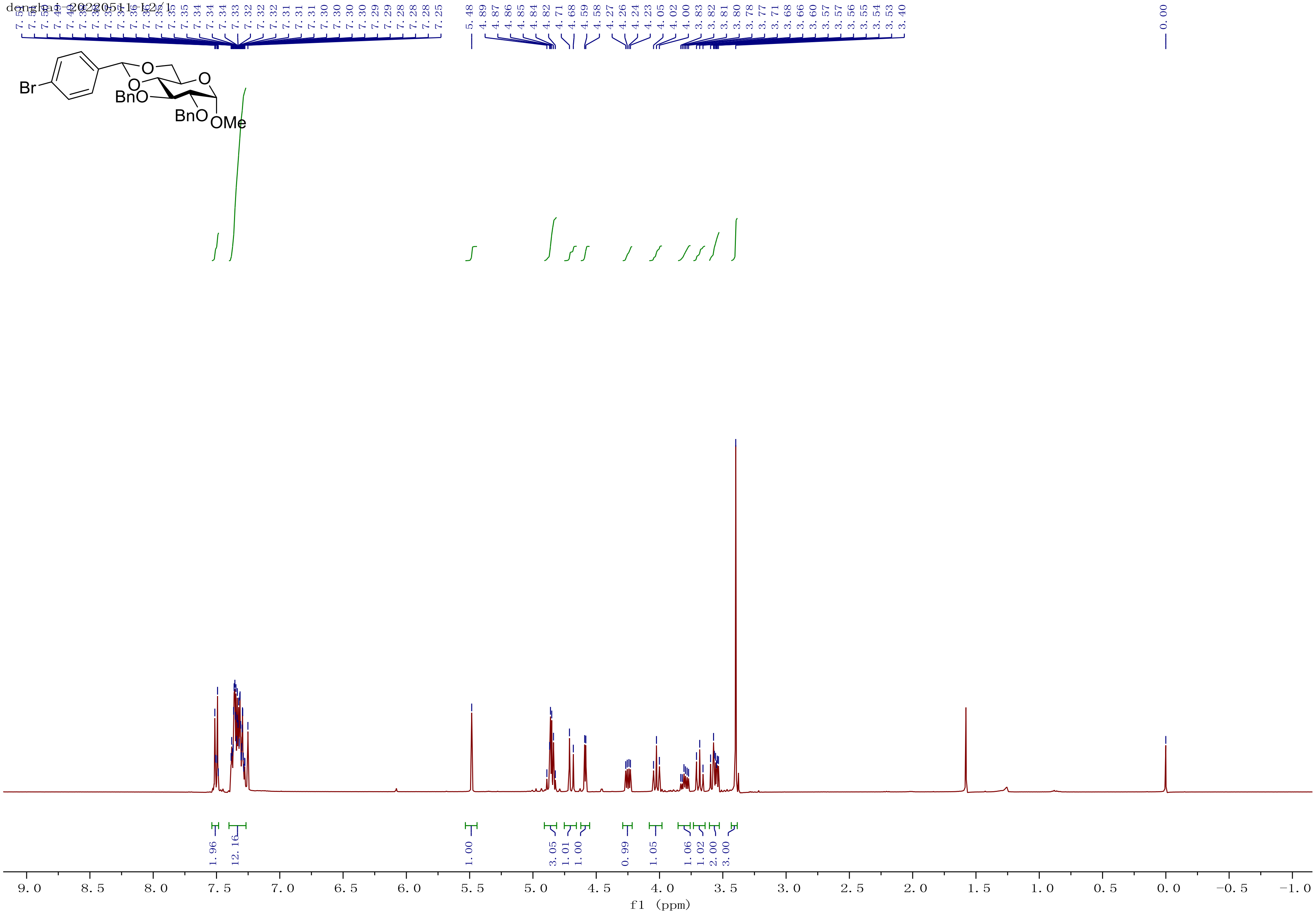

Methyl 2,3-di-*O*-benzyl-*α*-D-glucopyranoside 2

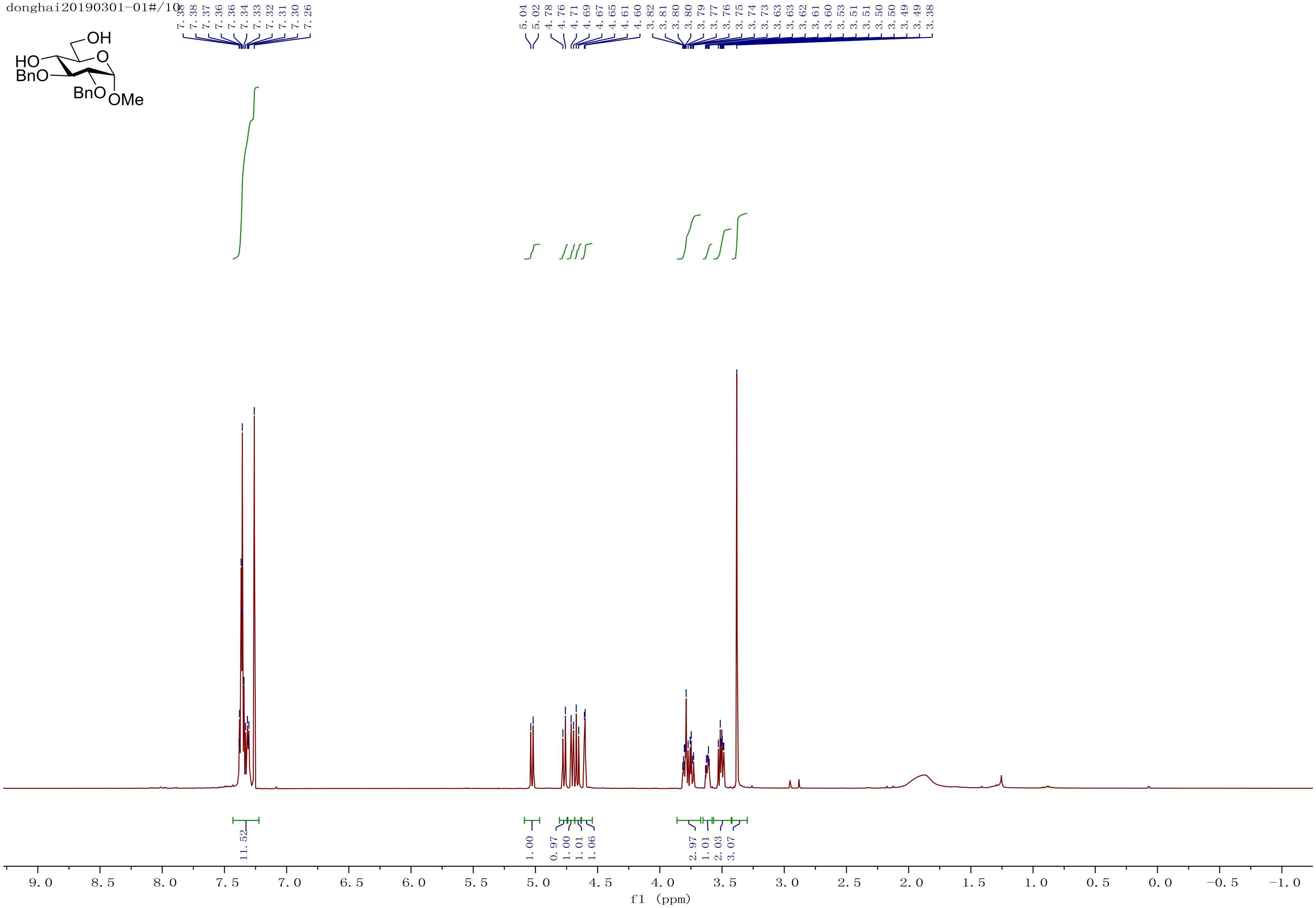

Methyl 2,3-di-*O*-benzyl-4,6-*O*-acetyl-*α*-D-glucopyranoside 2a

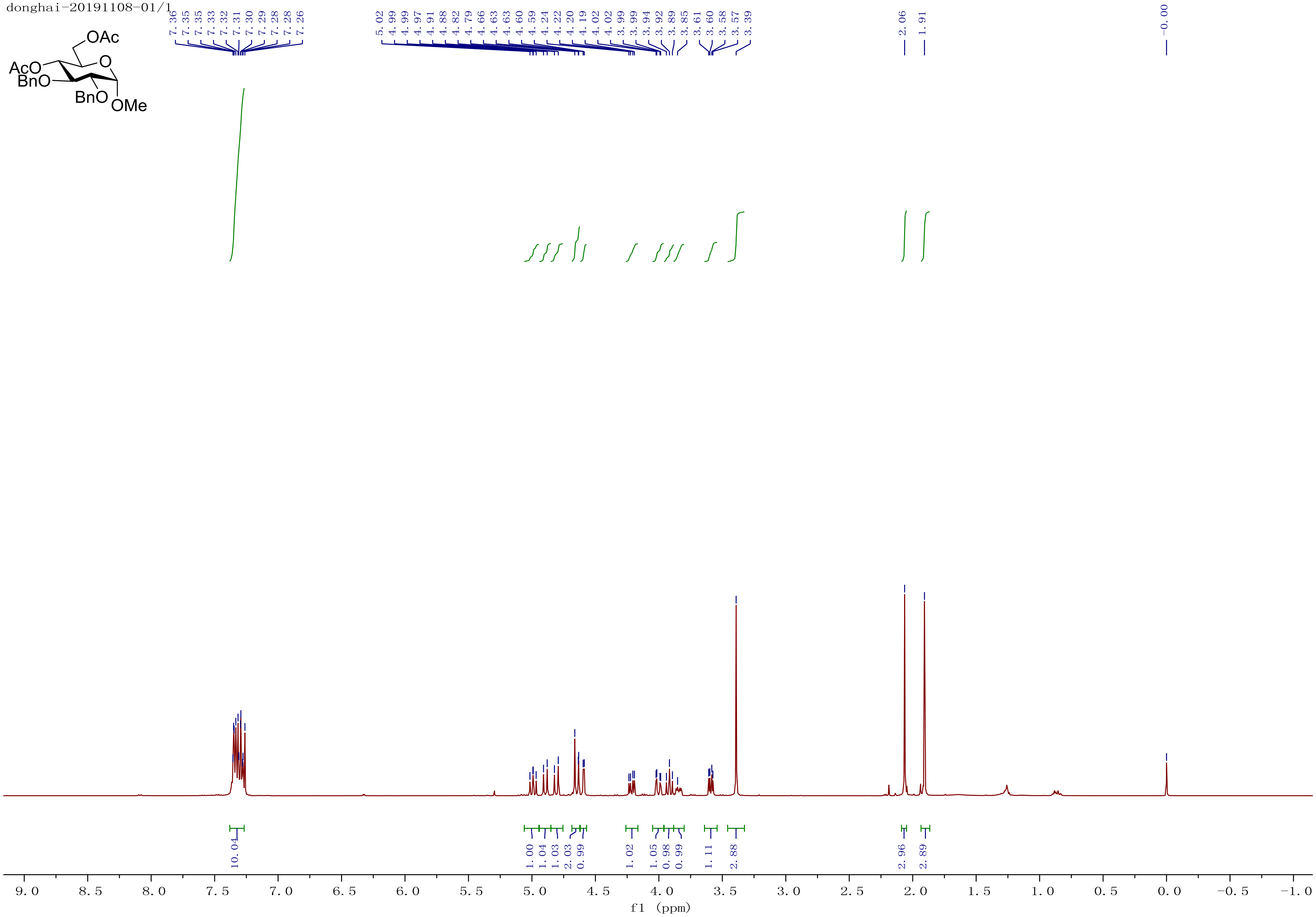

Methyl 2,3-di-*O*-methyl-4,6-*O*-benzylidene-*α*-D-glucopyranoside 3

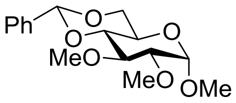

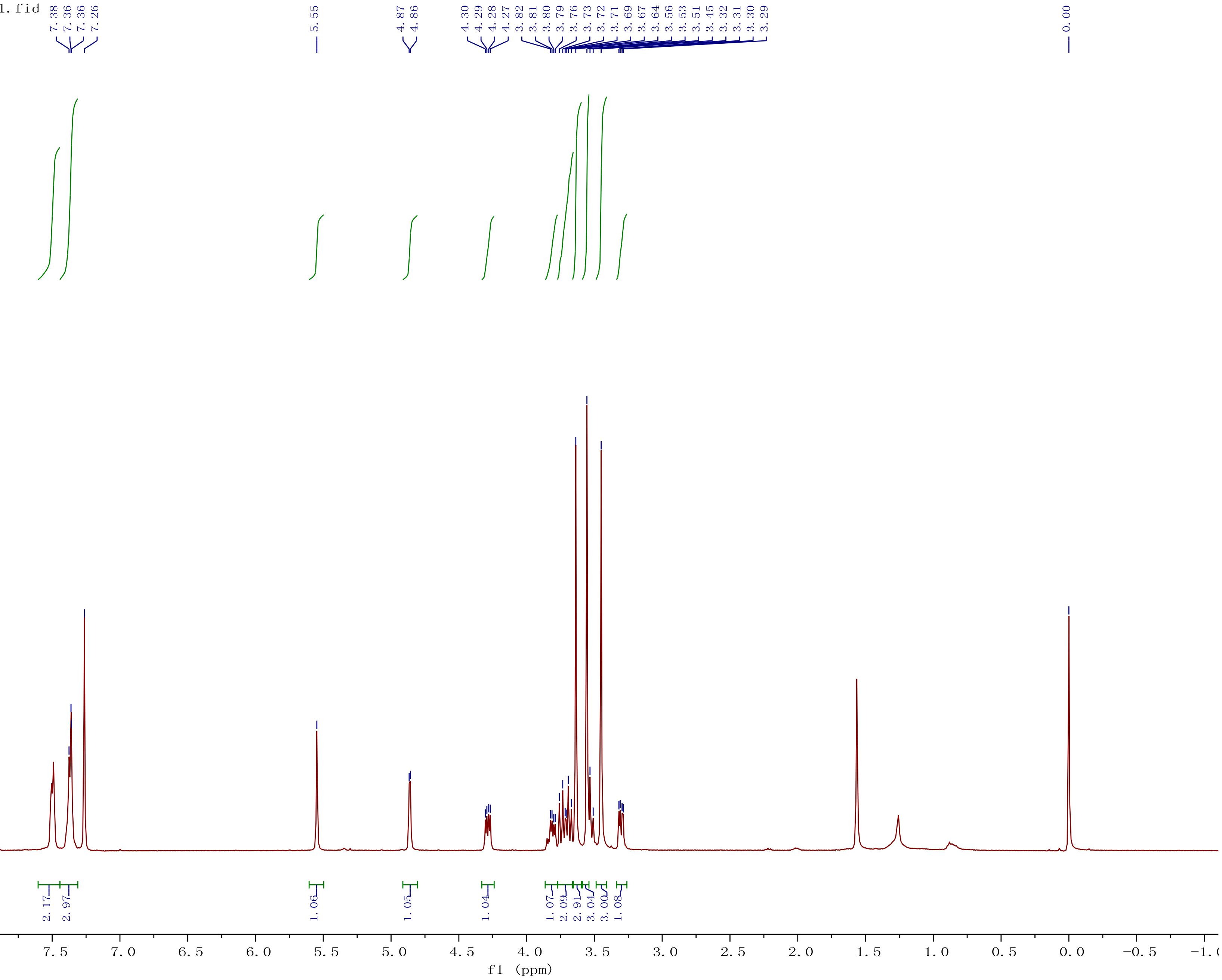

Methyl-2,3-di-*O*-methyl-*α*-D-glucopyranoside 4

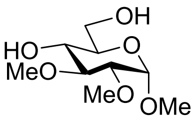

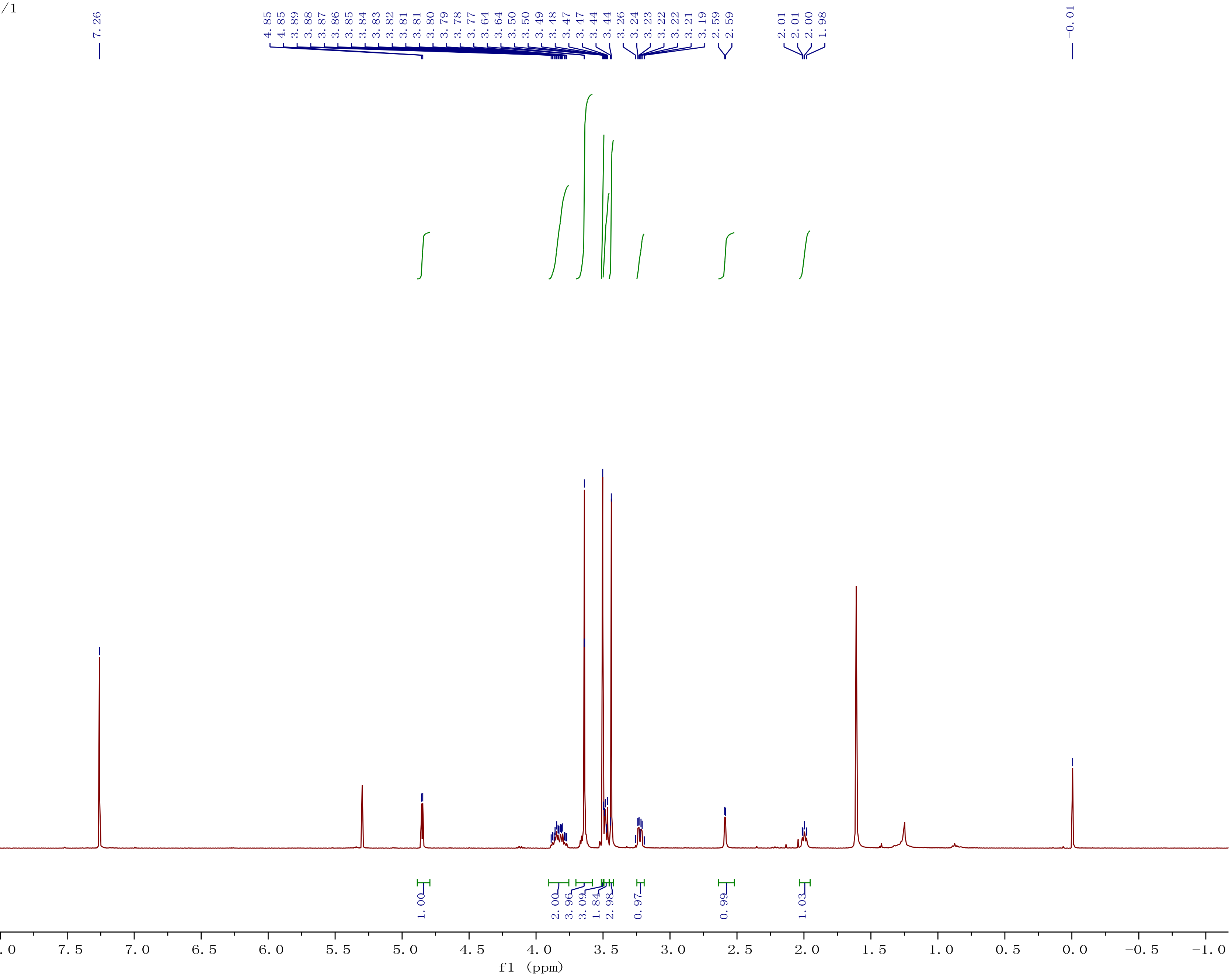

Methyl 2,3-di-*O*-acetyl-4,6-*O*-benzylidene-*α*-D-glucopyranoside 5

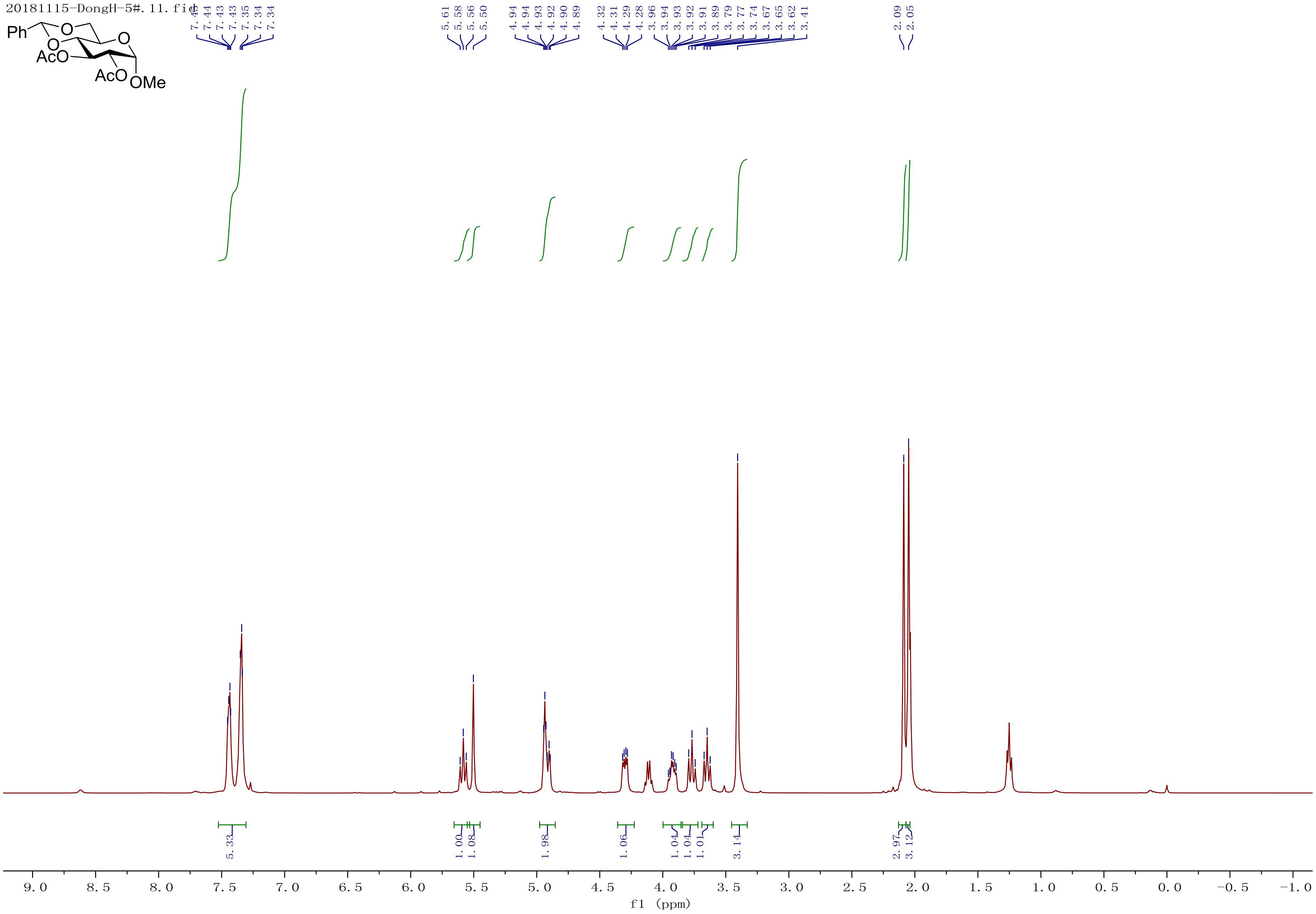

Methyl 2,3-di-*O*-acetyl-*α*-D-glucopyranoside 6

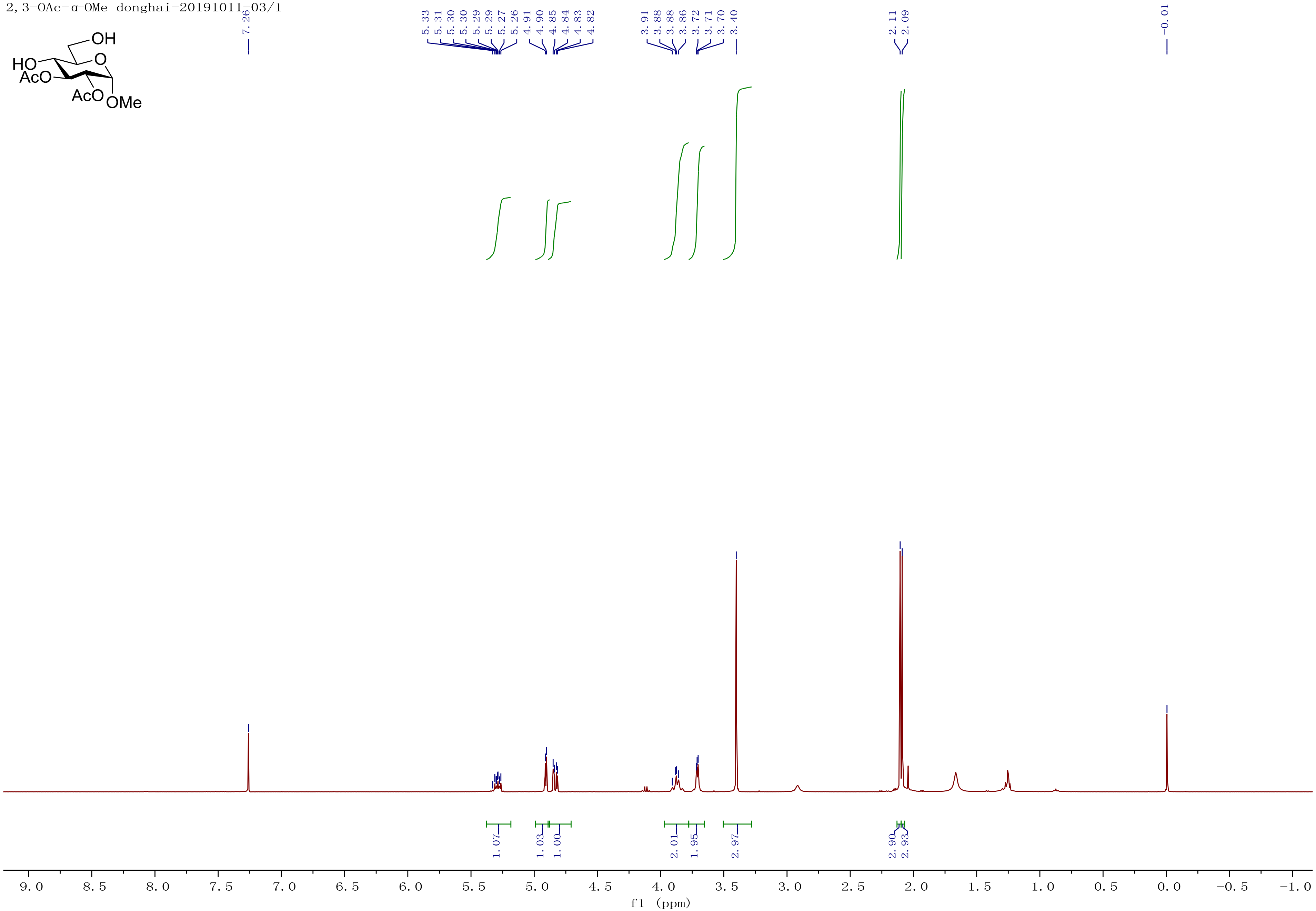

Methyl 2,3-di-*O*-pivaloyl-4,6-*O*-benzylidene-*α*-D-glucopyranoside 7

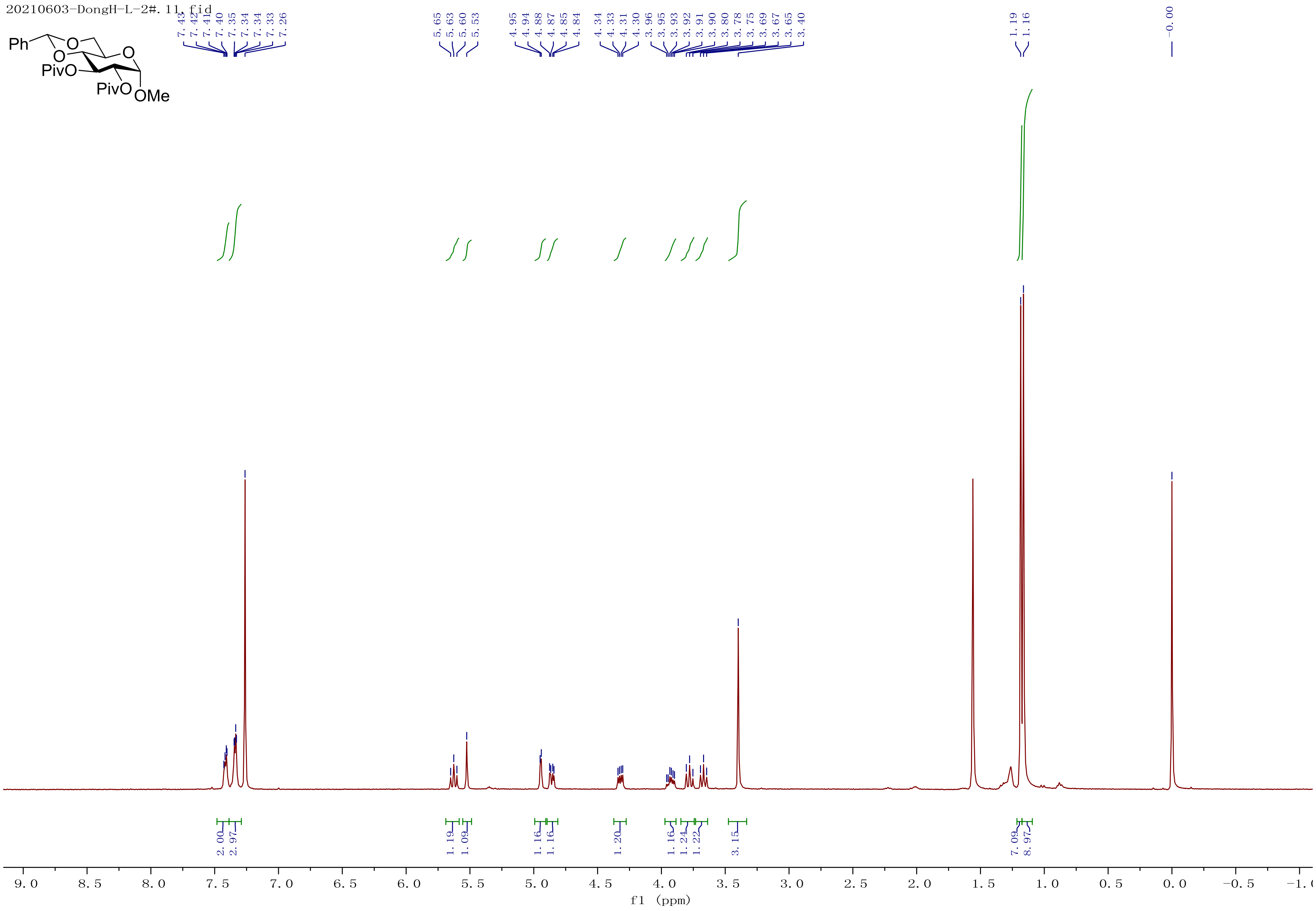

Methyl 2,3-di-*O*-pivaloyl-*α*-D-glucopyranoside 8

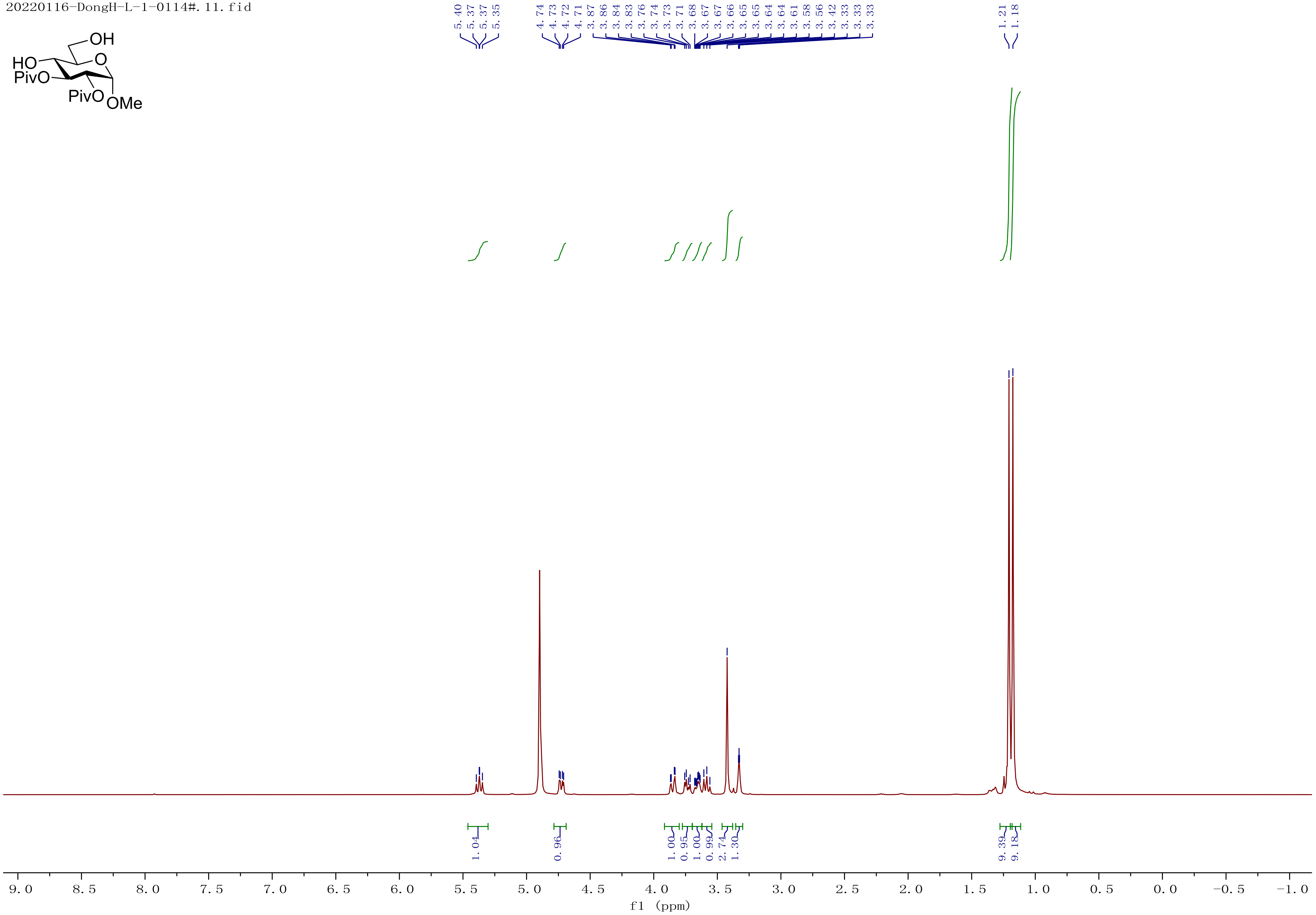

Methyl 2,3-di-*O*-benzoyl-4,6-*O*-benzylidene-*β*-D-glucopyranoside 9

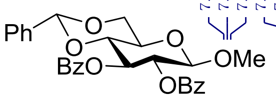

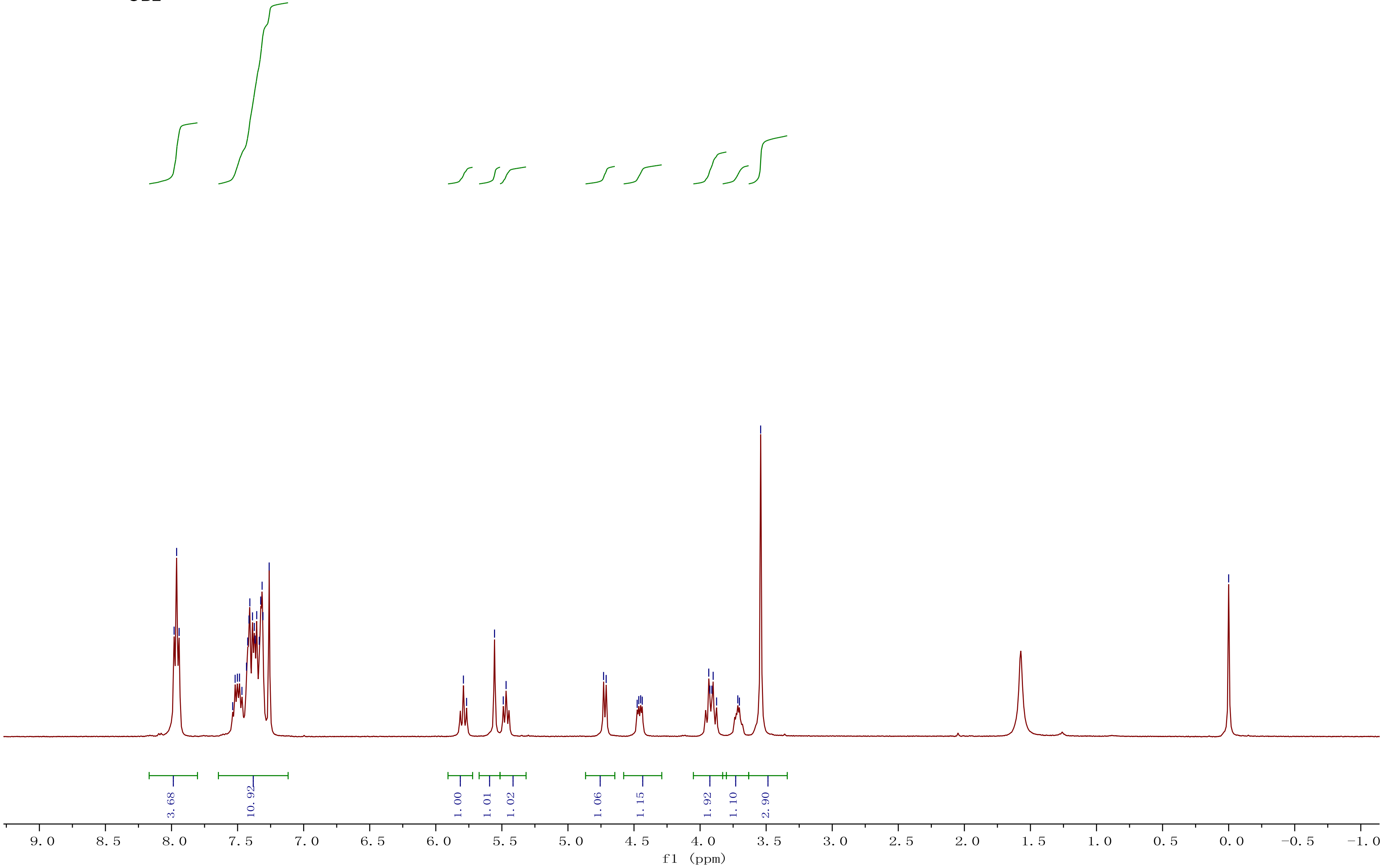

Methyl 2,3-di-*O*-benzoyl-*β*-D-glucopyranoside 10

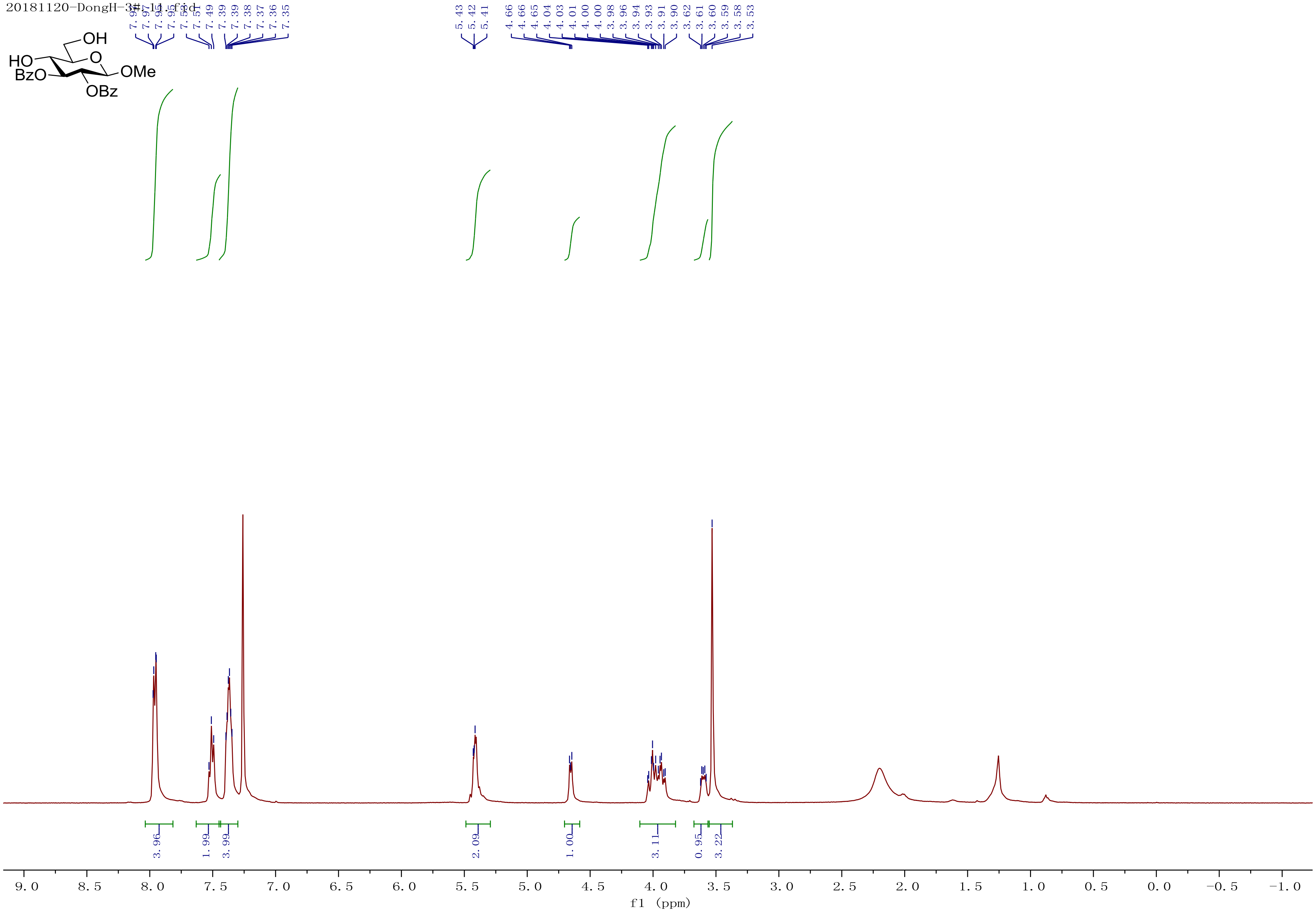

Benzyl 2,3-di-*O*-acetyl-4,6-*O*-benzylidene-1-thio-*β*-D-glucopyranoside 11

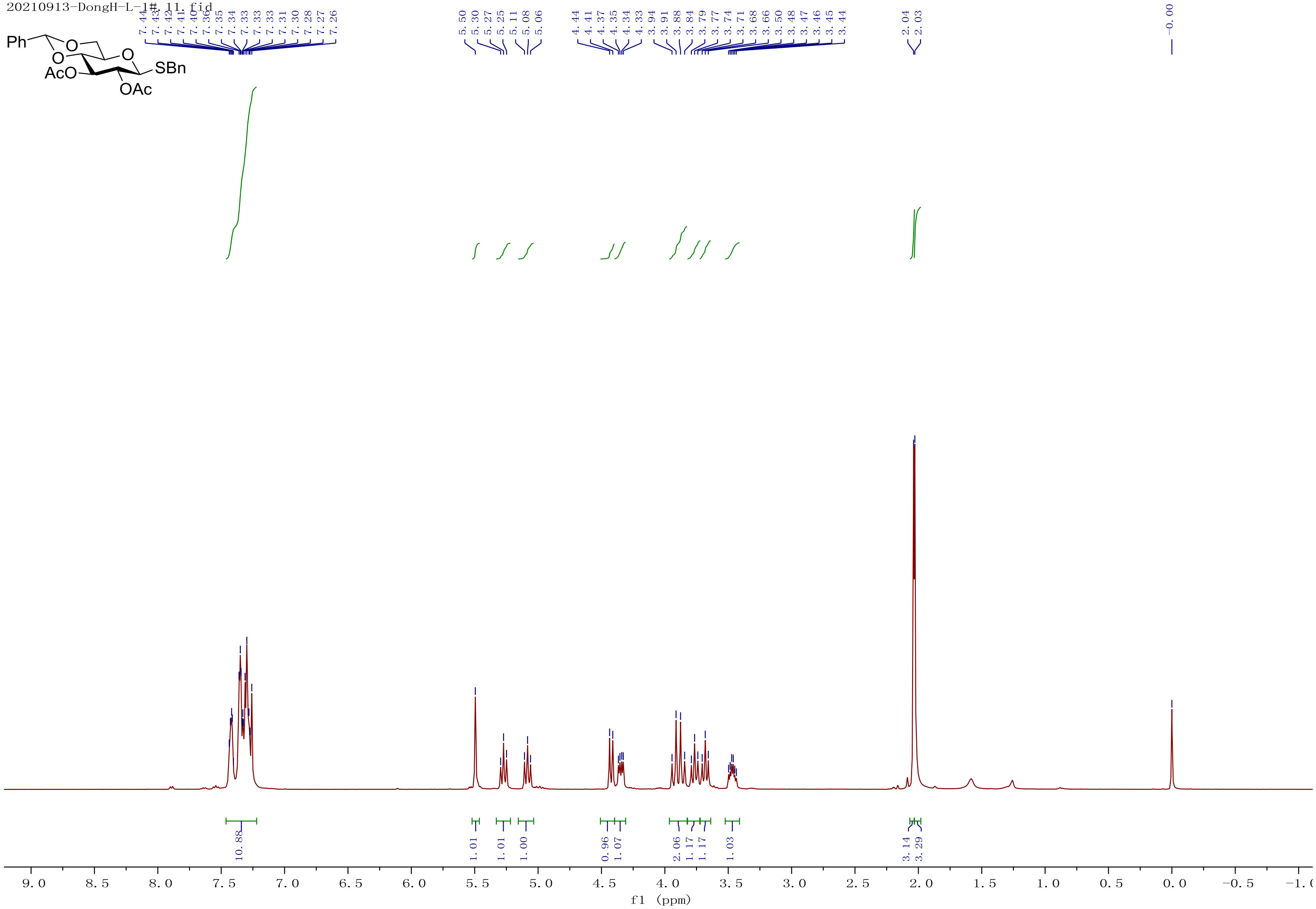

Benzyl 2,3-di-*O*-acetyl-4,6-*O*-benzylidene-1-thio-*β*-D-glucopyranoside 11

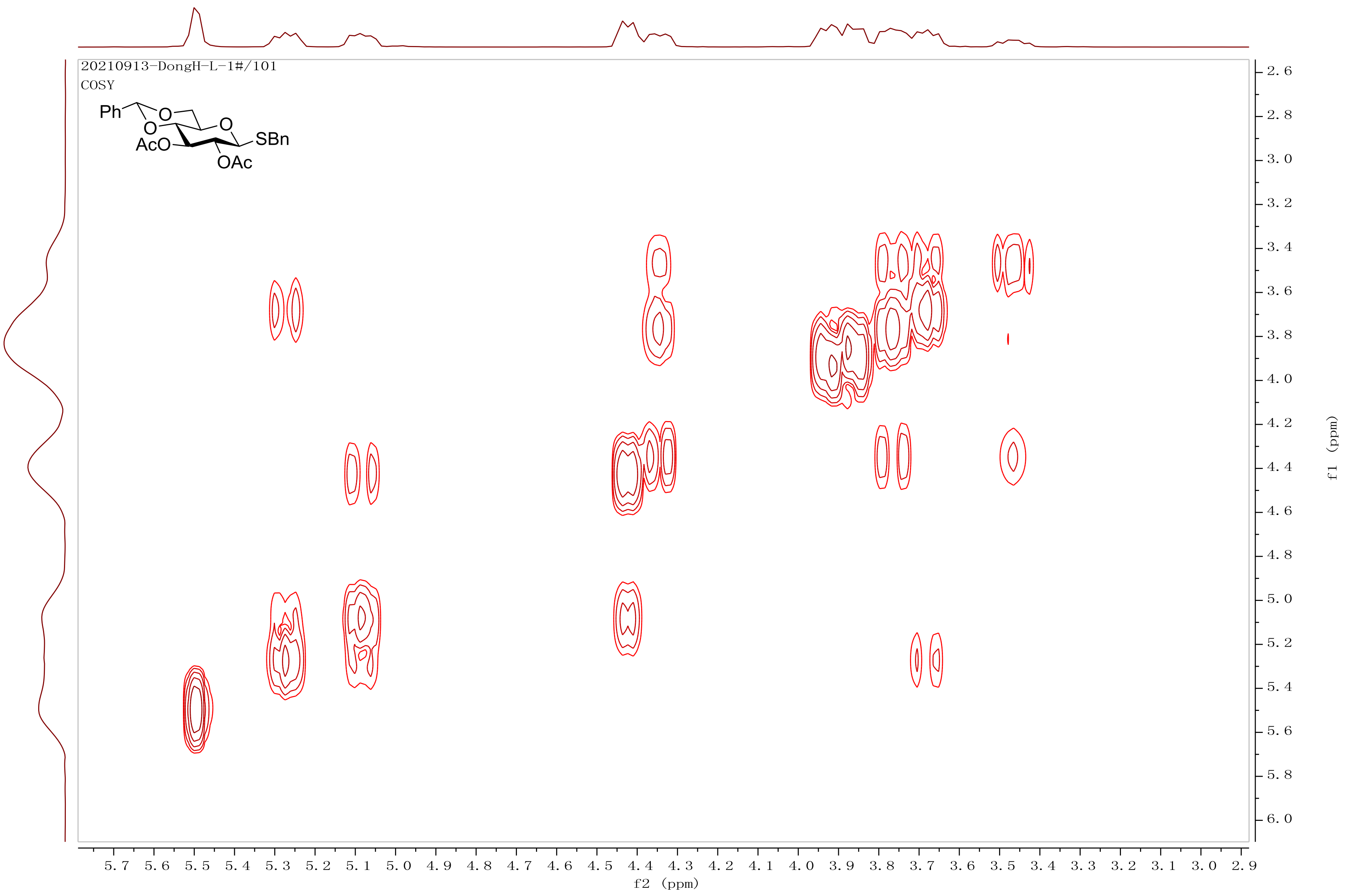

Benzyl 2,3-di-*O*-acetyl-4,6-*O*-benzylidene-1-thio-*β*-D-glucopyranoside 11

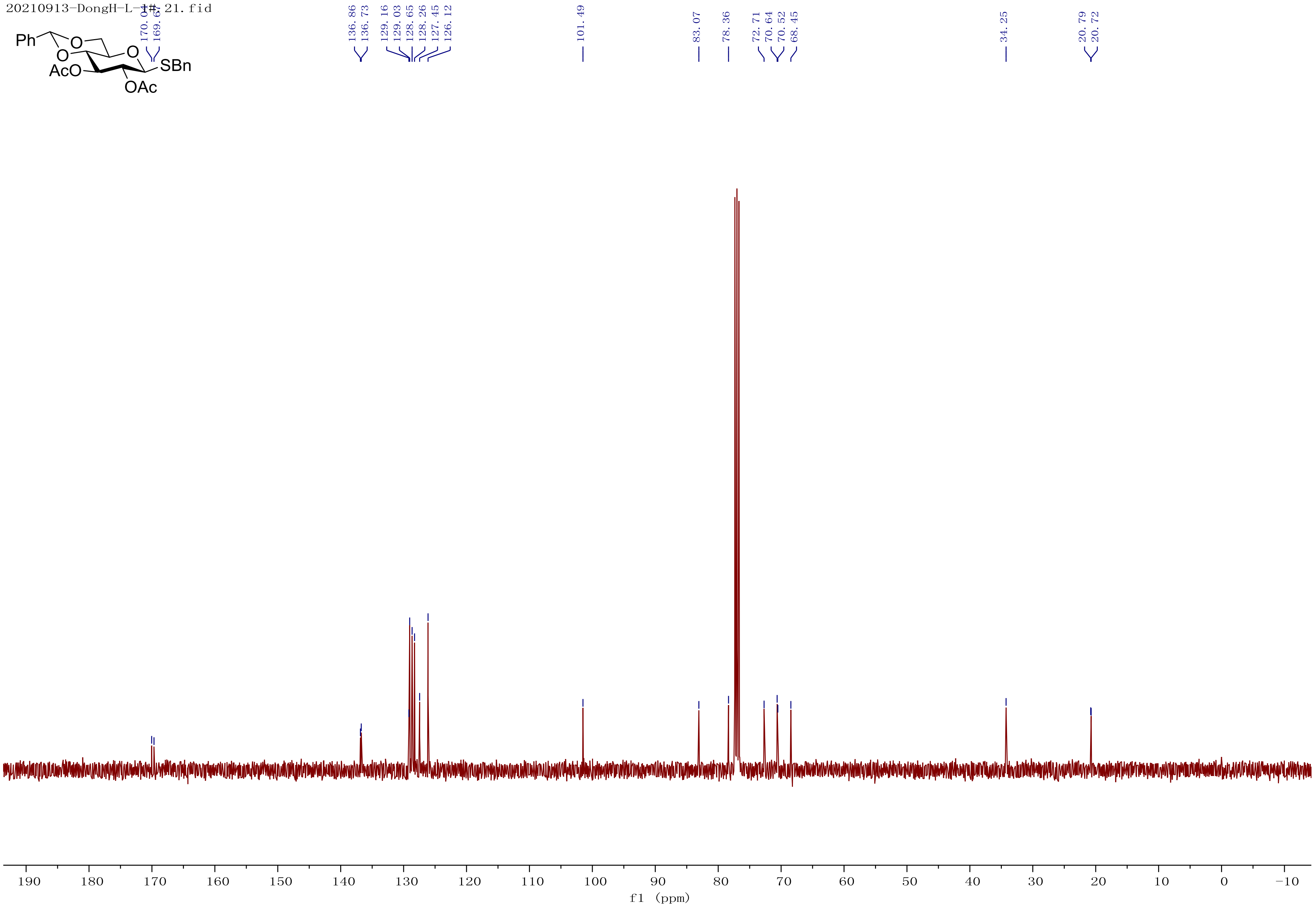

Benzyl 2,3-di-*O*-acetyl-1-thio-*β*-D-glucopyranoside 12

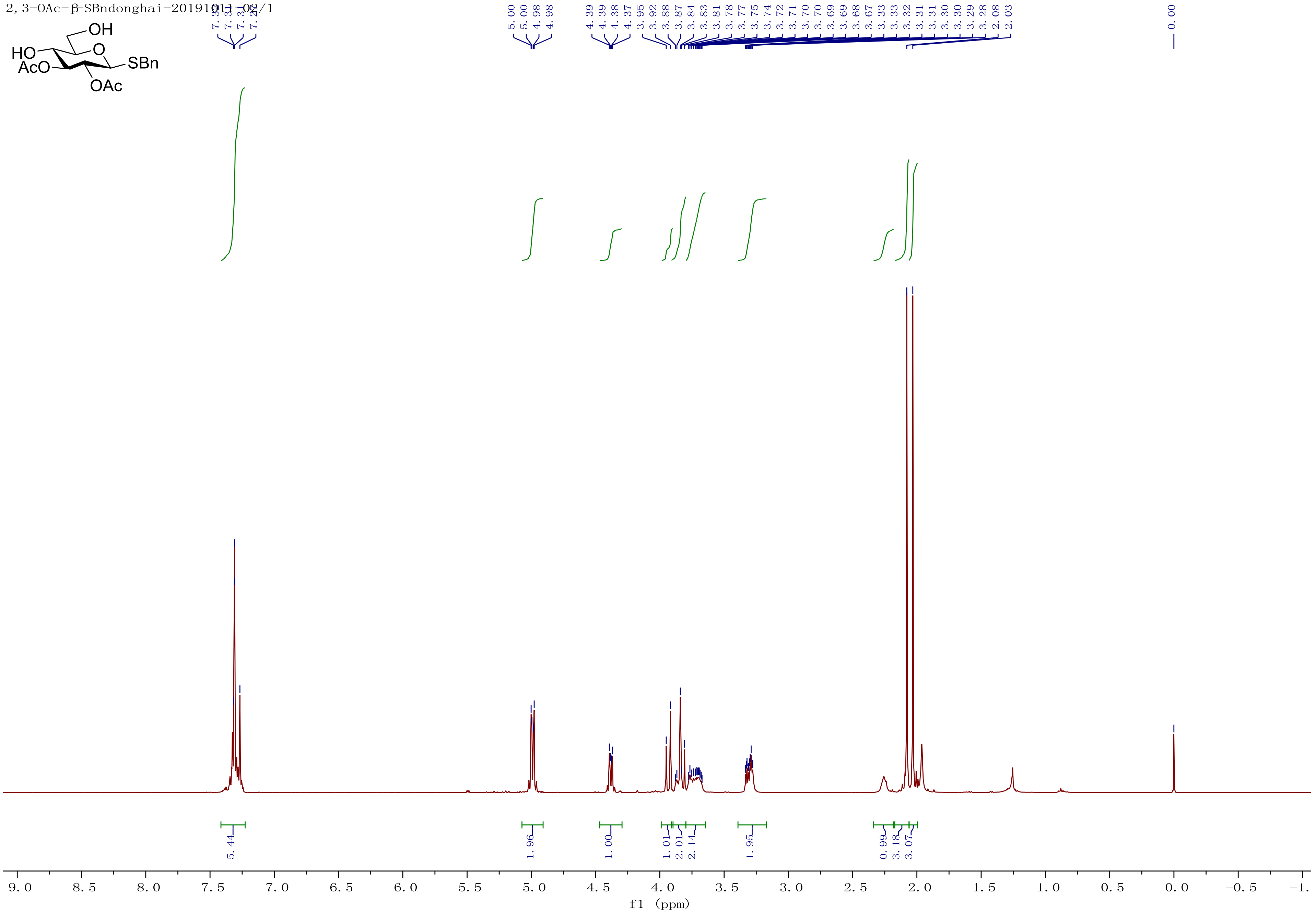

Benzyl 2,3-di-*O*-acetyl-1-thio-*β*-D-glucopyranoside 12

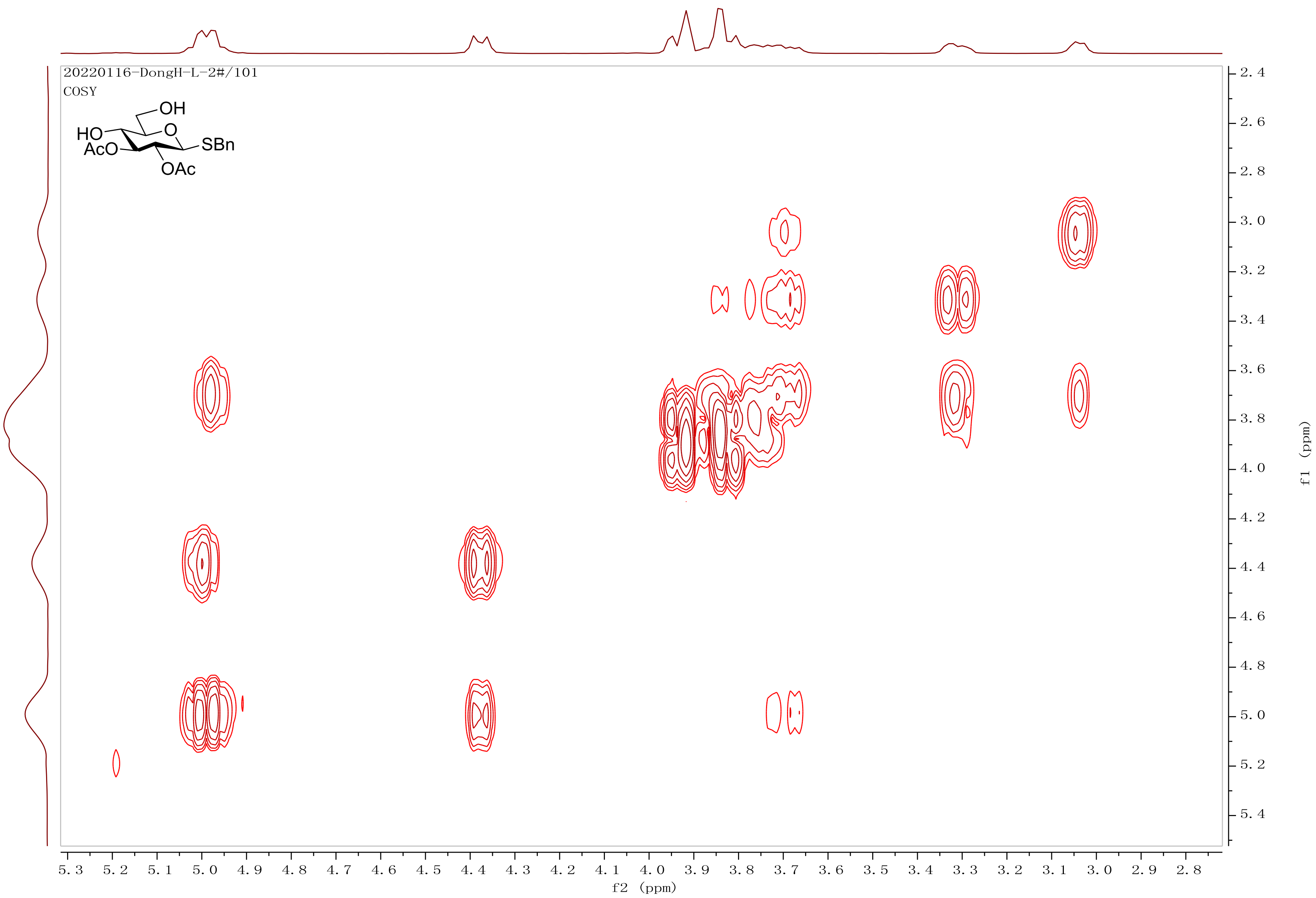

Benzyl 2,3-di-*O*-acetyl-1-thio-*β*-D-glucopyranoside 12

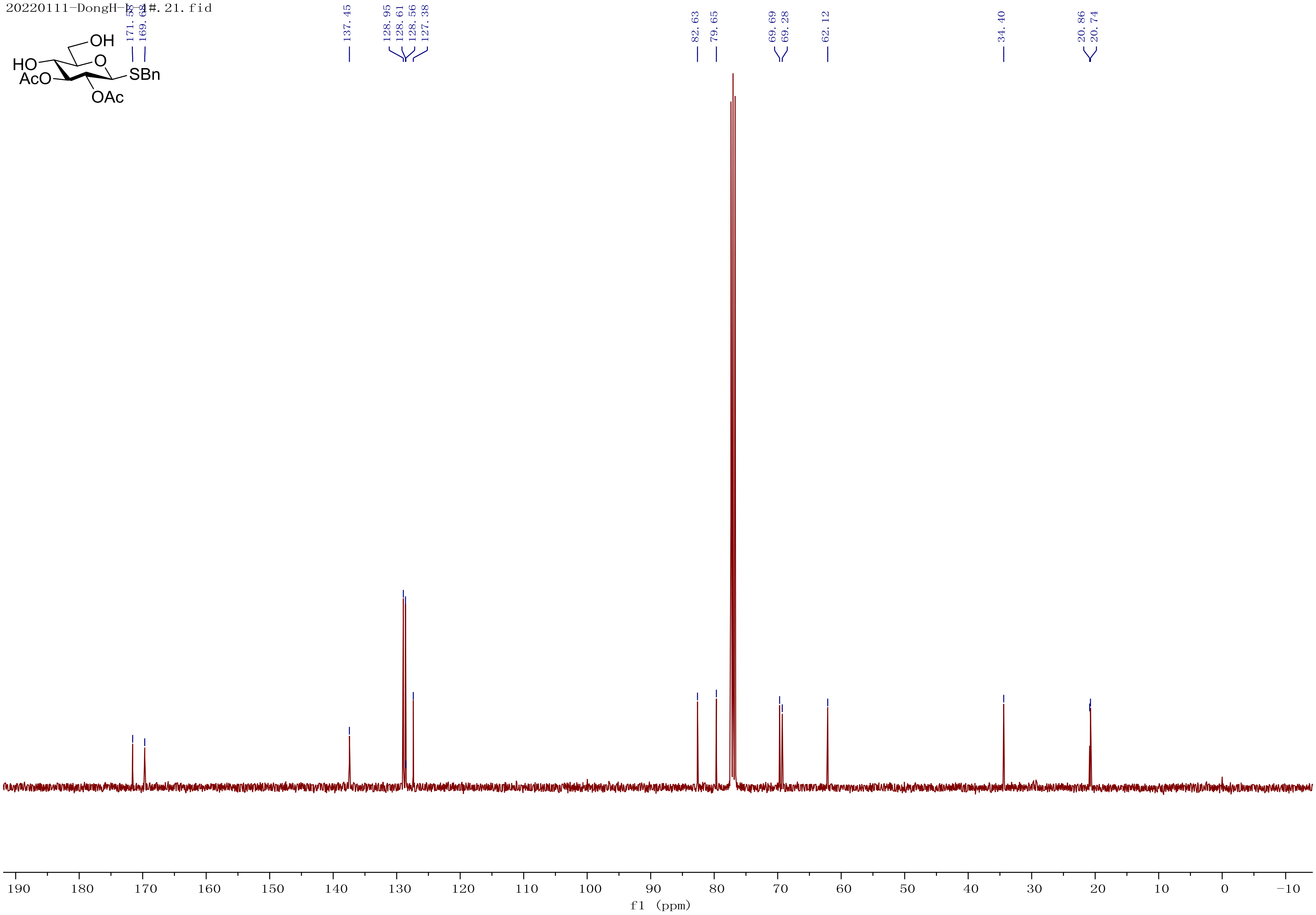

4-Methylphenyl 2,3-di-*O*-acetyl-4,6-*O*-benzylidene-1-thio-*β*-D-glucopyranoside 13

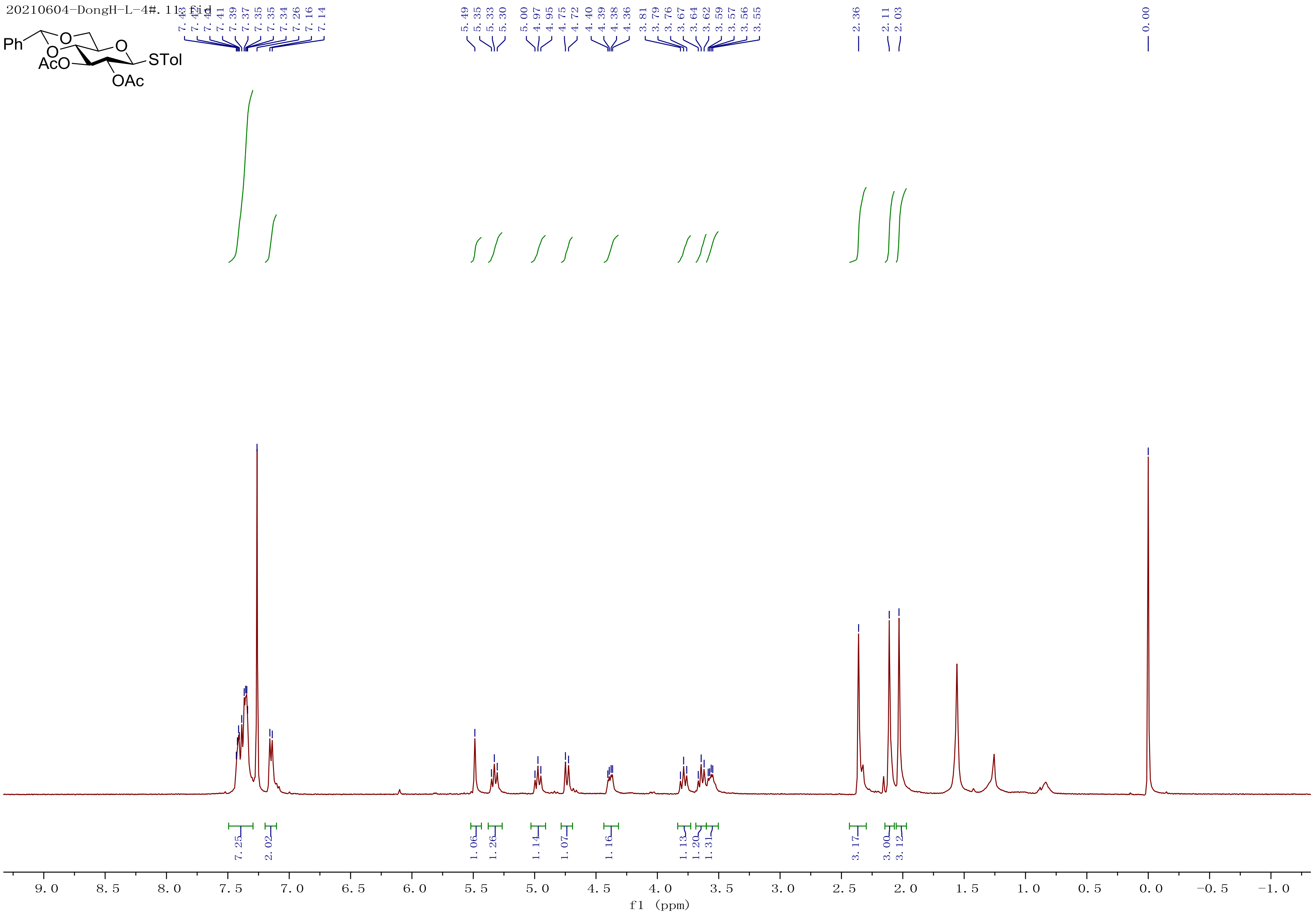

4-Methylphenyl 2,3-di-*O*-acetyl-1-thio-*β*-D-glucopyranoside 14

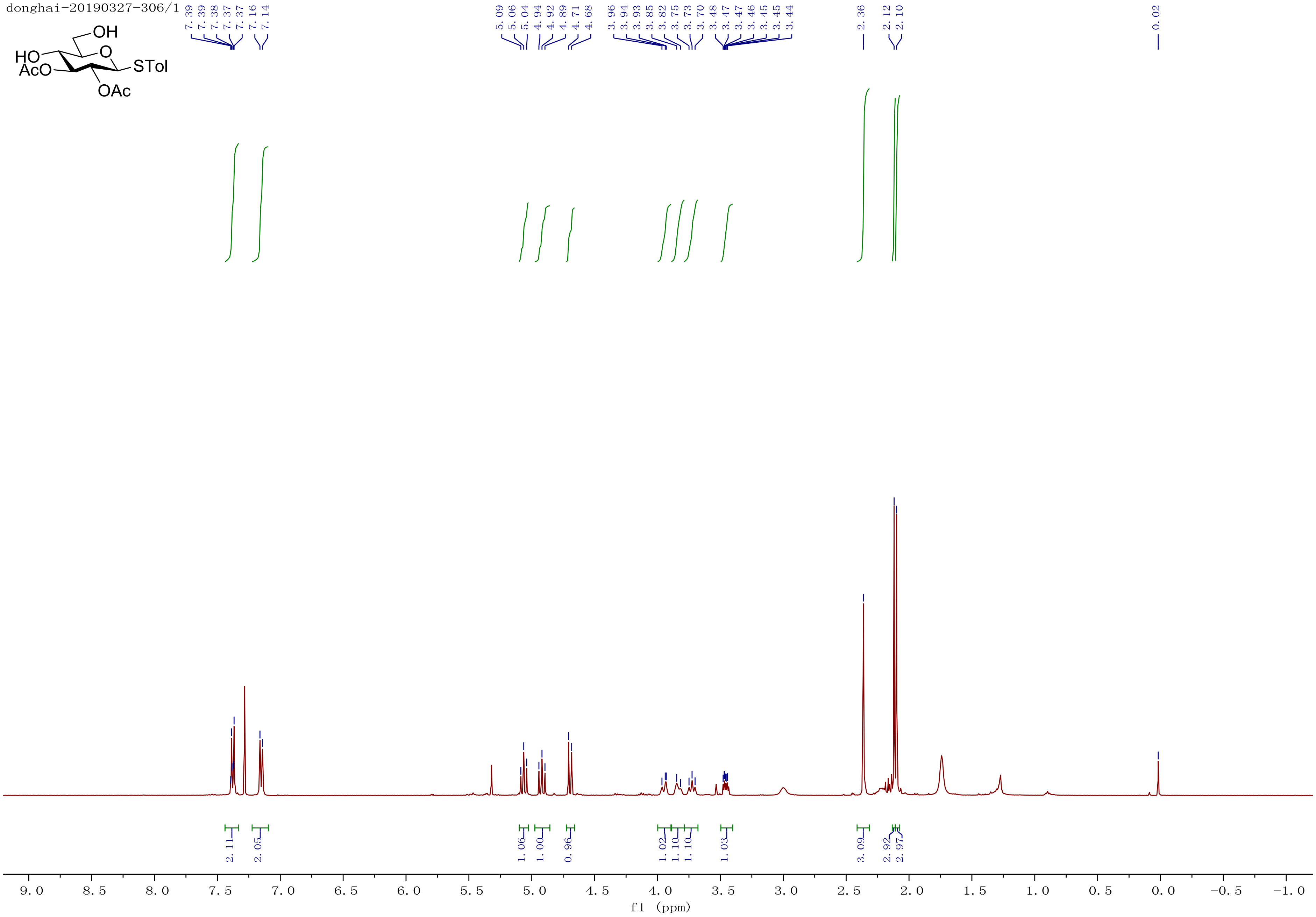

Methyl 2,3-di-*O*-benzyl-4,6-*O*-benzylidene-*α*-D-galactopyranoside 15

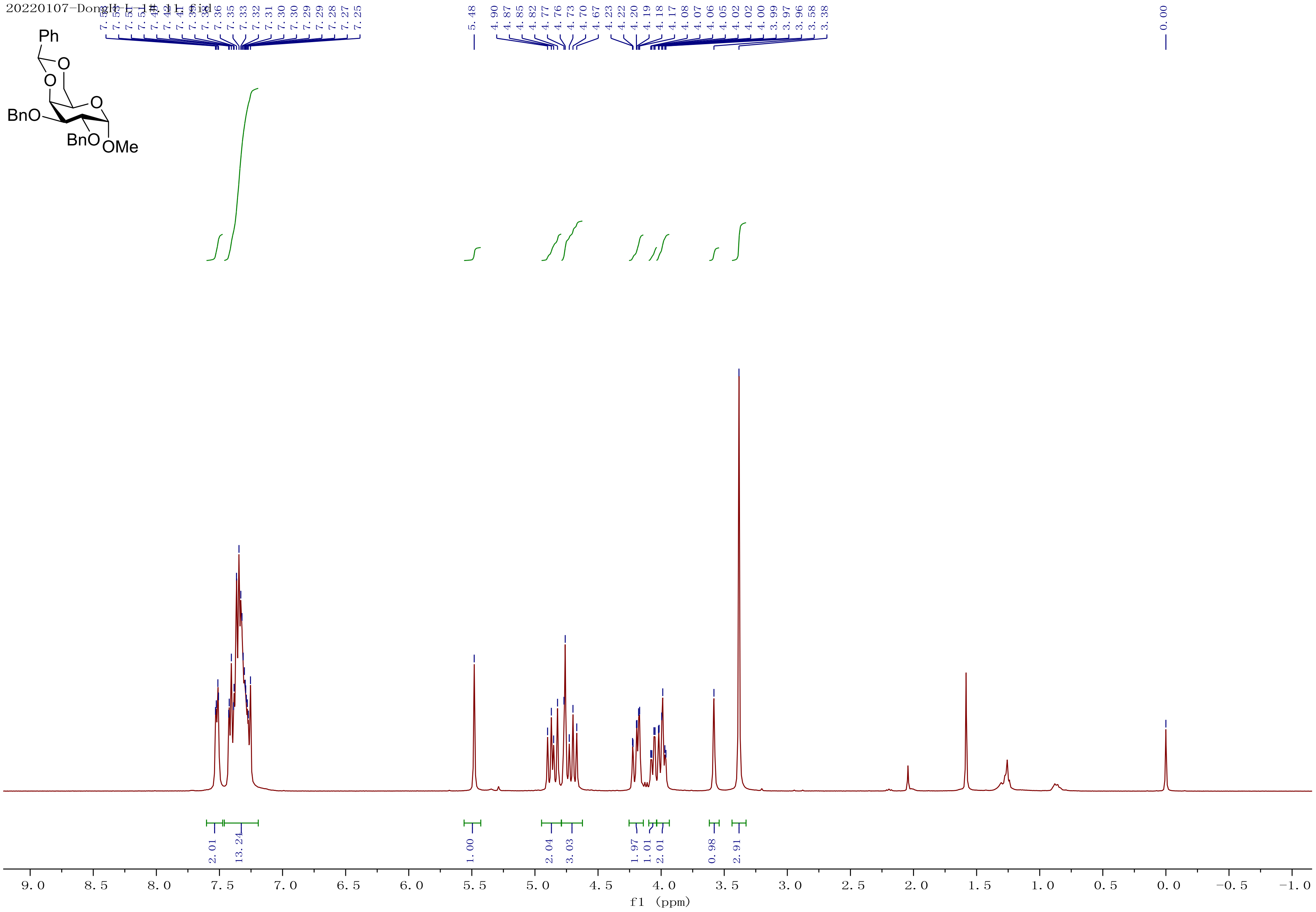

Methyl 2,3-di-*O*-benzyl-*α*-D-galactopyranoside 16

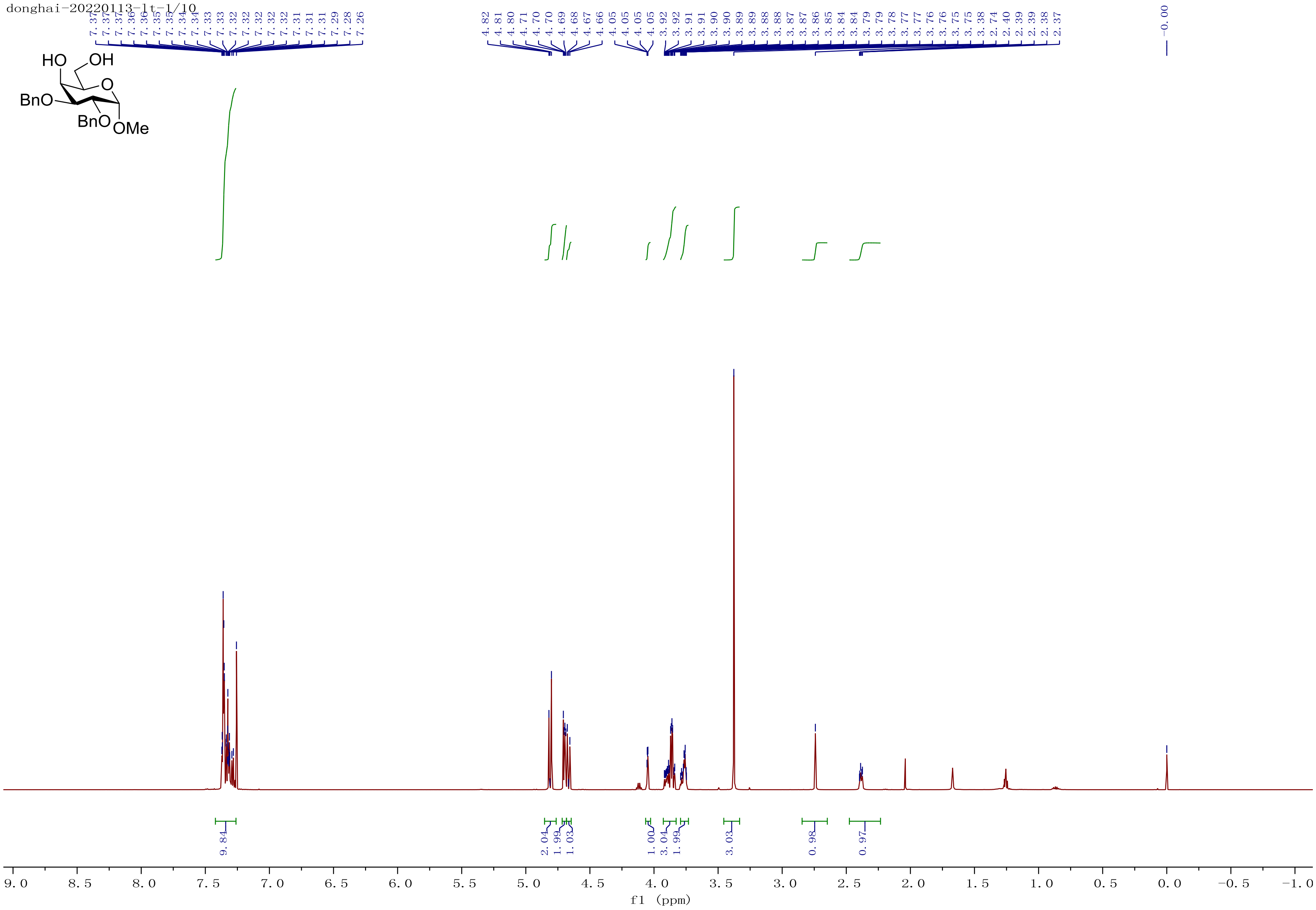

Methyl 2,3-di-*O*-benzyl-4,6-*O*-benzylidene-*β*-D-galactopyranoside 17

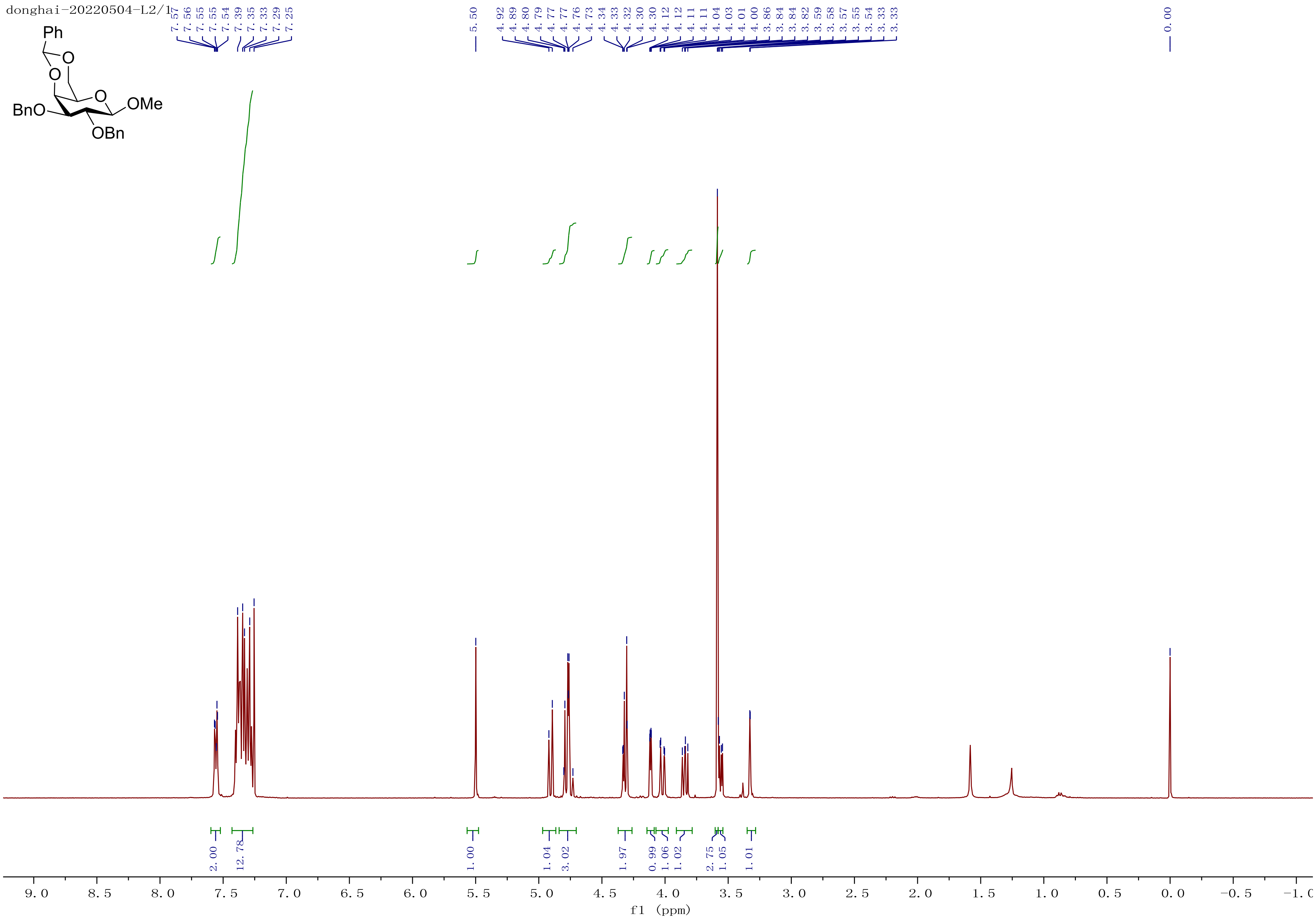

Methyl 2,3-di-*O*-benzyl-*β*-D-galactopyranoside 18

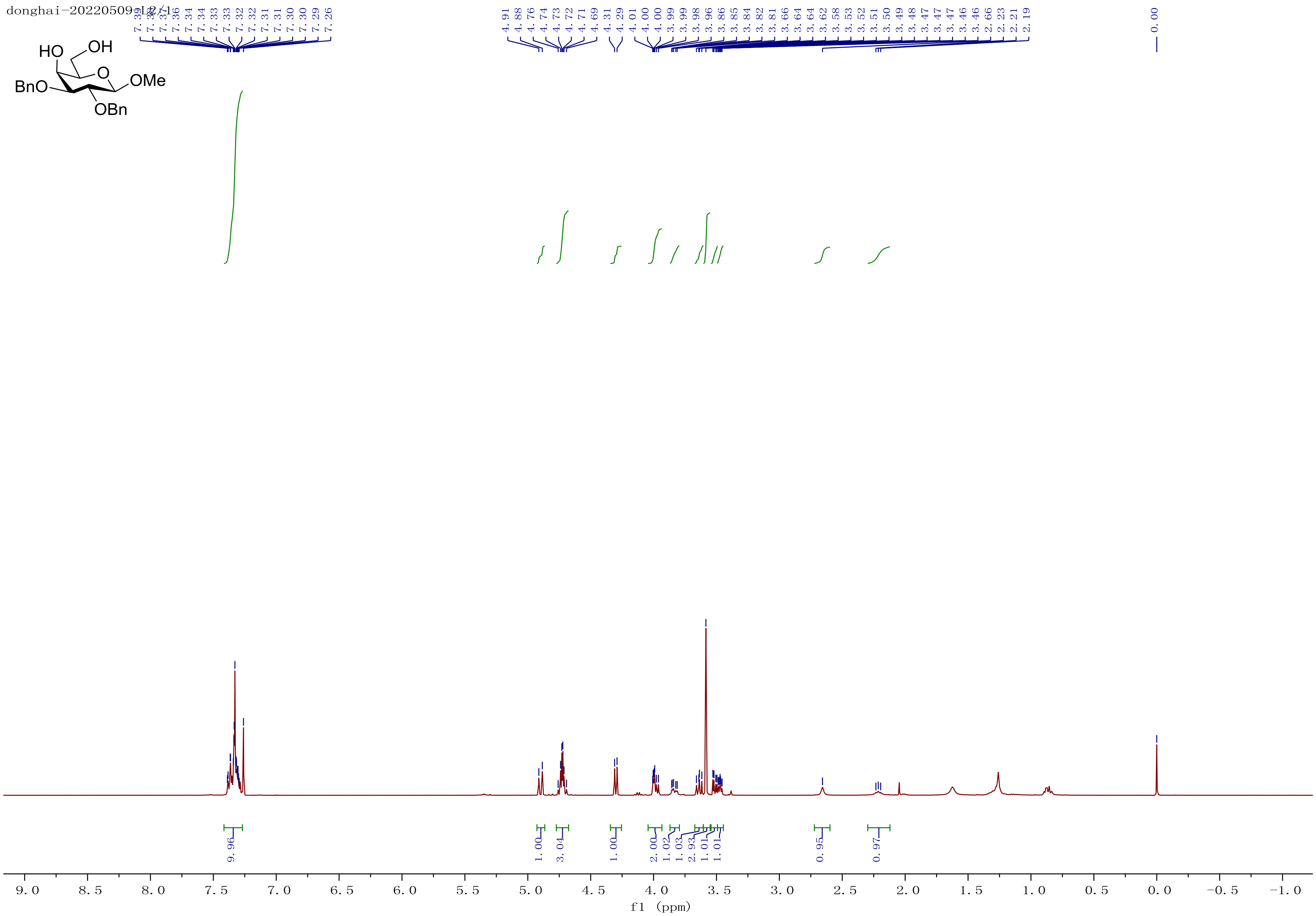

Methyl 2,3-di-*O*-acetyl-4,6-*O*-benzylidene-*α*-D-mannopyranoside 19

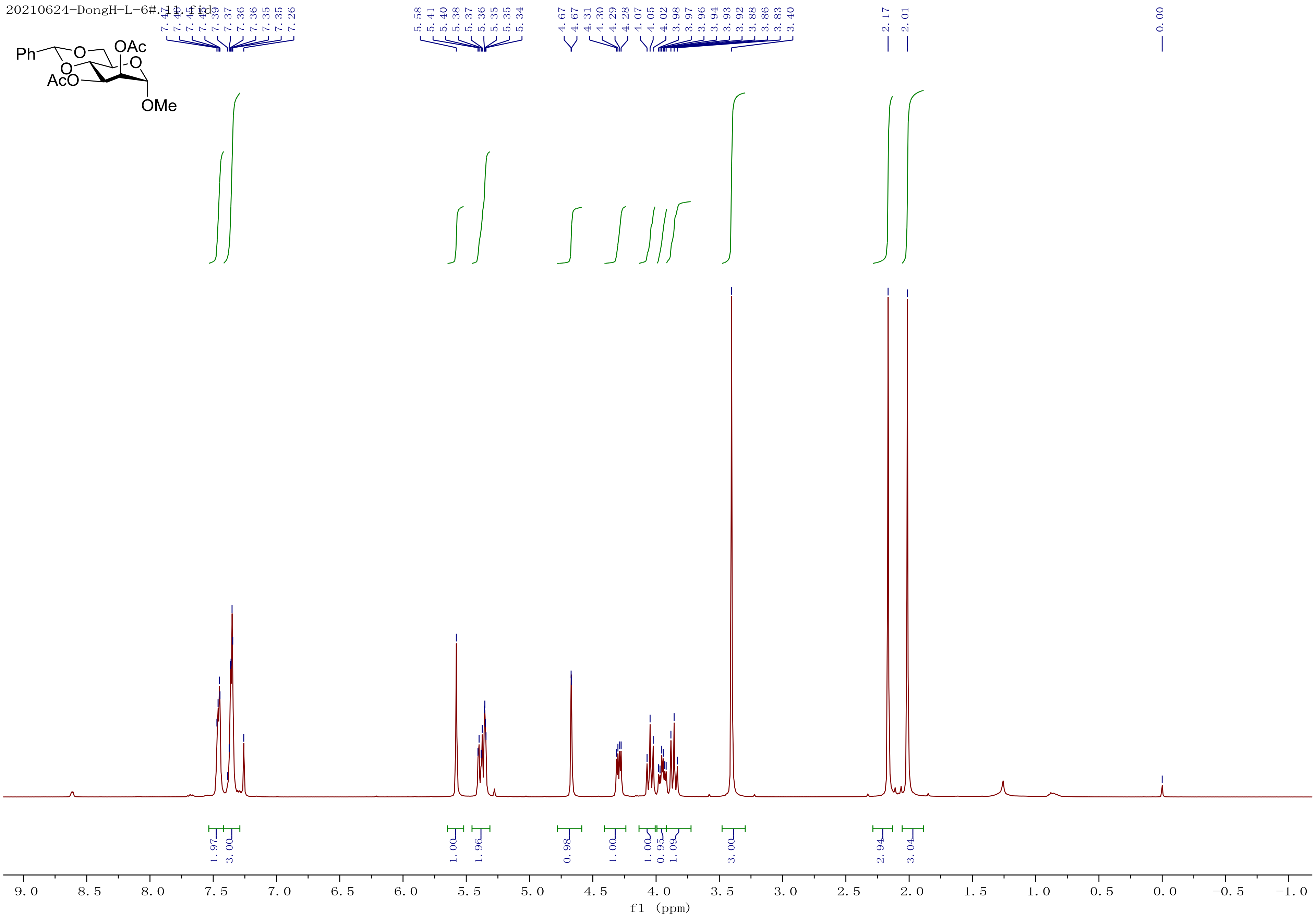

Methyl 2,3-di-*O*-acetyl-*α*-D-mannopyranoside 20

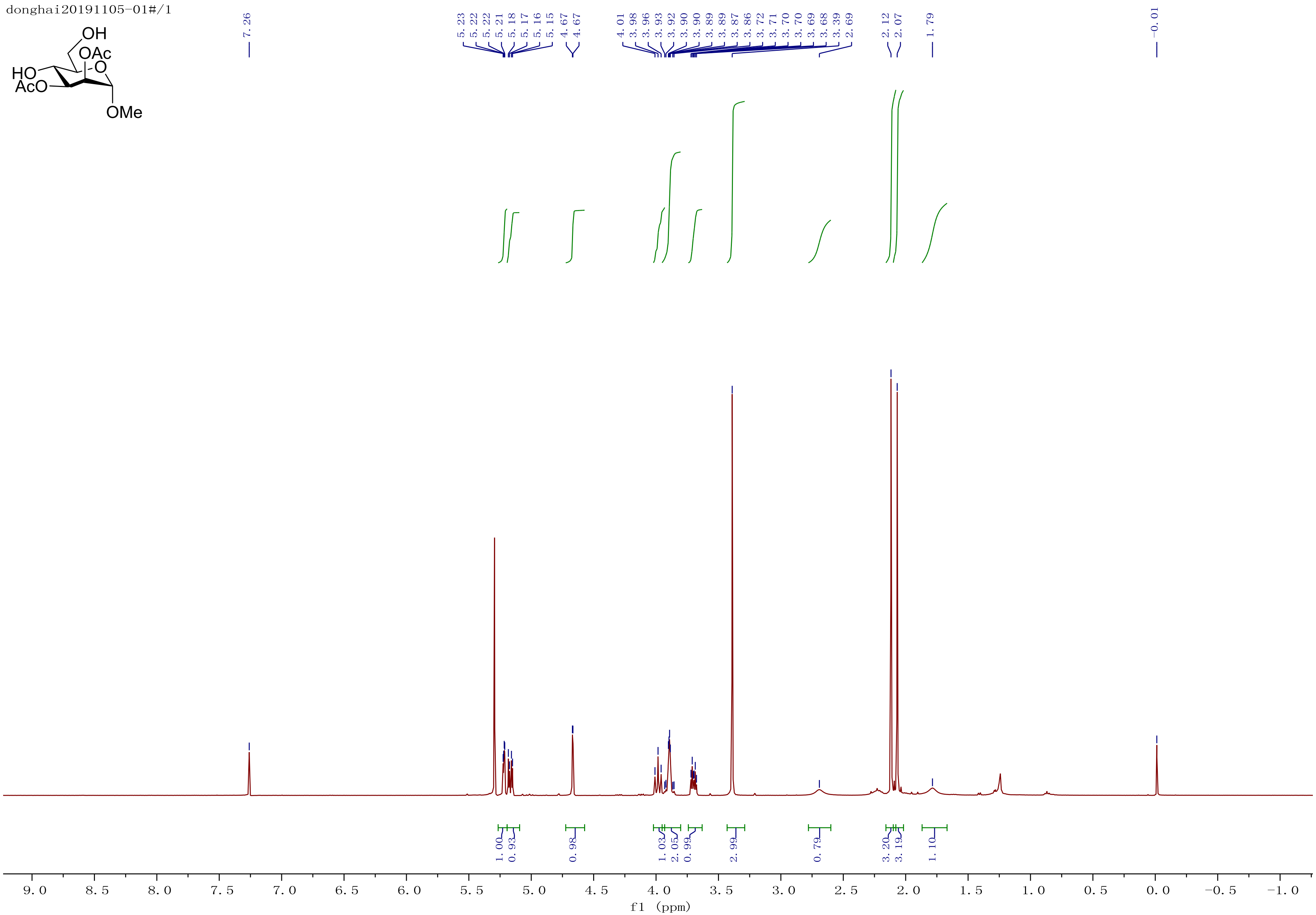

Methyl 2,6-di-*O*-benzyl 3,4-*O*-isopropylidene-*α*-D-galactopyranoside 21

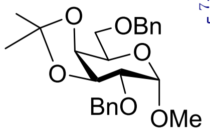

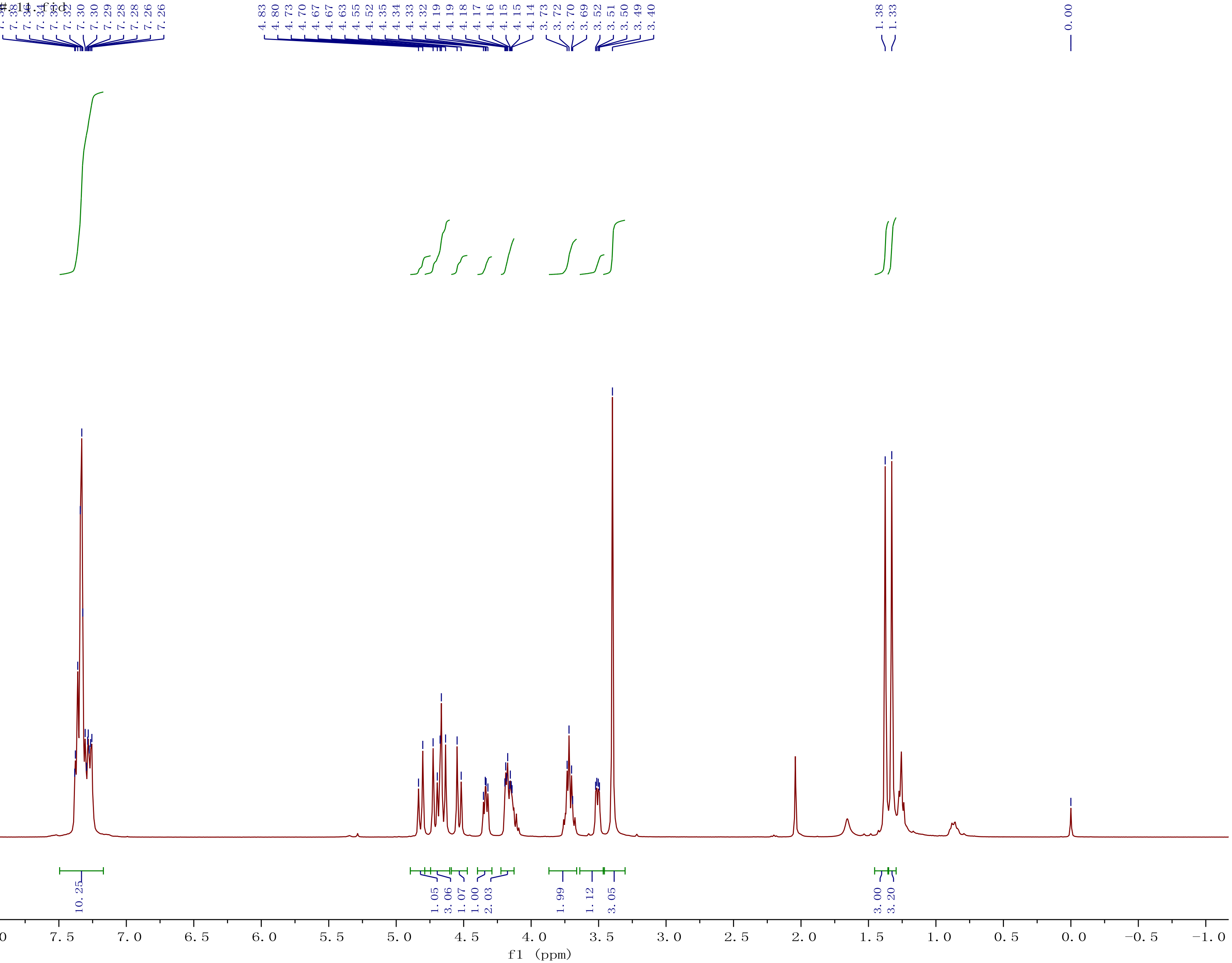

Methyl 2,6-di-*O*-benzyl-*α*-D-galactopyranoside 22

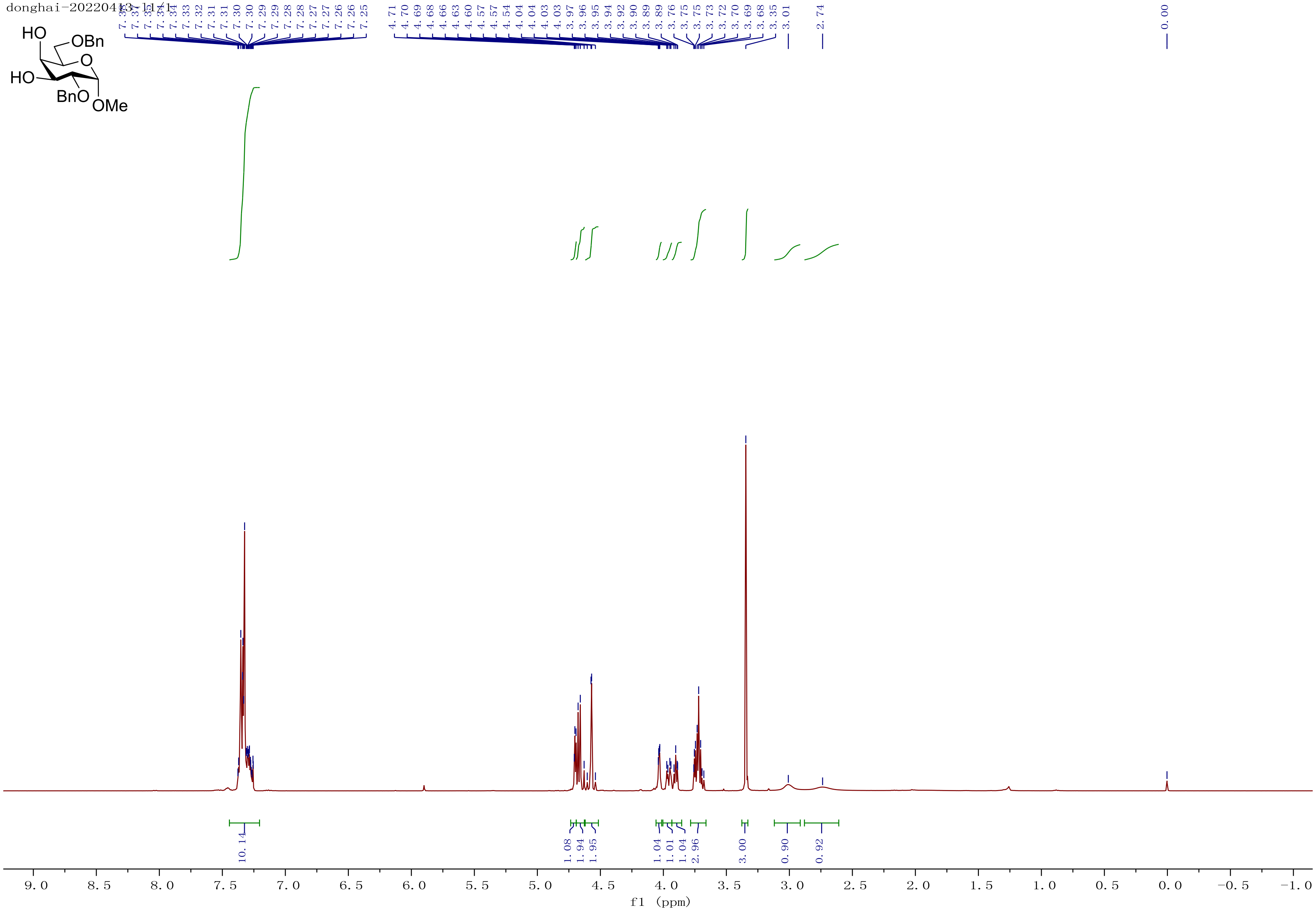

1,2:5,6-Di-*O*-isopropylidene-3-*O*-benzoyl-*α*-D-glucofuranose 23

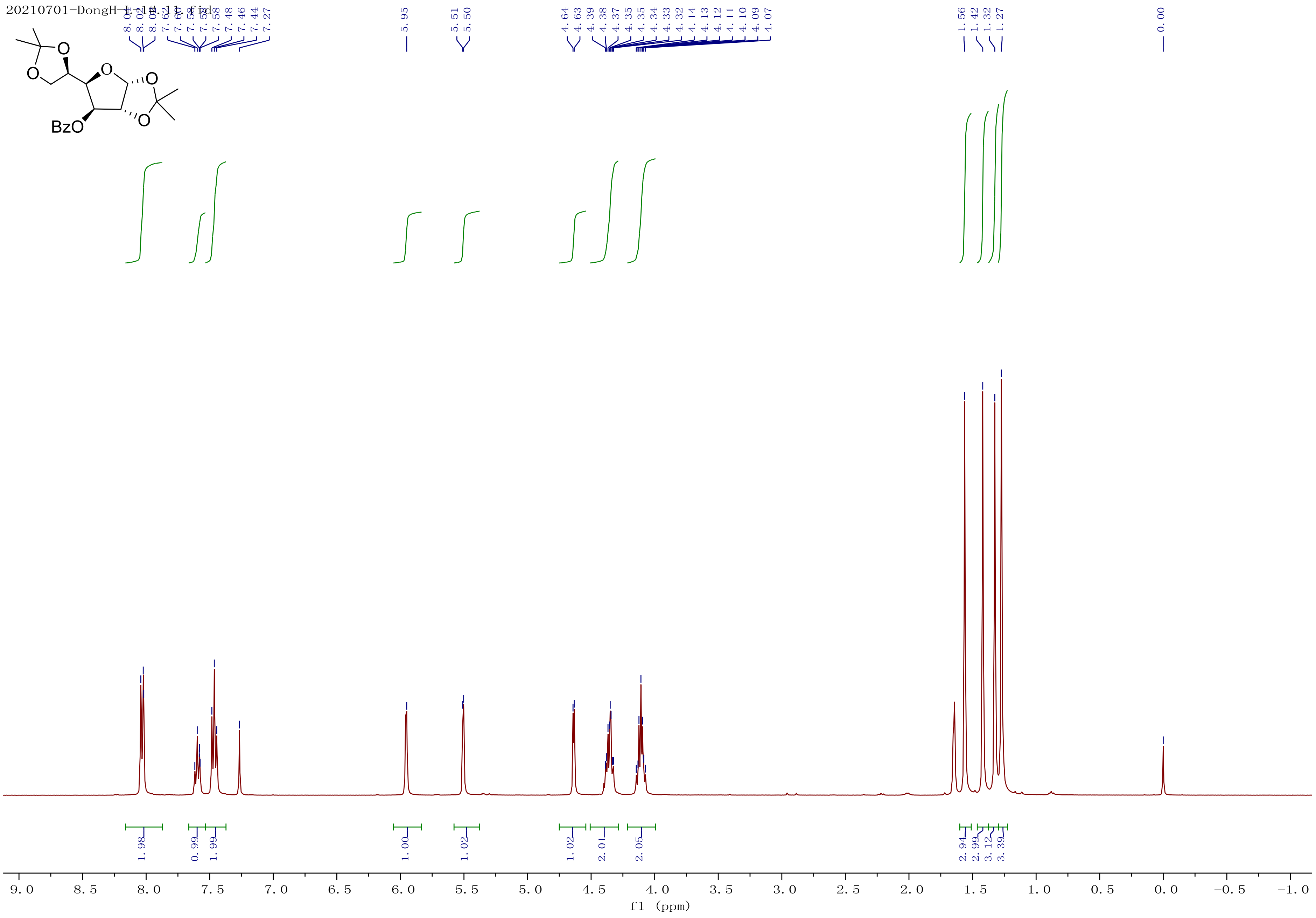

3-*O*-Benzoyl-1,2-*O*-isopropylidene-*α*-D-glucofuranose 24

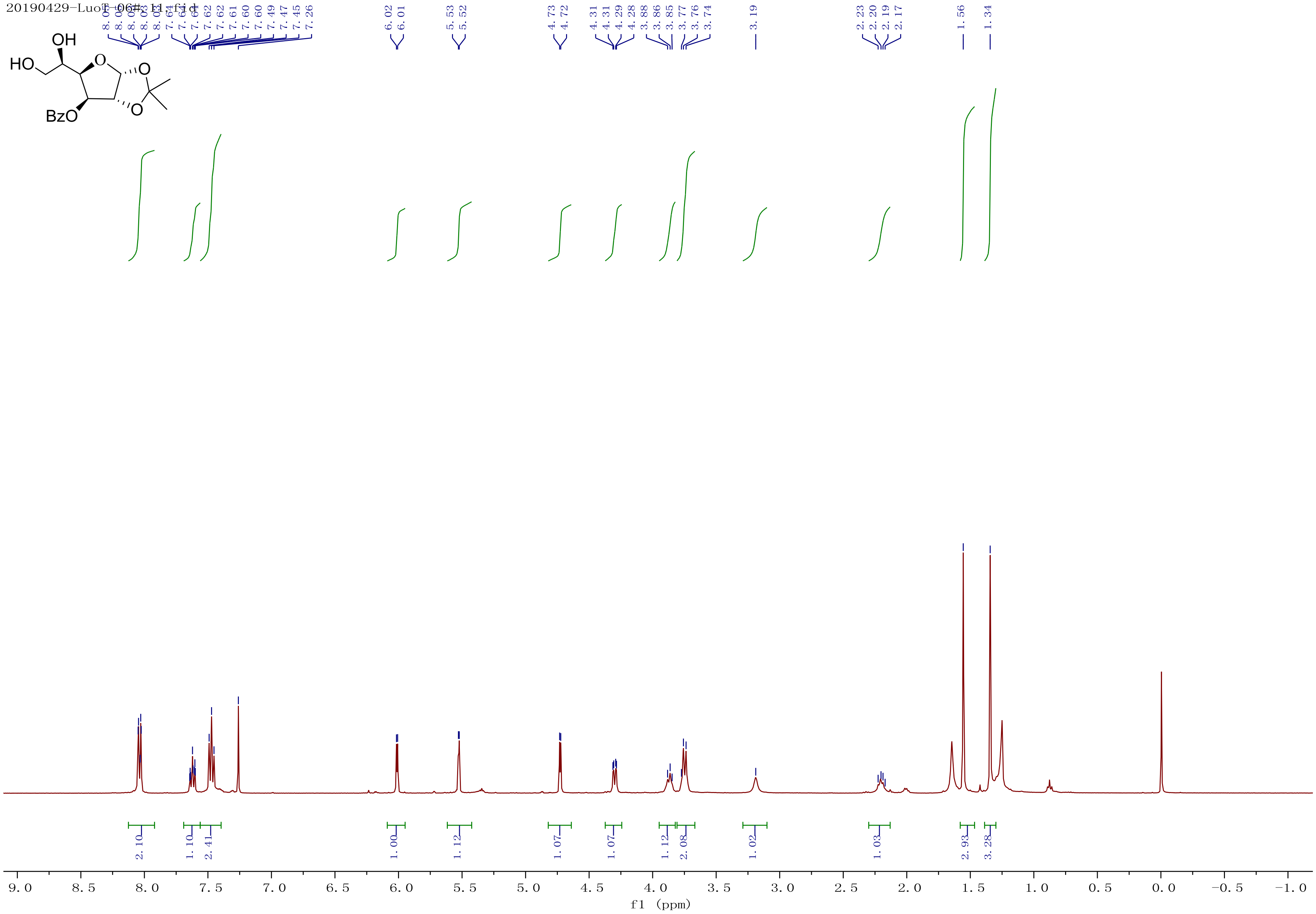

1,2:5,6-Di-*O*-isopropylidene-3-*O*-benzyl-*α*-D-glucofuranose 25

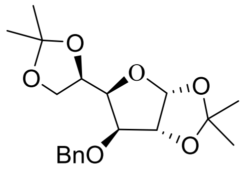

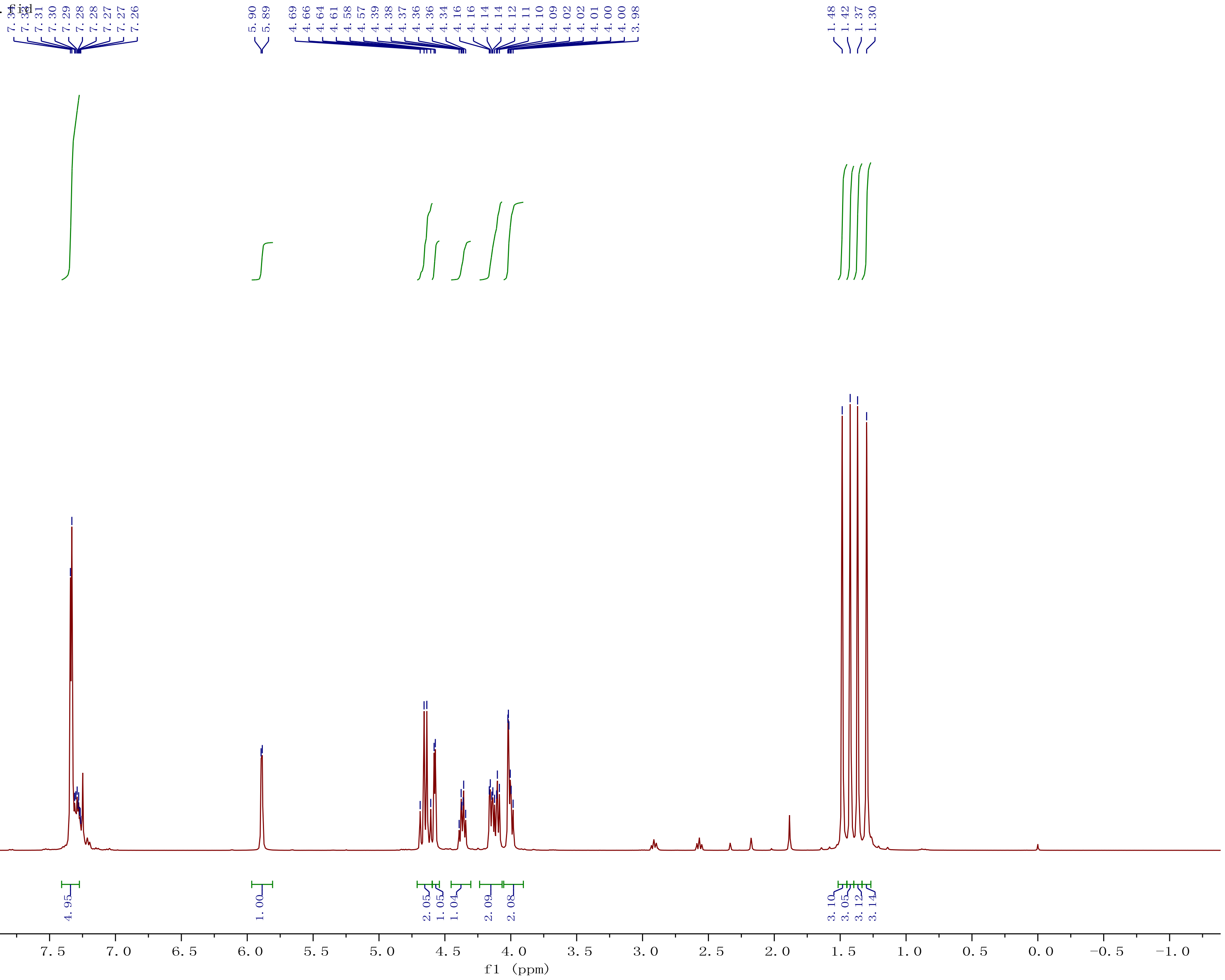

3-*O*-Benzyl-1,2-*O*-isopropylidene-*α*-D-glucofuranose 26

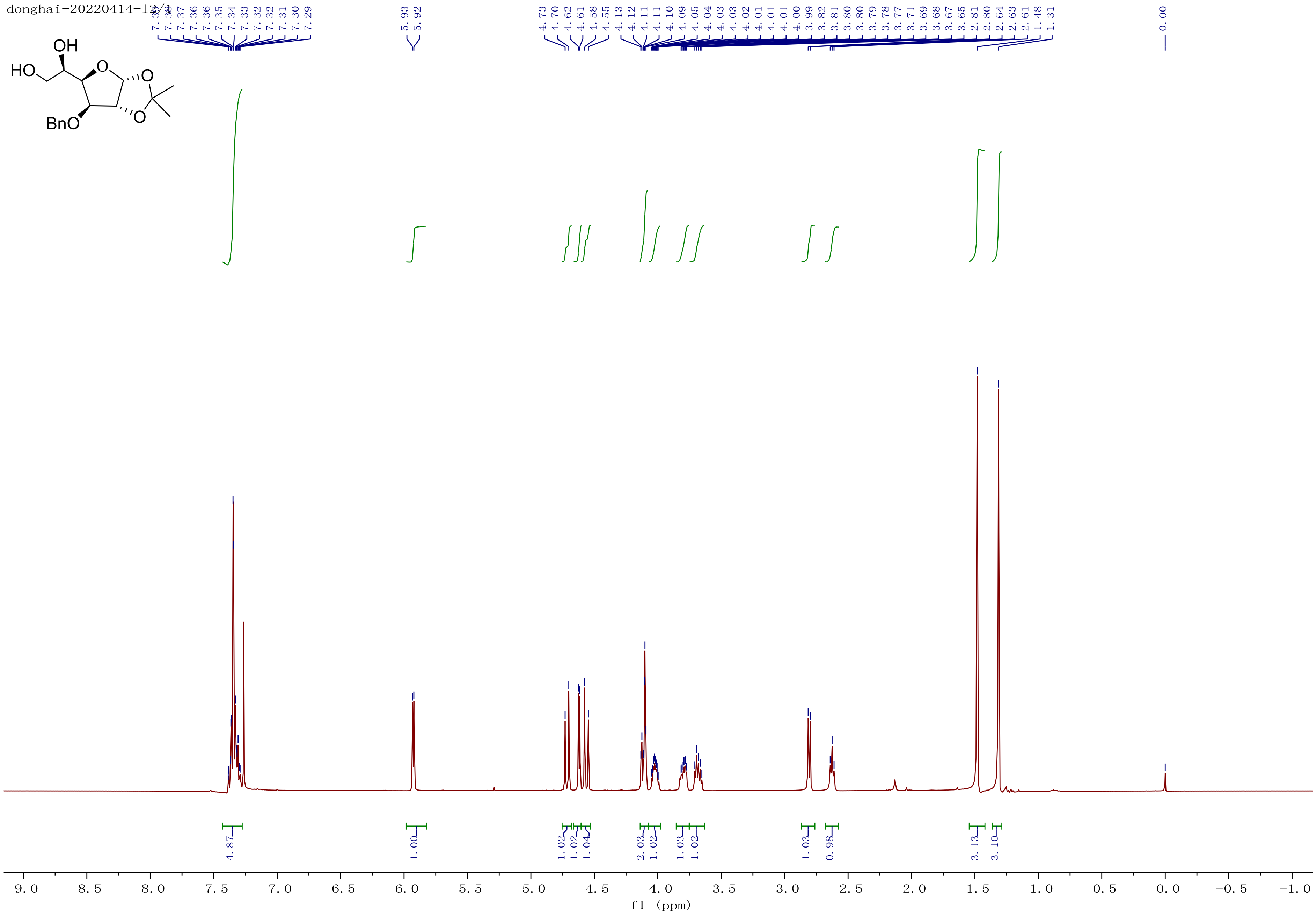

Methyl 2,4,6-tri-*O*-acetyl-3-*O*-(*p*-methoxybenzyl)-*β*-D-galactopyranoside 27

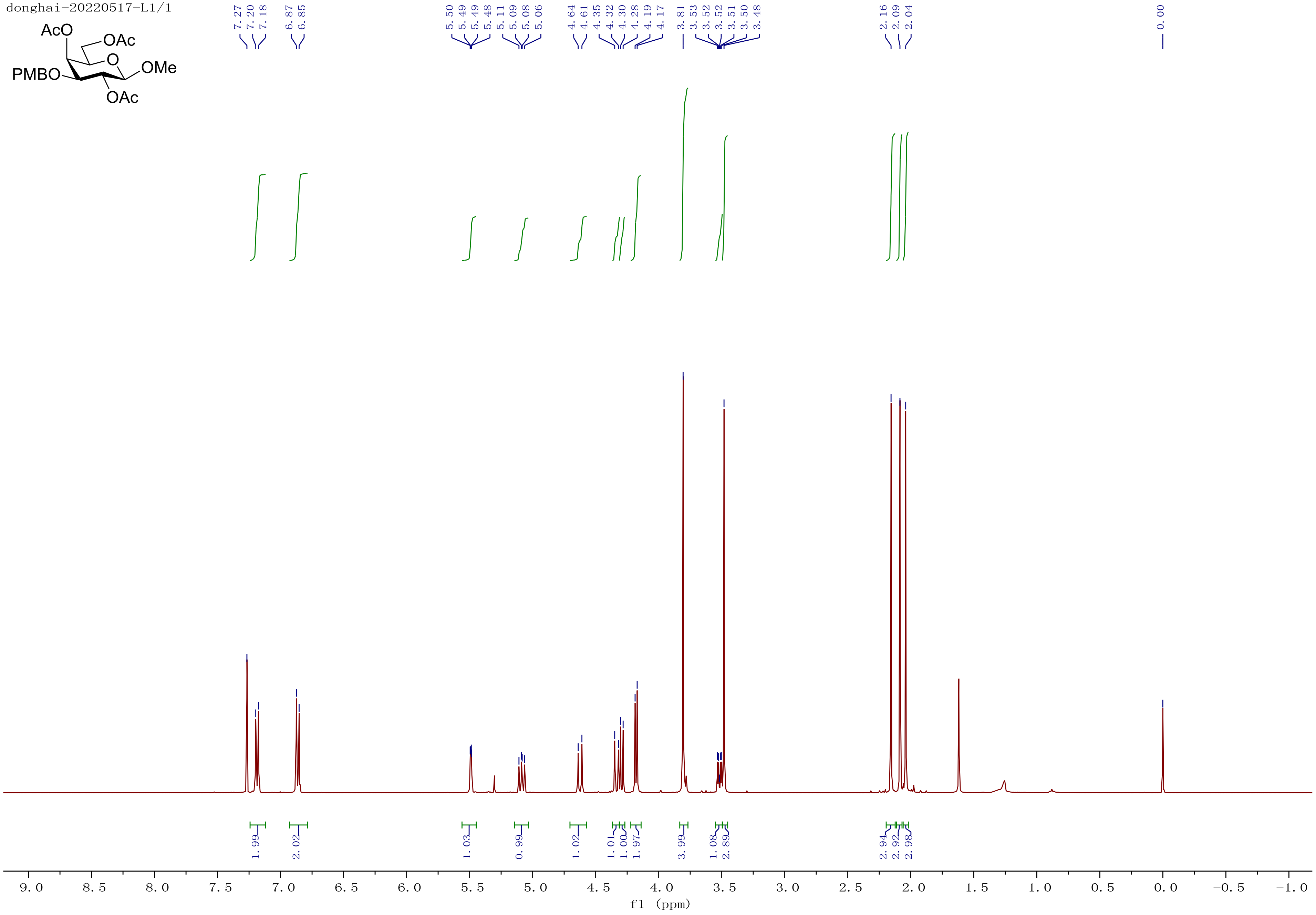

Methyl 2,4,6-tri-*O*-acetyl-3-*O*-(*p*-methoxybenzyl)-*β*-D-galactopyranoside 27

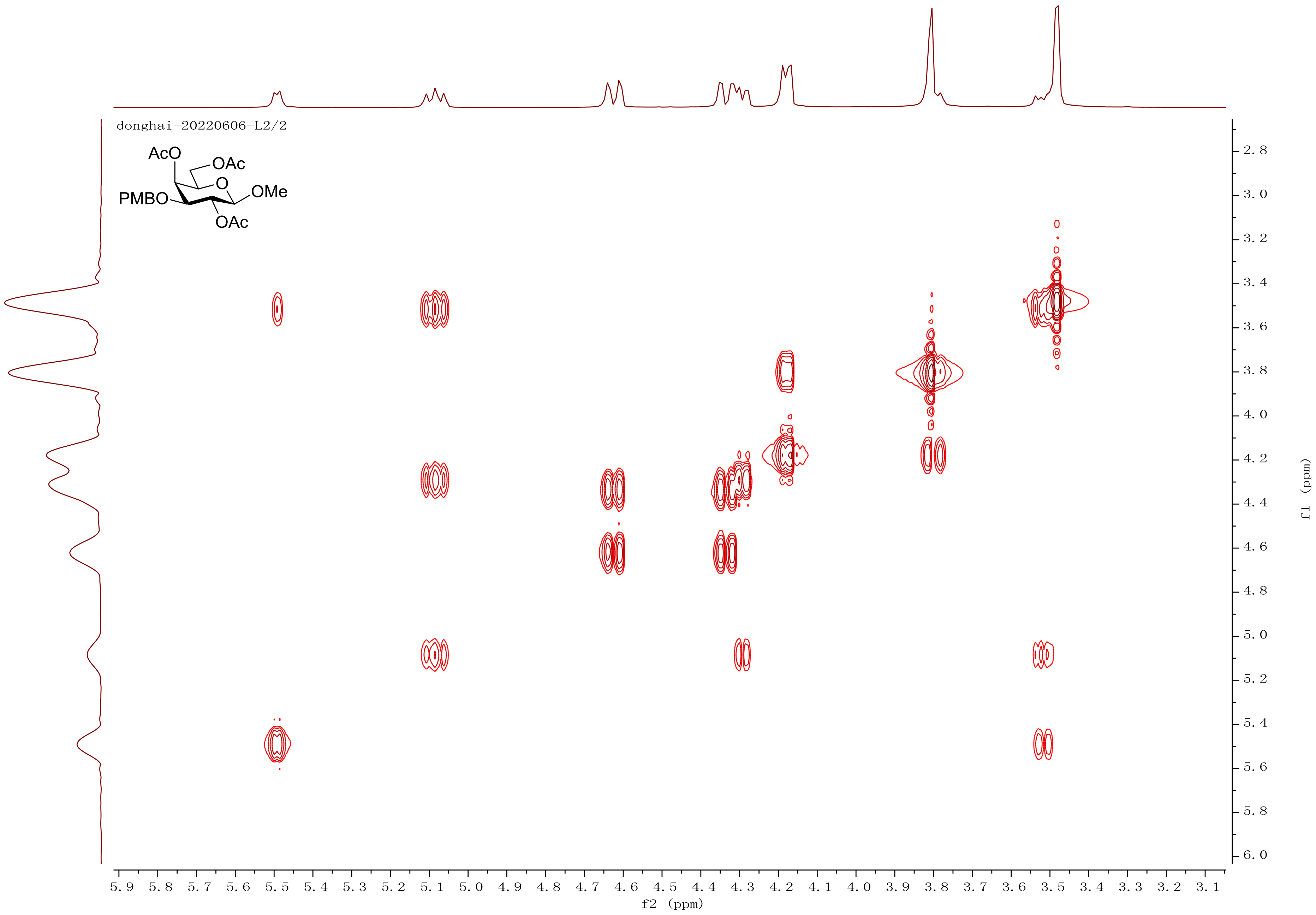

Methyl 2,4,6-tri-*O*-acetyl-3-*O*-(*p*-methoxybenzyl)-*β*-D-galactopyranoside 27

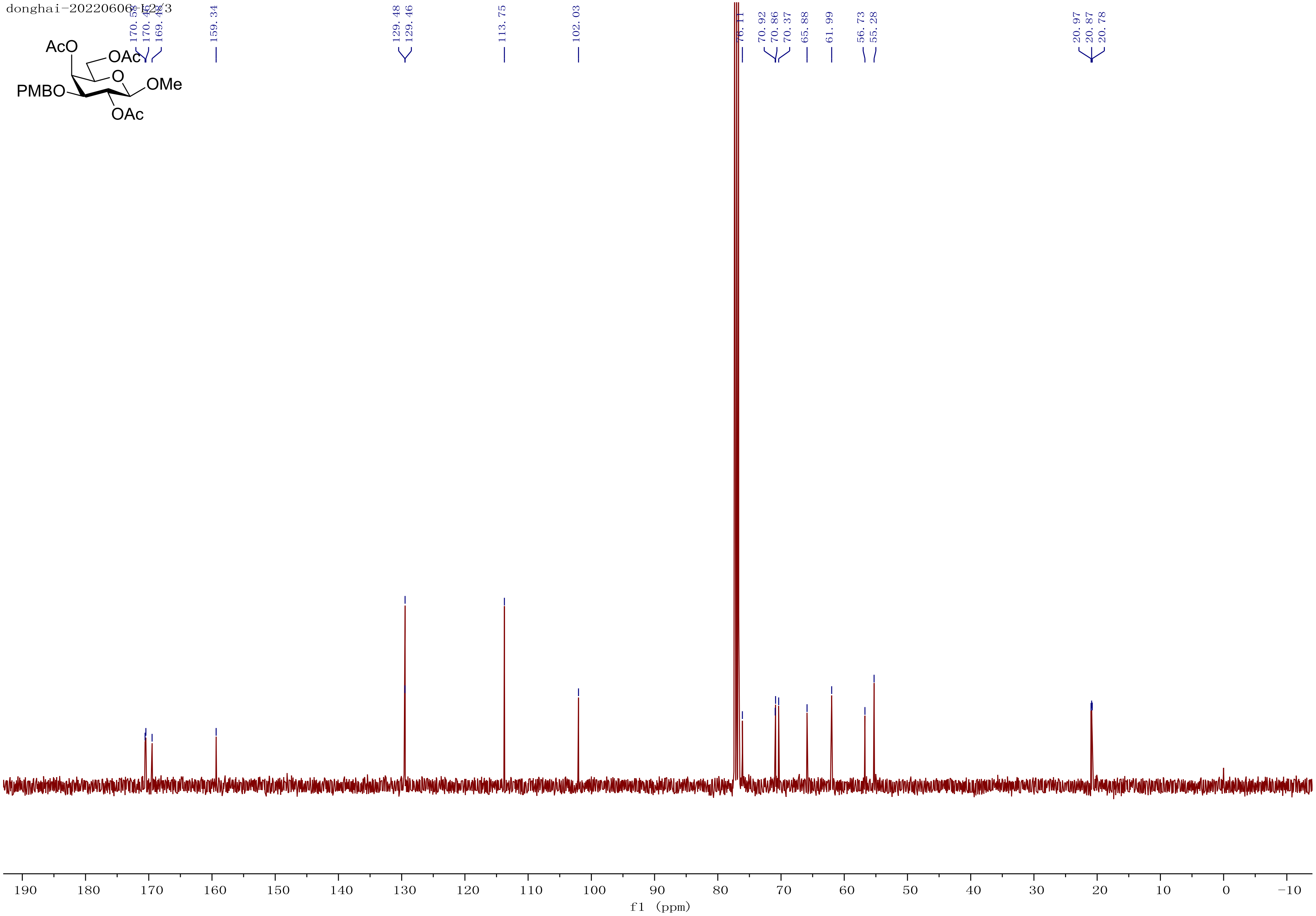

Methyl 2,4,6-tri-*O*-acetyl-*β*-D-galactopyranoside 28

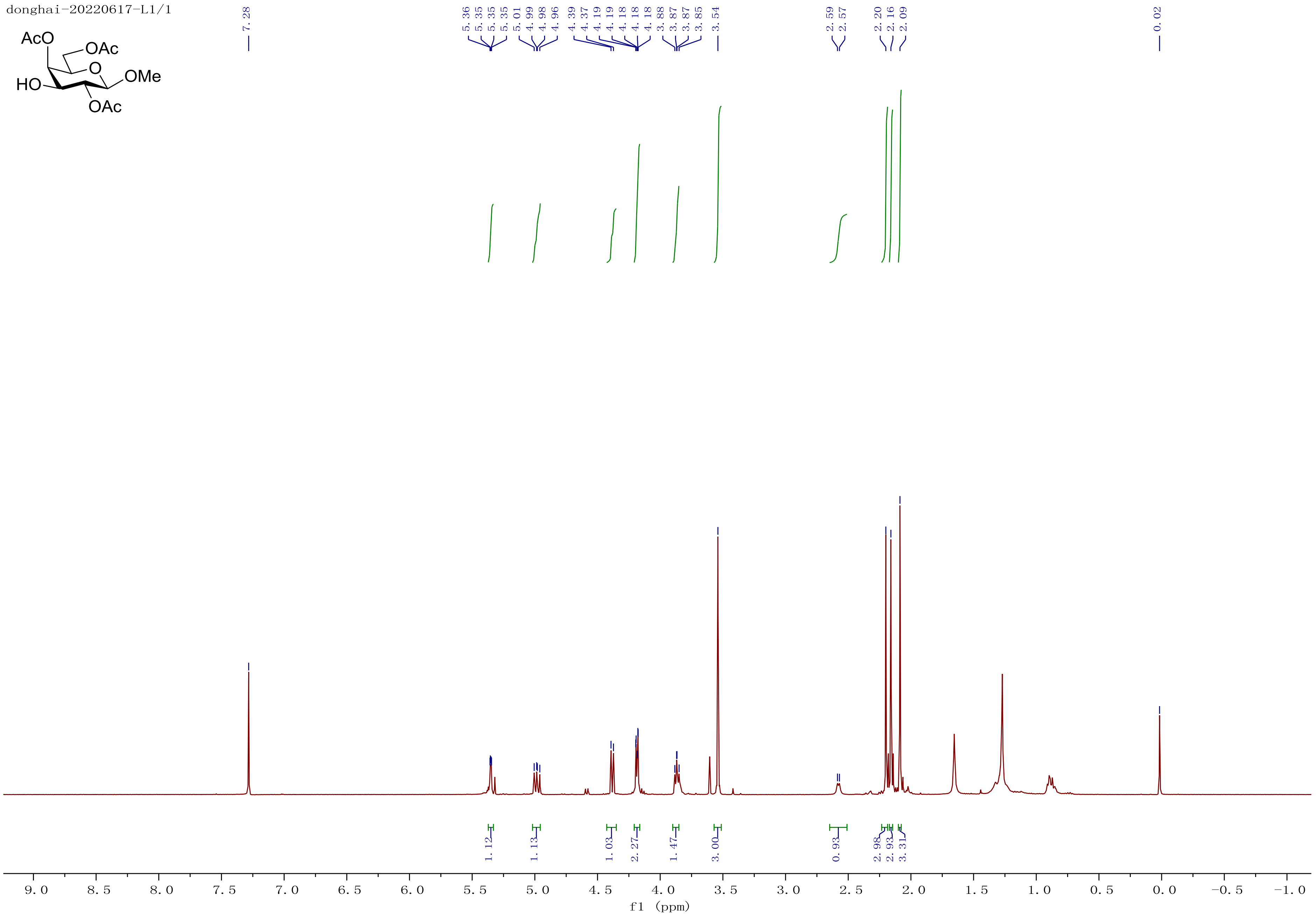

Methyl 2,4,6-tri-*O*-acetyl-3-*O*-(*p*-methoxybenzyl)-*α*-D-mannopyranoside 29

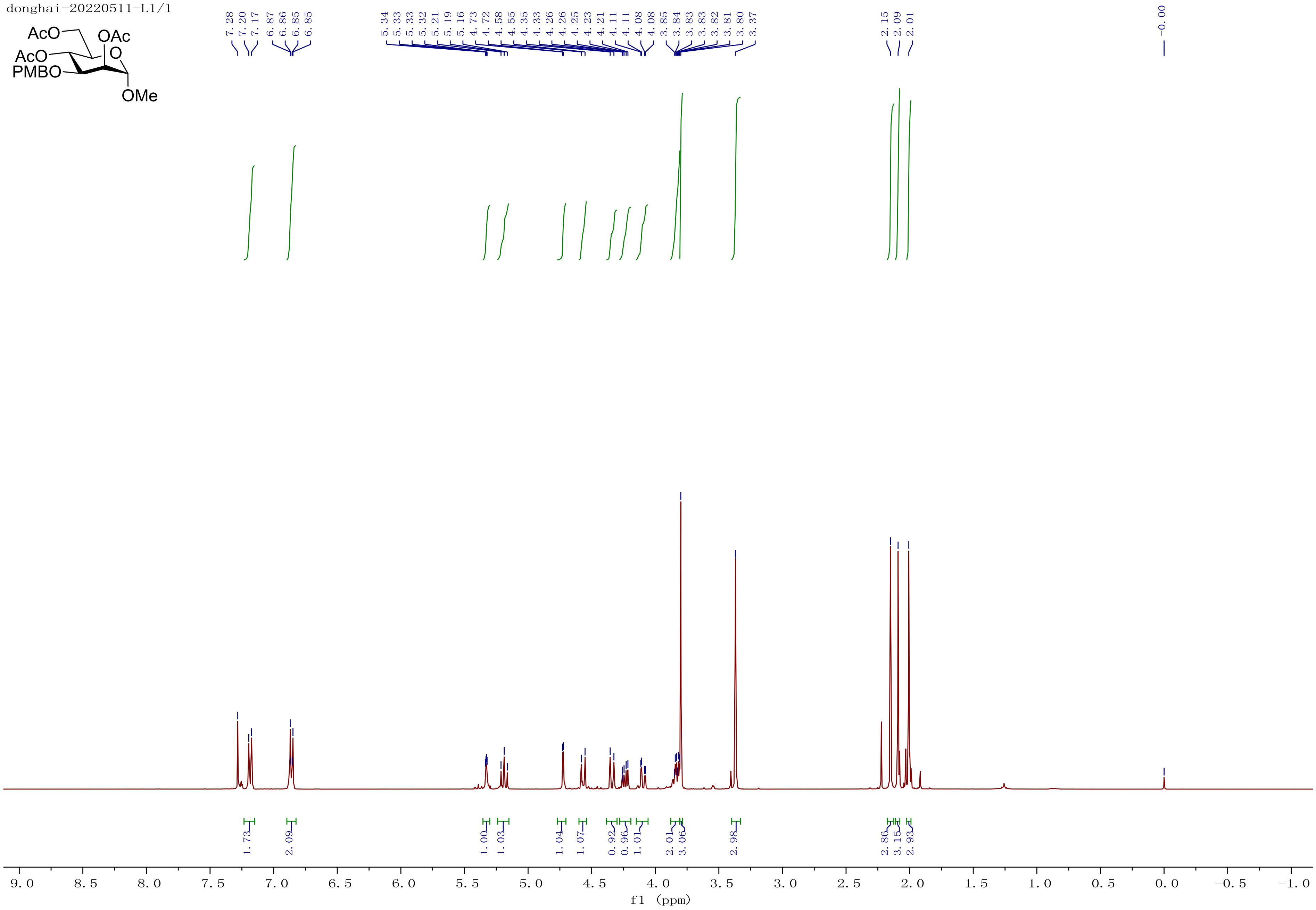

Methyl 2,4,6-tri-*O*-acetyl-3-*O*-(*p*-methoxybenzyl)-*α*-D-mannopyranoside 29

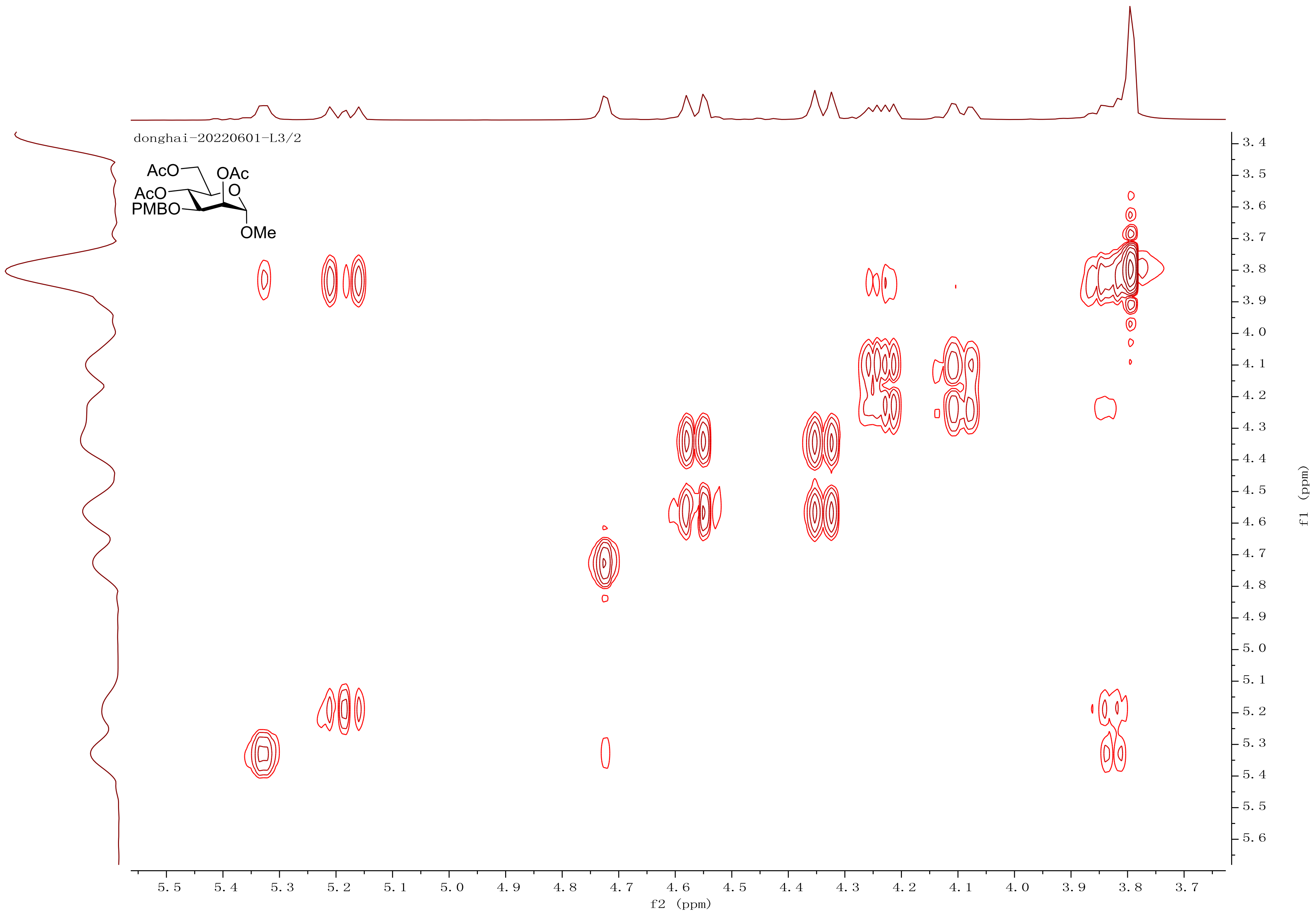

Methyl 2,4,6-tri-*O*-acetyl-3-*O*-(*p*-methoxybenzyl)-*α*-D-mannopyranoside 29

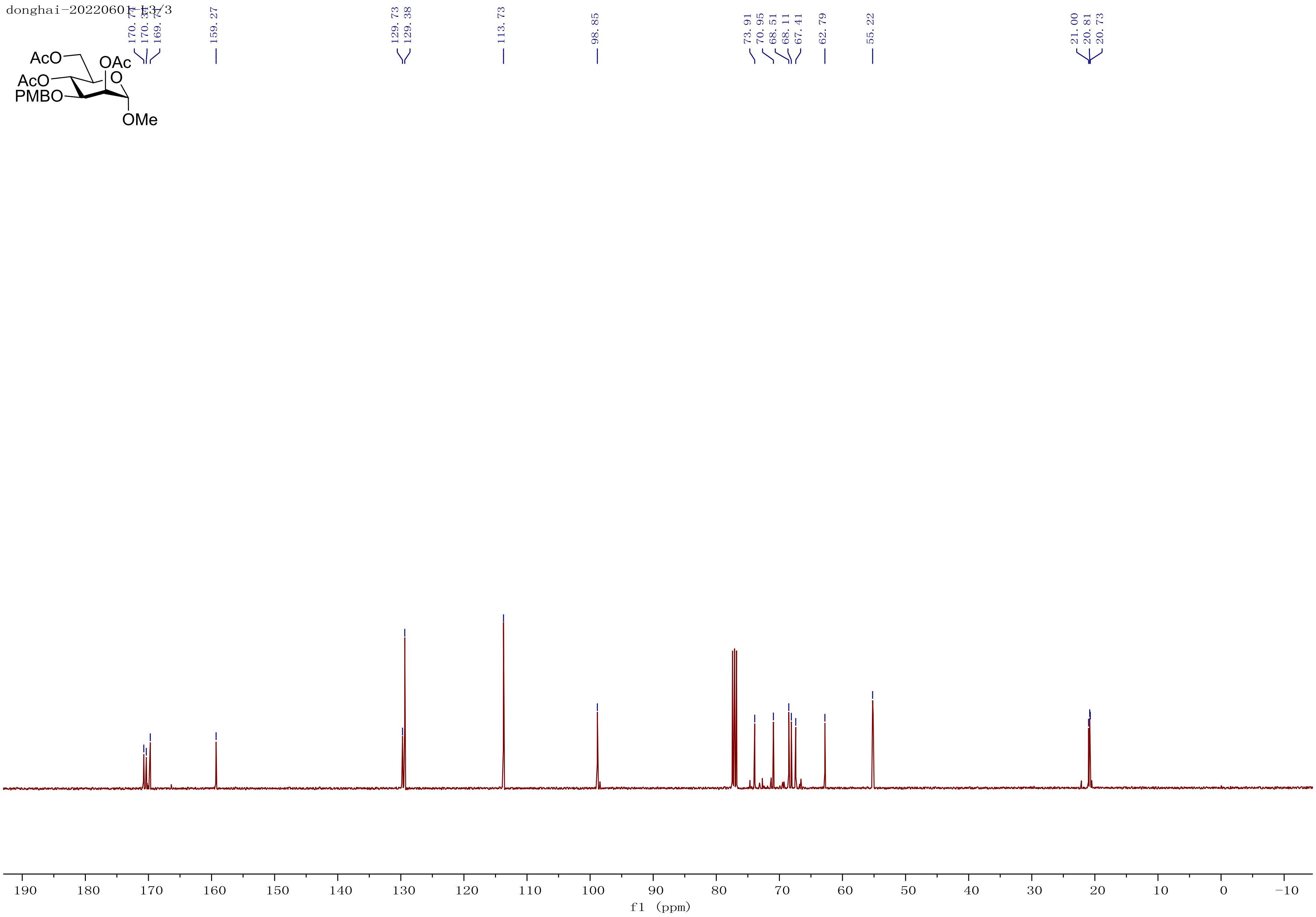

Methyl 2,4,6-tri-*O*-acetyl-*α*-D-mannopyranoside 30

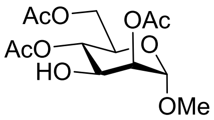

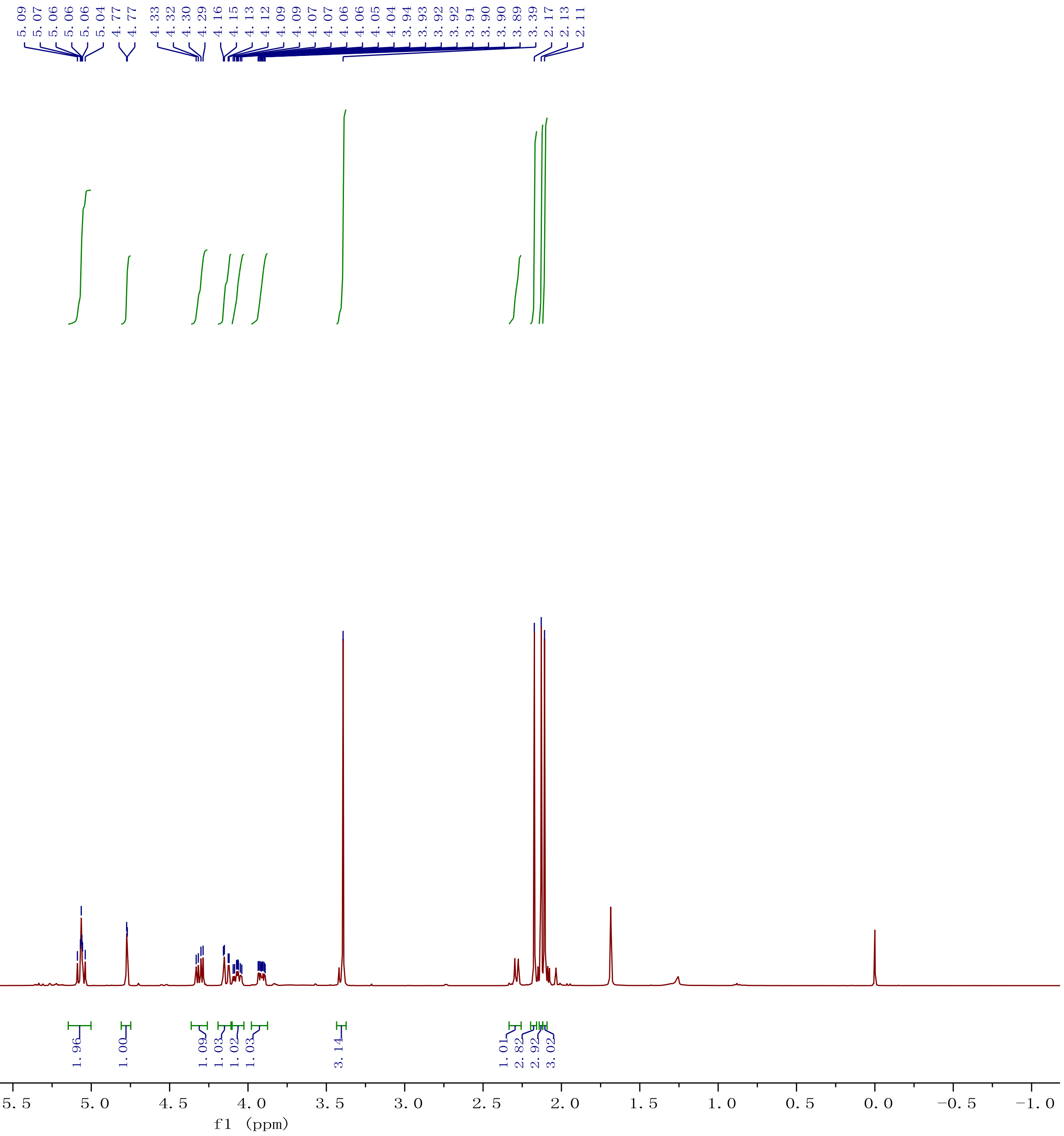

Methyl 2-*O*-*p*-methoxybenzyl-3-*O*-benzyl-4,6-*O*-benzylidene-*α*-D-glucopyranoside 31

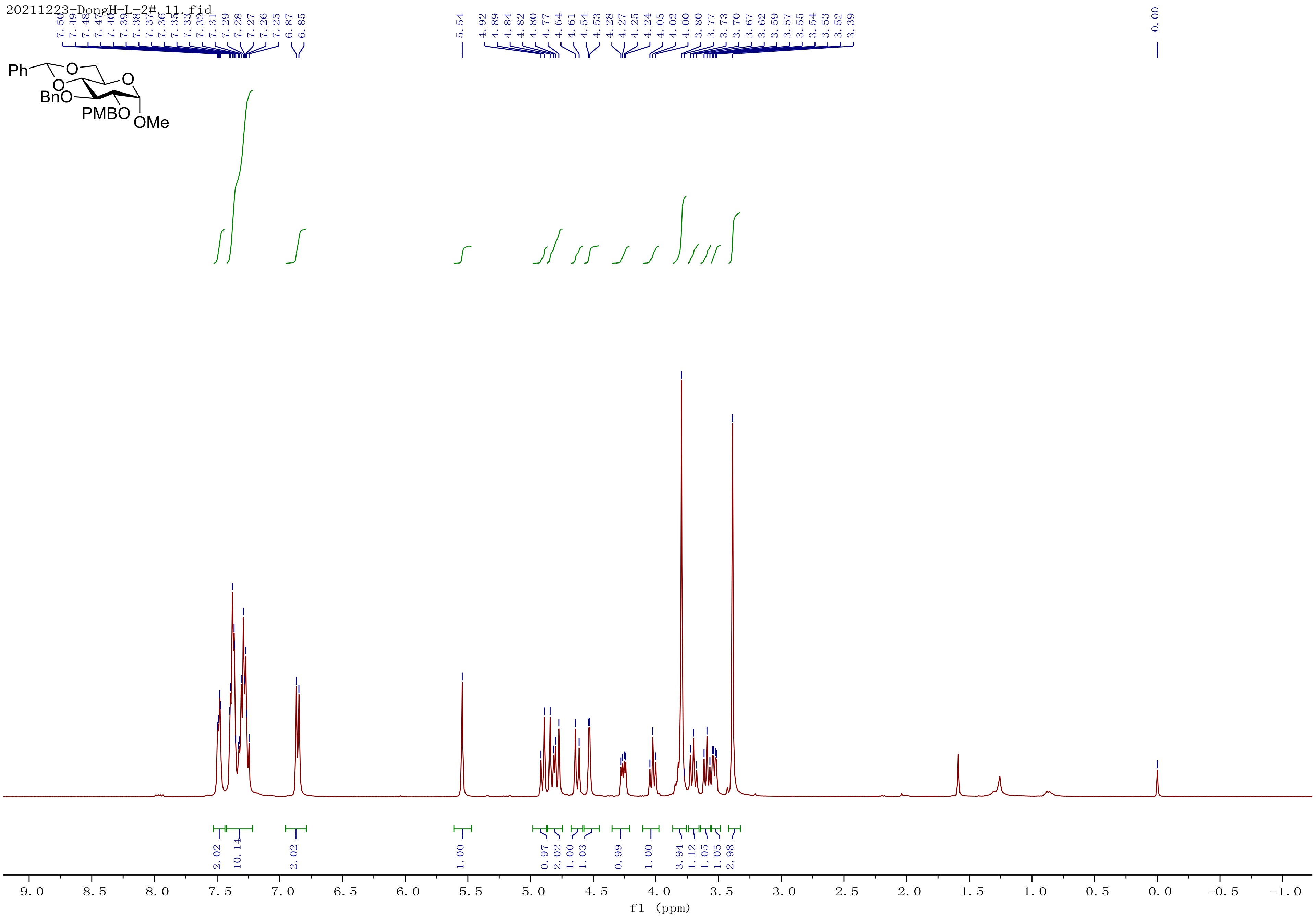

Methyl 2-*O*-*p*-methoxybenzyl-3-*O*-benzyl-4,6-*O*-benzylidene-*α*-D-glucopyranoside 31

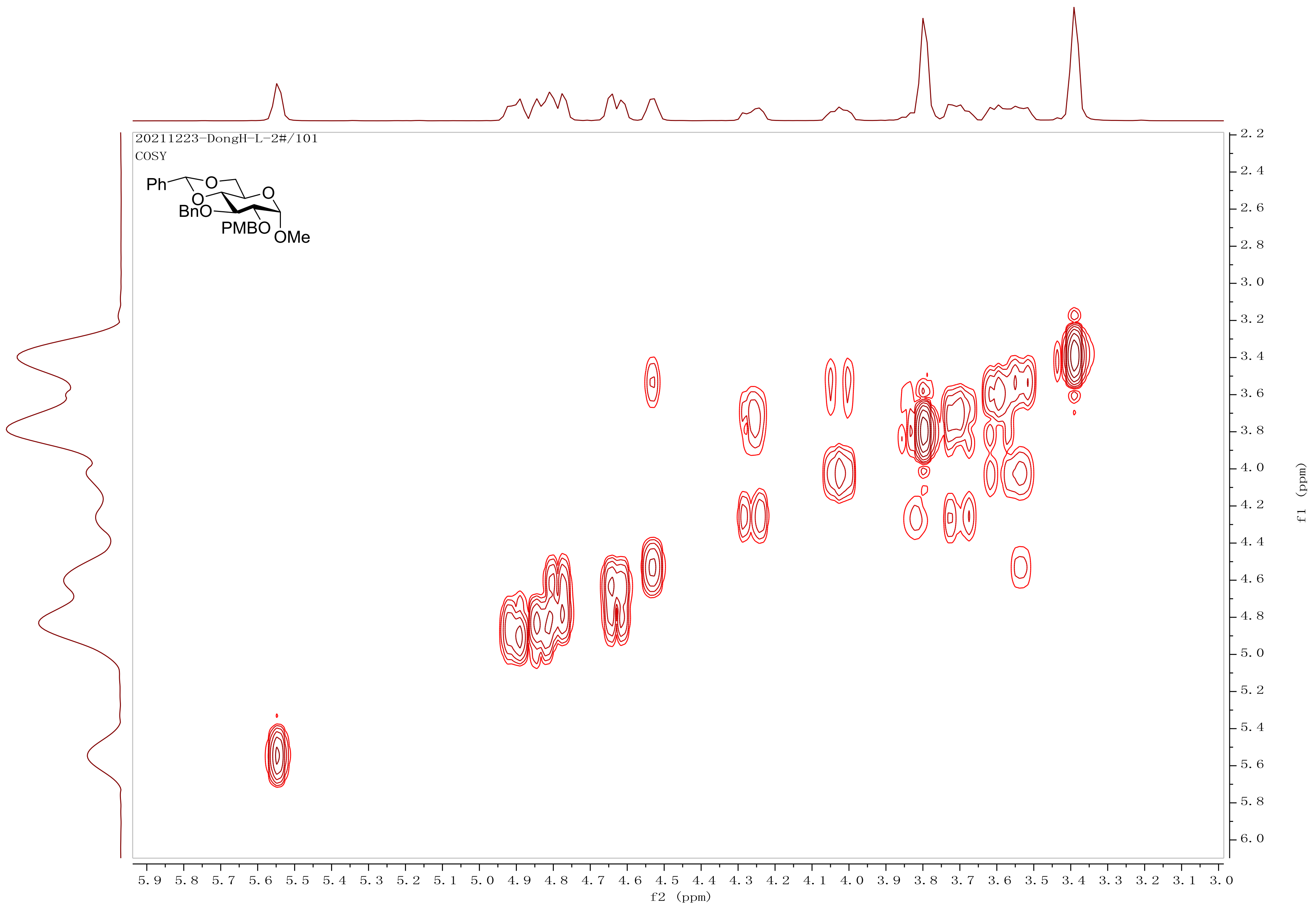

Methyl 2-*O*-*p*-methoxybenzyl-3-*O*-benzyl-4,6-*O*-benzylidene-*α*-D-glucopyranoside 31

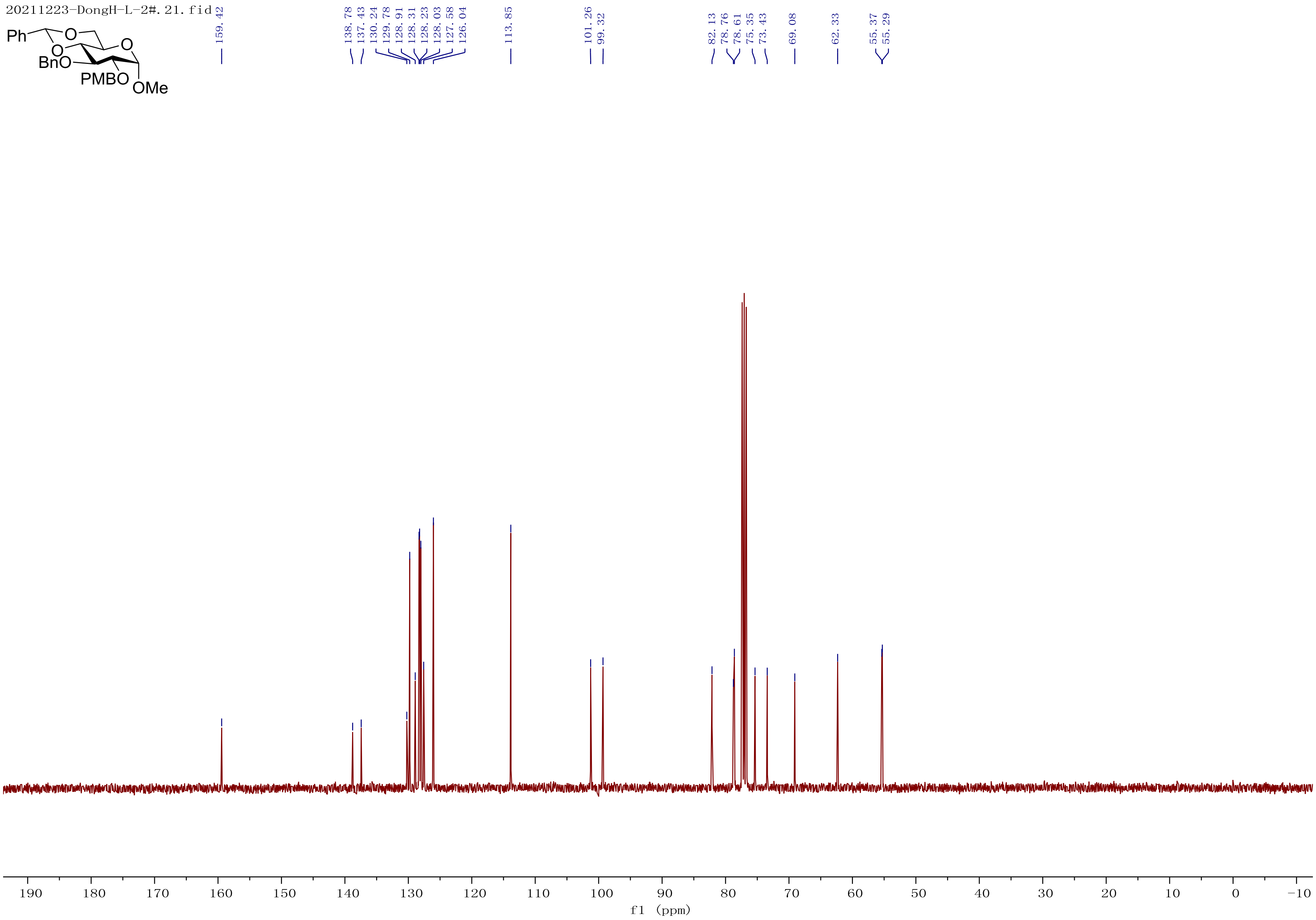

Methyl 3-*O*-benzyl-4,6-*O*-benzylidene-*α*-D-glucopyranoside 32

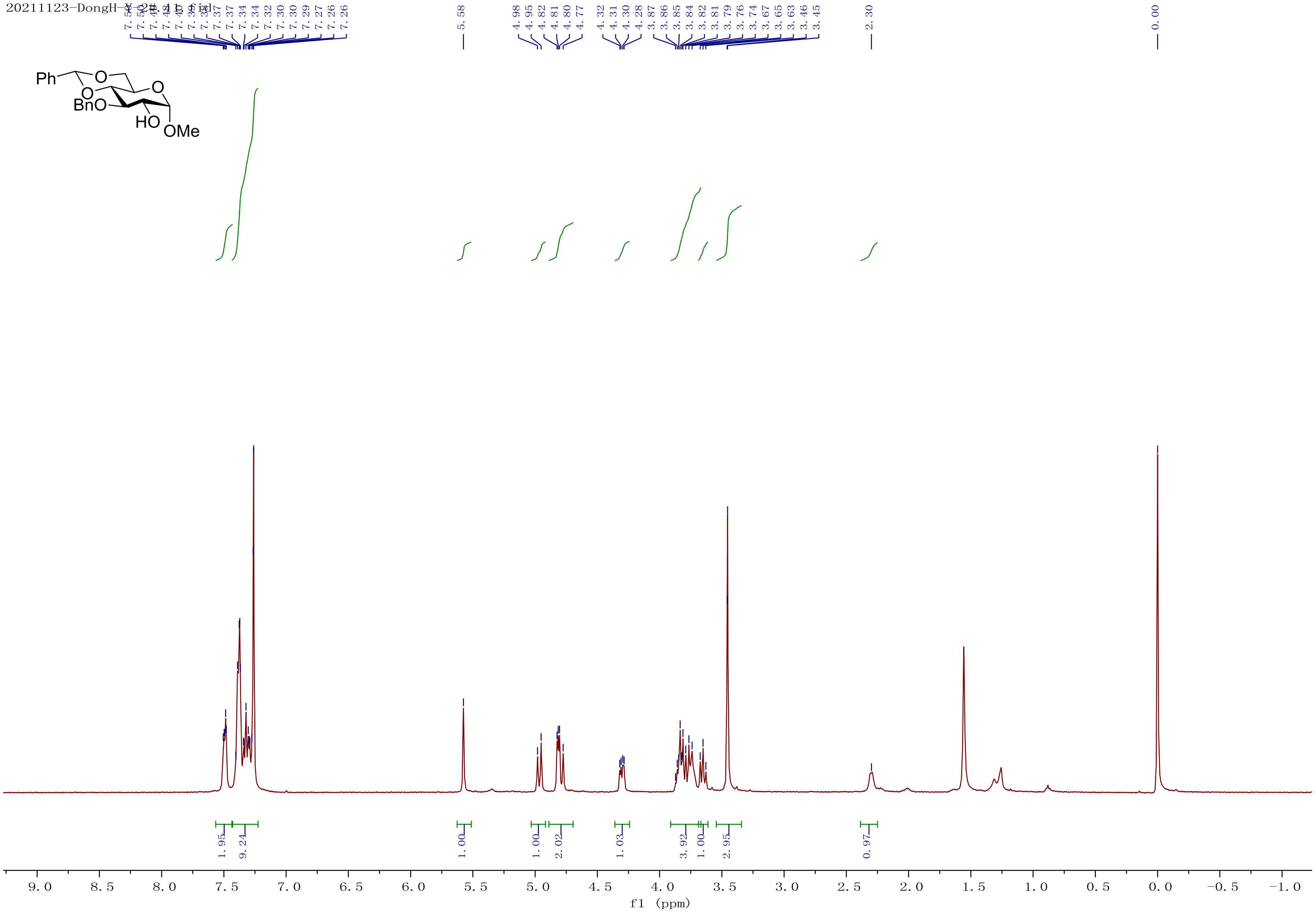

Methyl 2-*O*-*p*-methoxybenzyl-3-*O*-*tert*-butyl-dimethylsilyl-4,6-*O*-benzylidene-*α*-D-glucopyranoside 33

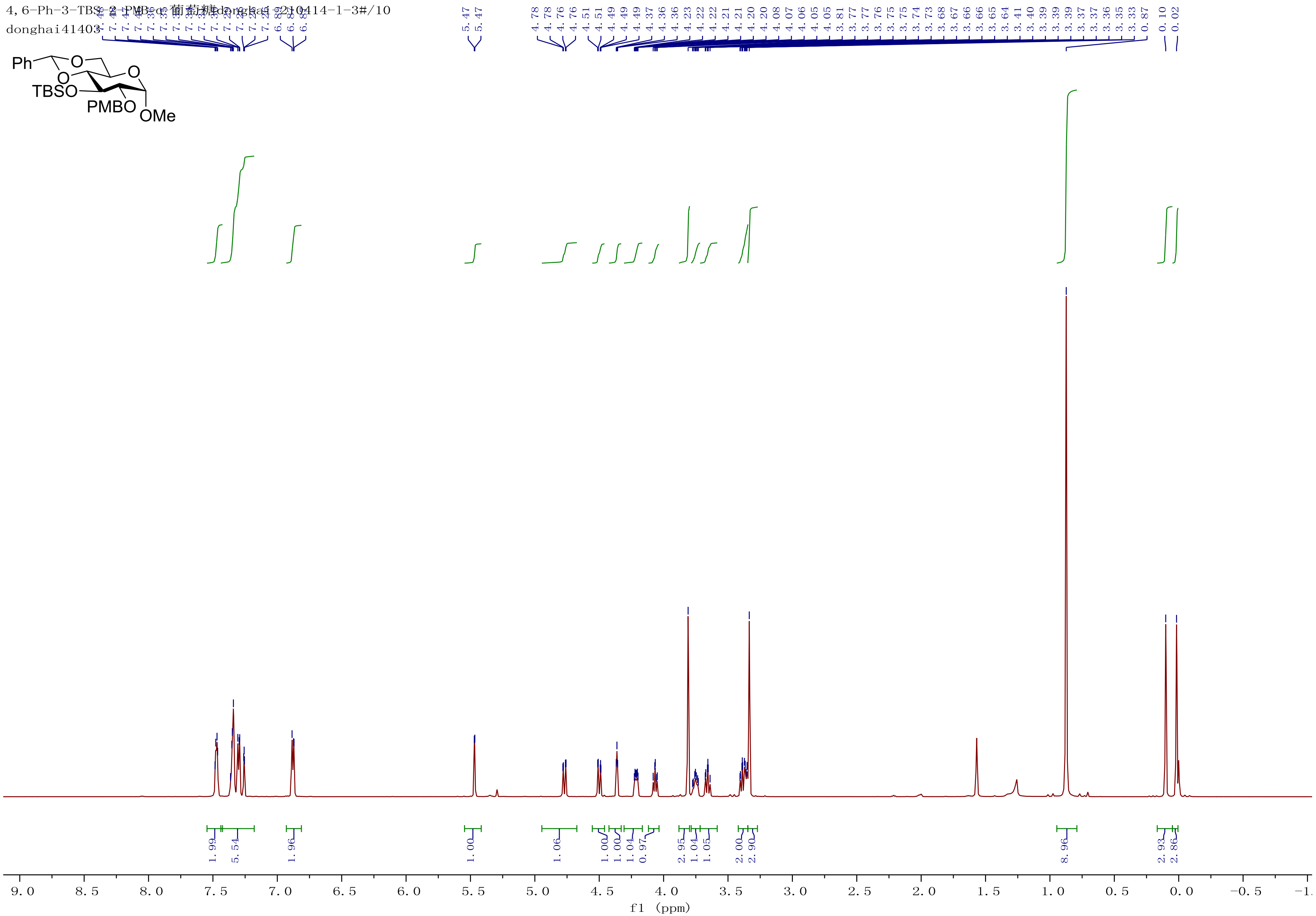

Methyl 2-*O*-*p*-methoxybenzyl-3-*O*-*tert*-butyl-dimethylsilyl-4,6-*O*-benzylidene-*α*-D-glucopyranoside 33

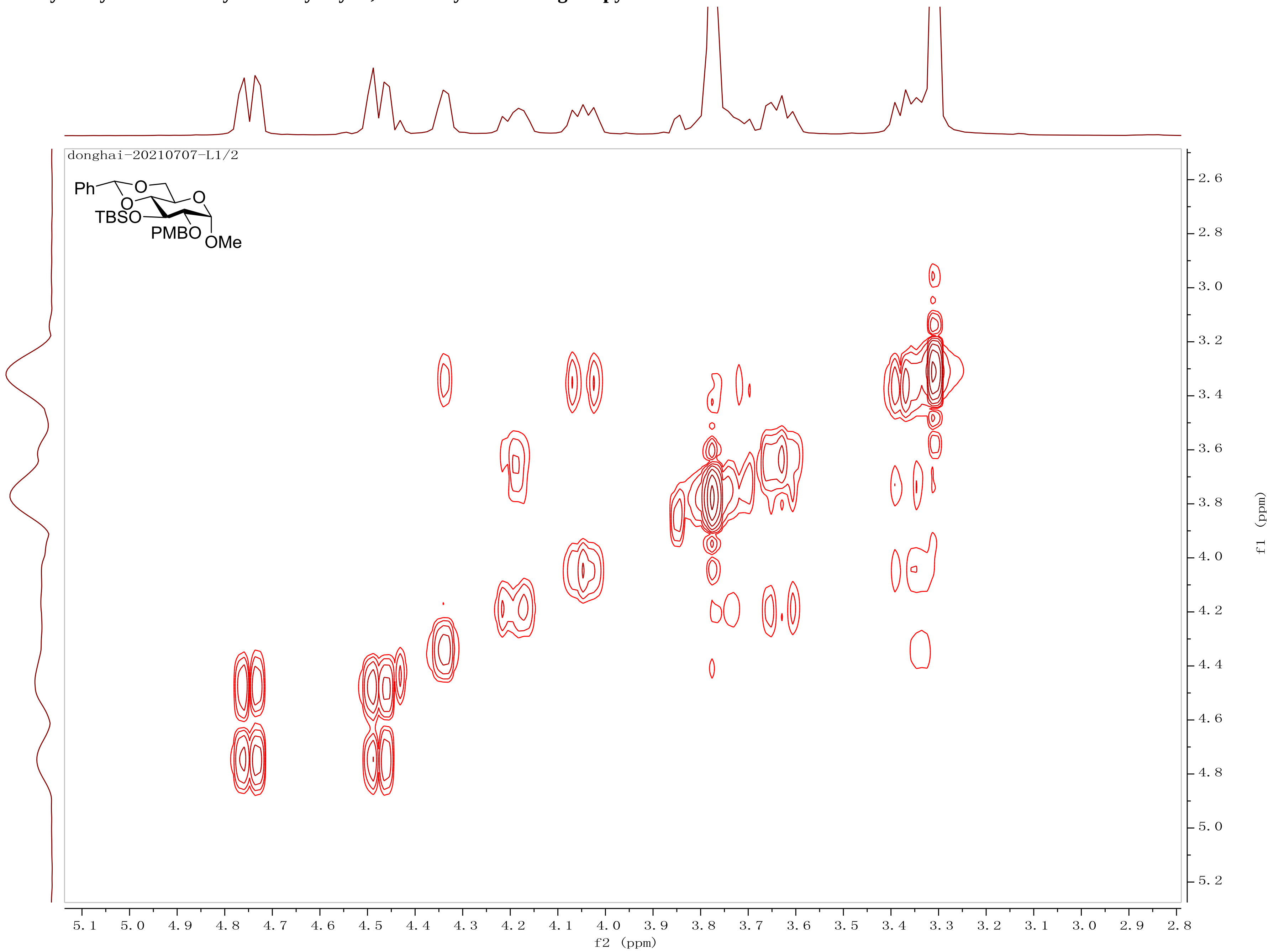

Methyl 2-*O*-*p*-methoxybenzyl-3-*O*-*tert*-butyl-dimethylsilyl-4,6-*O*-benzylidene-*α*-D-glucopyranoside 33

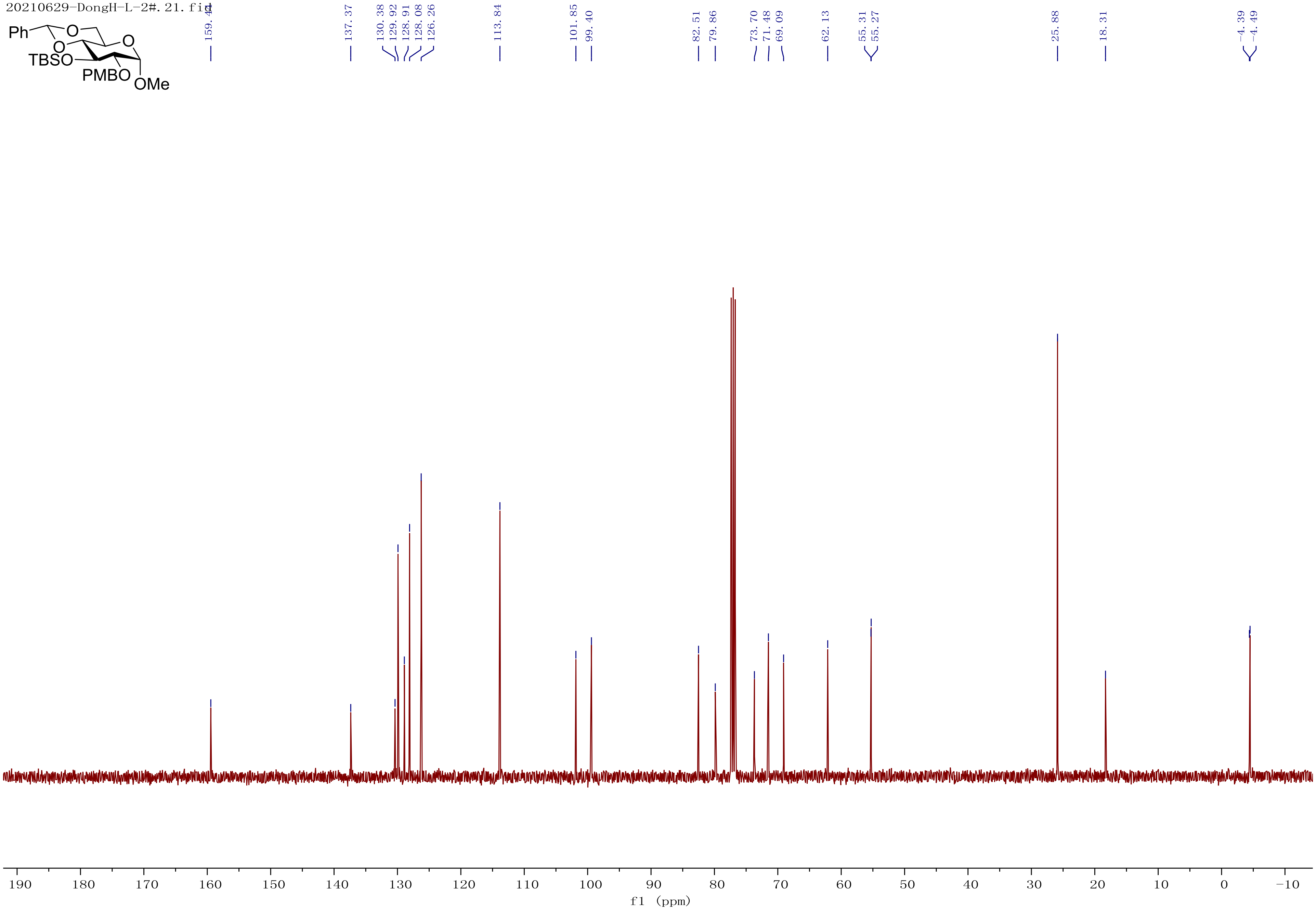

Methyl 3-*O*-*tert*-butyl-dimethylsilyl-4,6-*O*-benzylidene-*α*-D-glucopyranoside 34

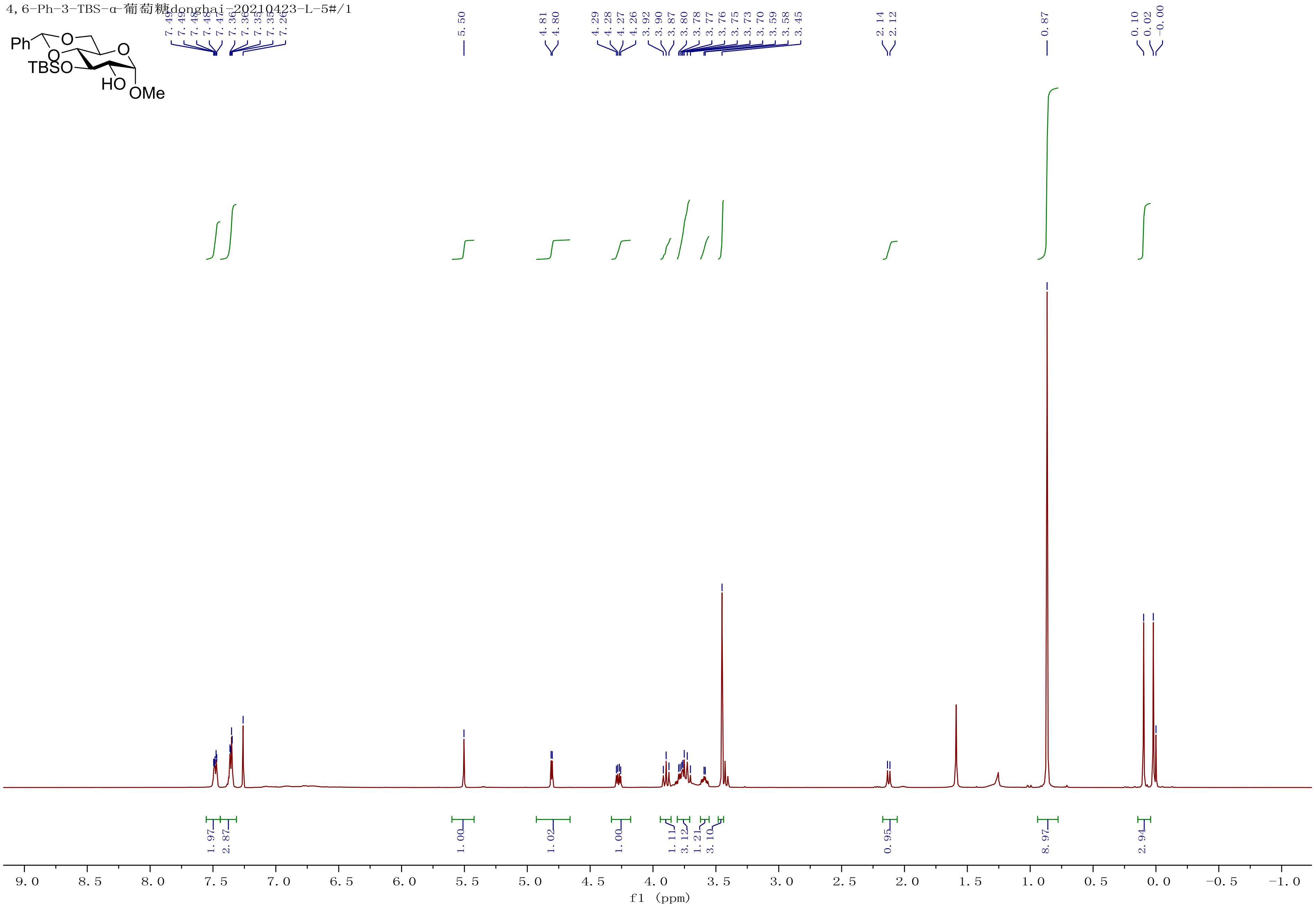

1,2:5,6-Di-*O*-isopropylidene-3-*O*-*p*-methoxybenzyl-*α*-D-glucofuranose 35

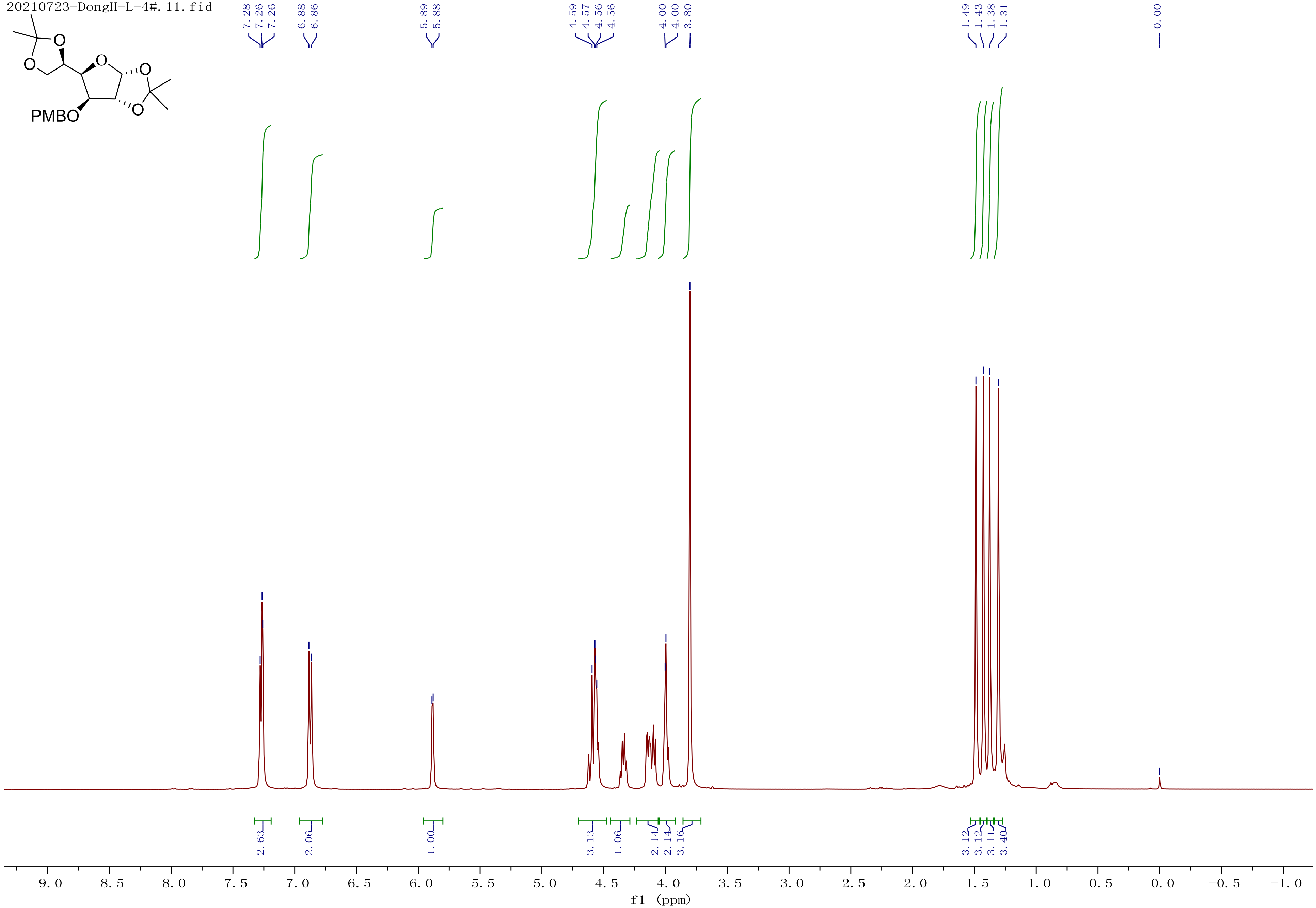

1,2:5,6-Di-*O*-isopropylidene-*α*-D-glucofuranose 36

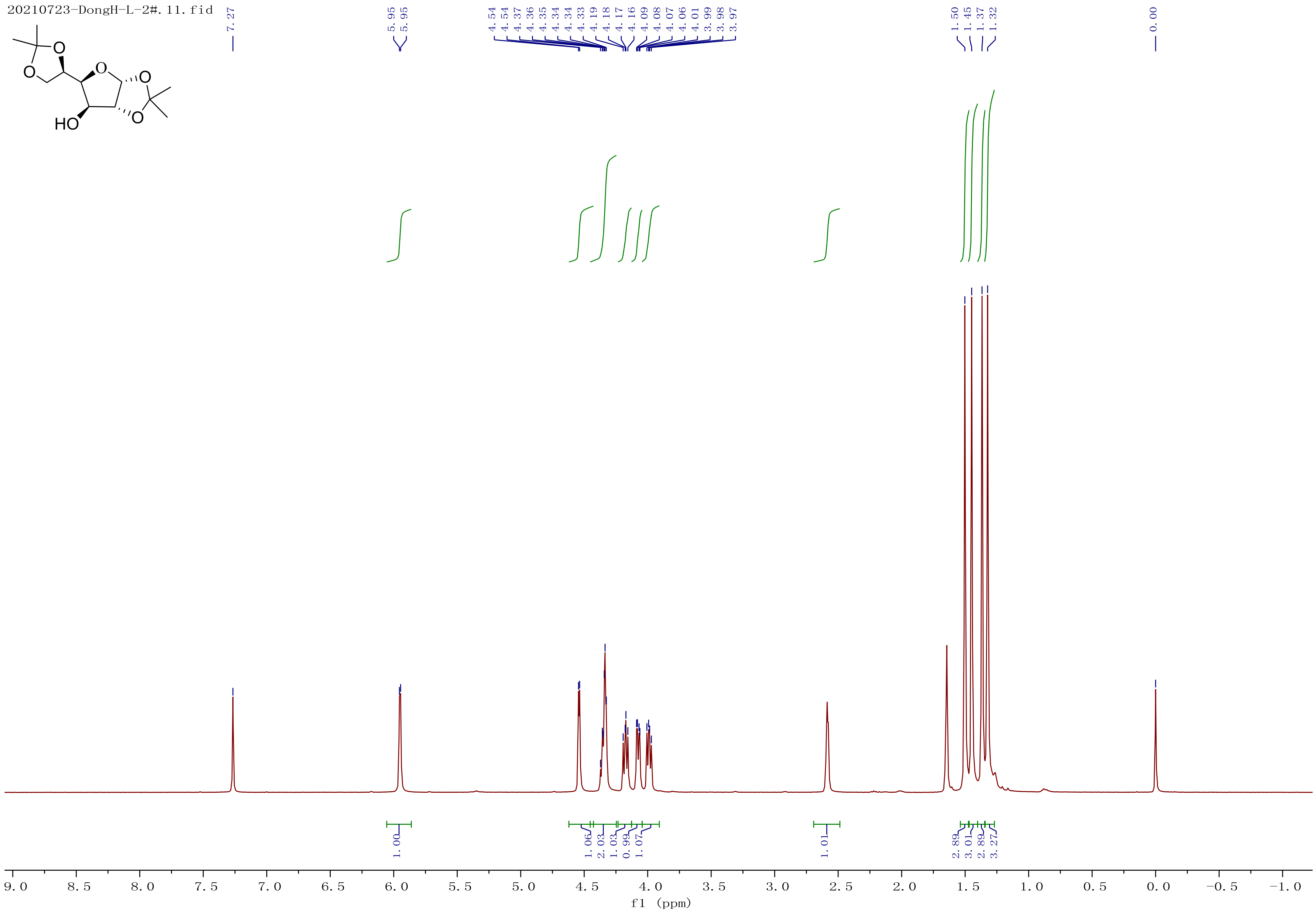

1,2:3,4-Di-*O*-isopropylidene-6-*O*-(*p*-methoxybenzyl)-*α*-D-galactopyranose 37

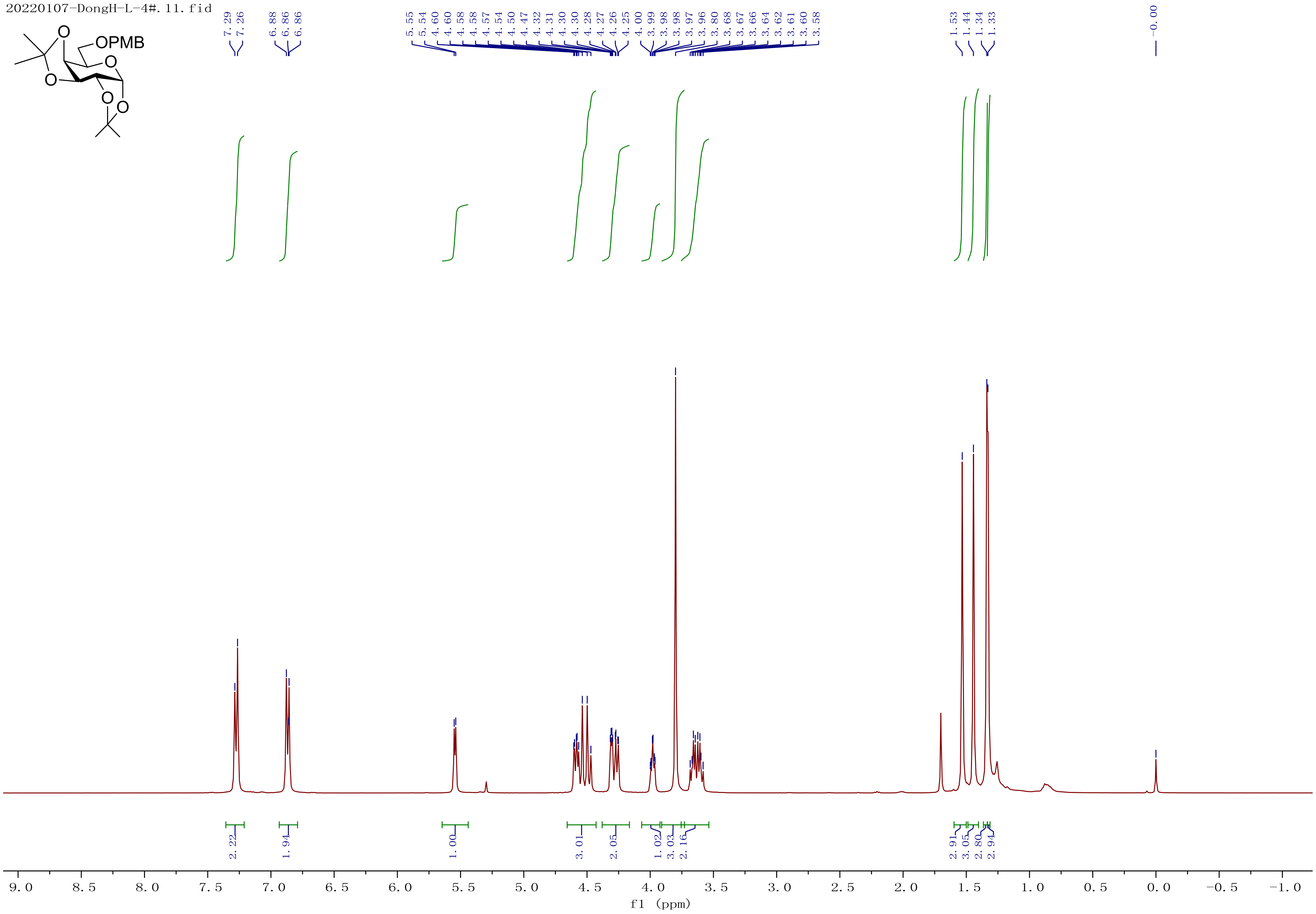

1,2:3,4-Di-*O*-isopropylidene-*α*-D-galactopyranose 38

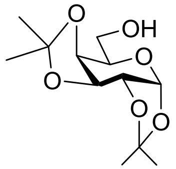

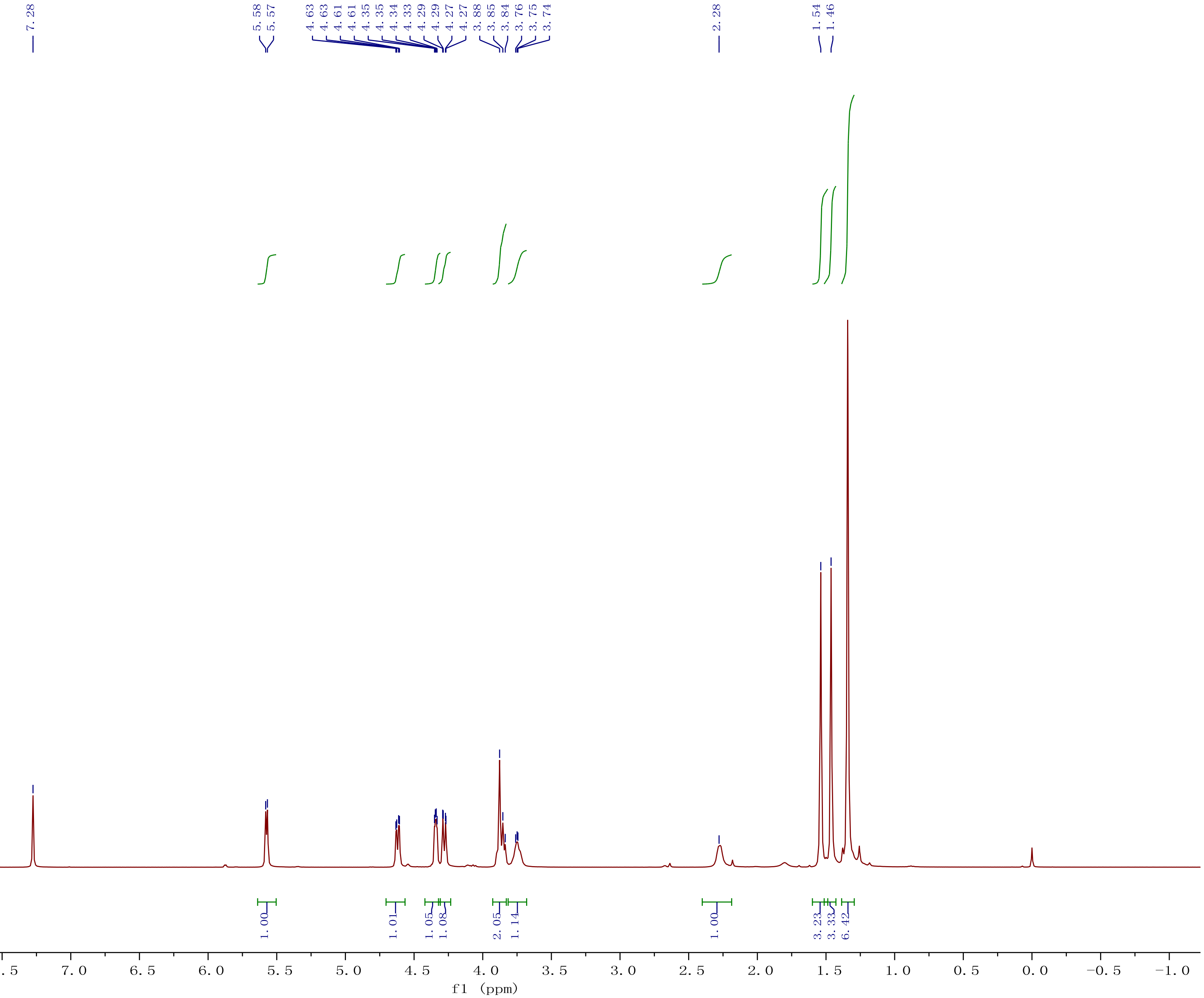

Methyl 2,3-di-*O*-benzyl-4,6-*O*-benzylidene-*α*-D-mannopyranoside 39

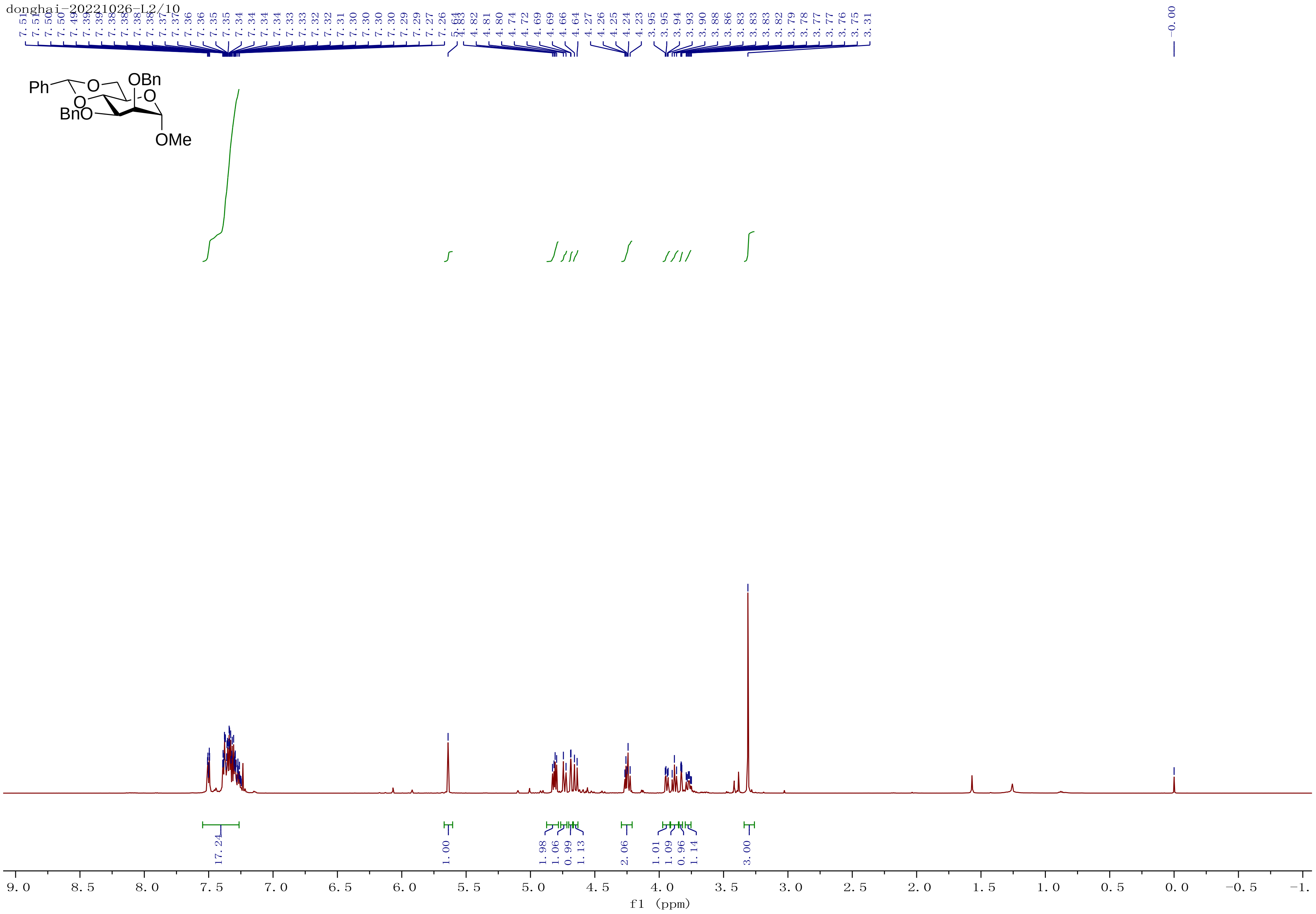

Methyl 2,3-di-*O*-benzyl-4,6-*O*-benzylidene-*β*-D-glucopyranoside 40

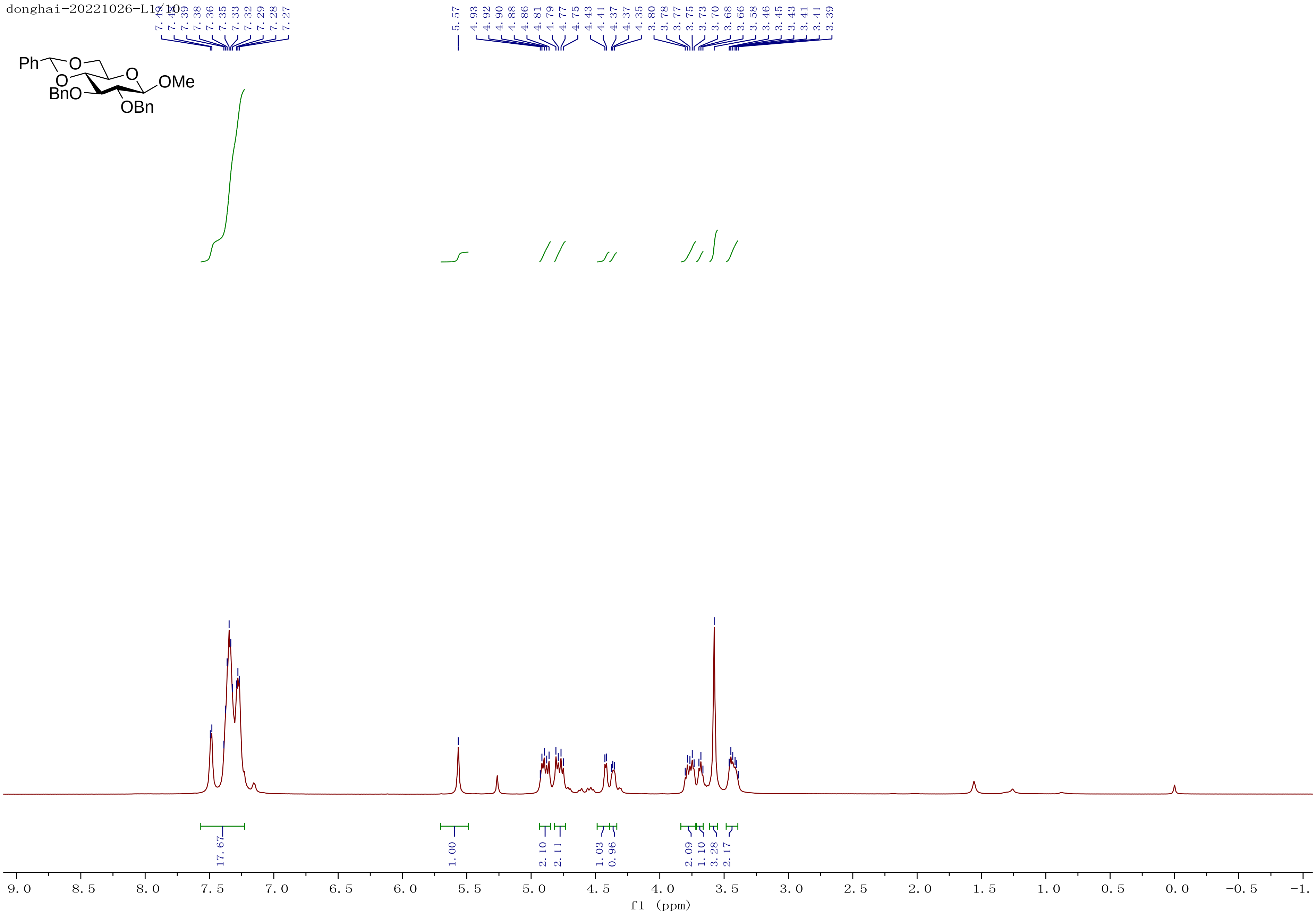

Methyl 2,3-di-*O*-benzyl-4,6-di-*O*-acetyl-*α*-D-galactopyranoside 41

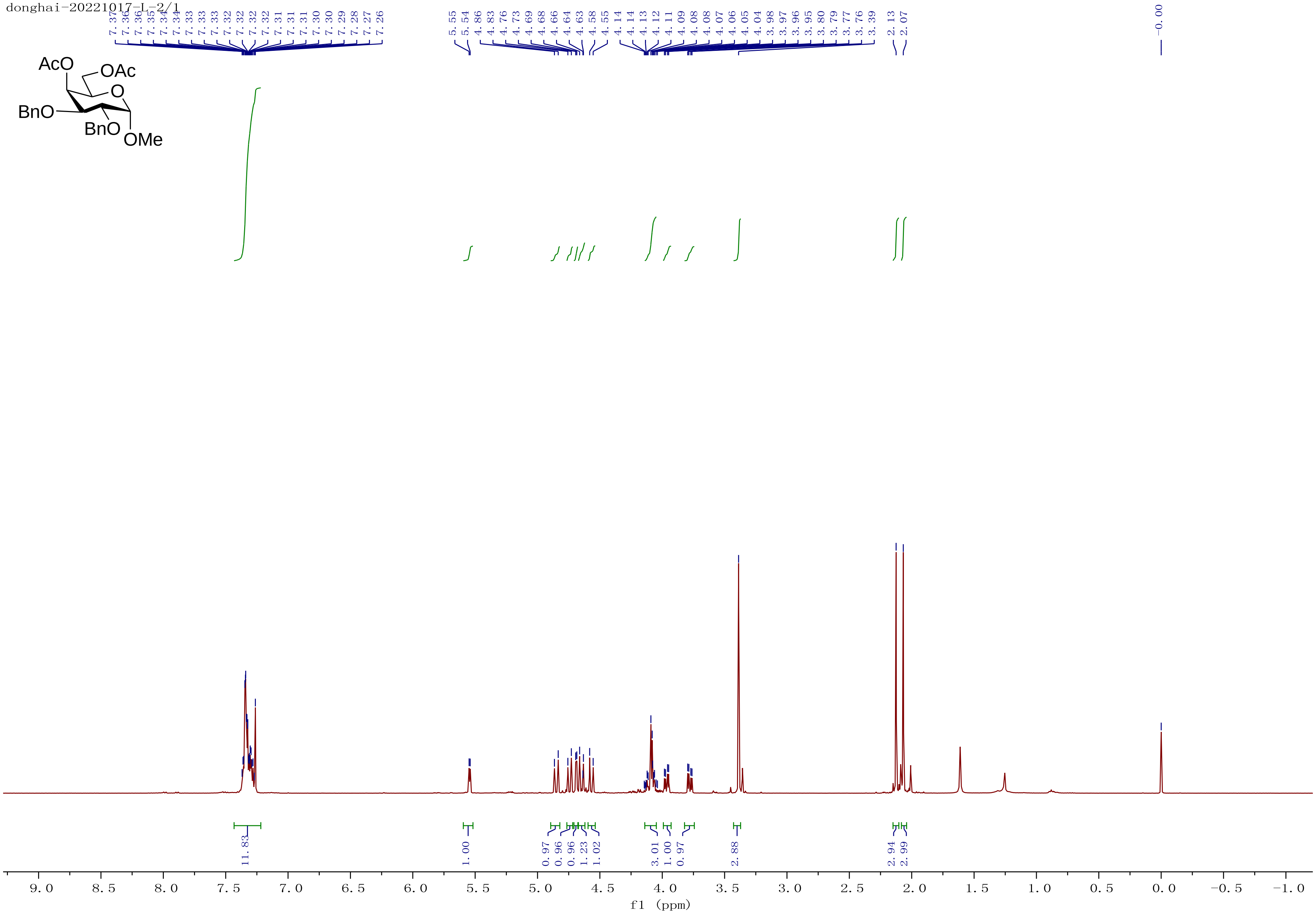

Methyl 2,3-di-*O*-benzyl-4,6-di-*O*-acetyl-*α*-D-mannopyranoside 42

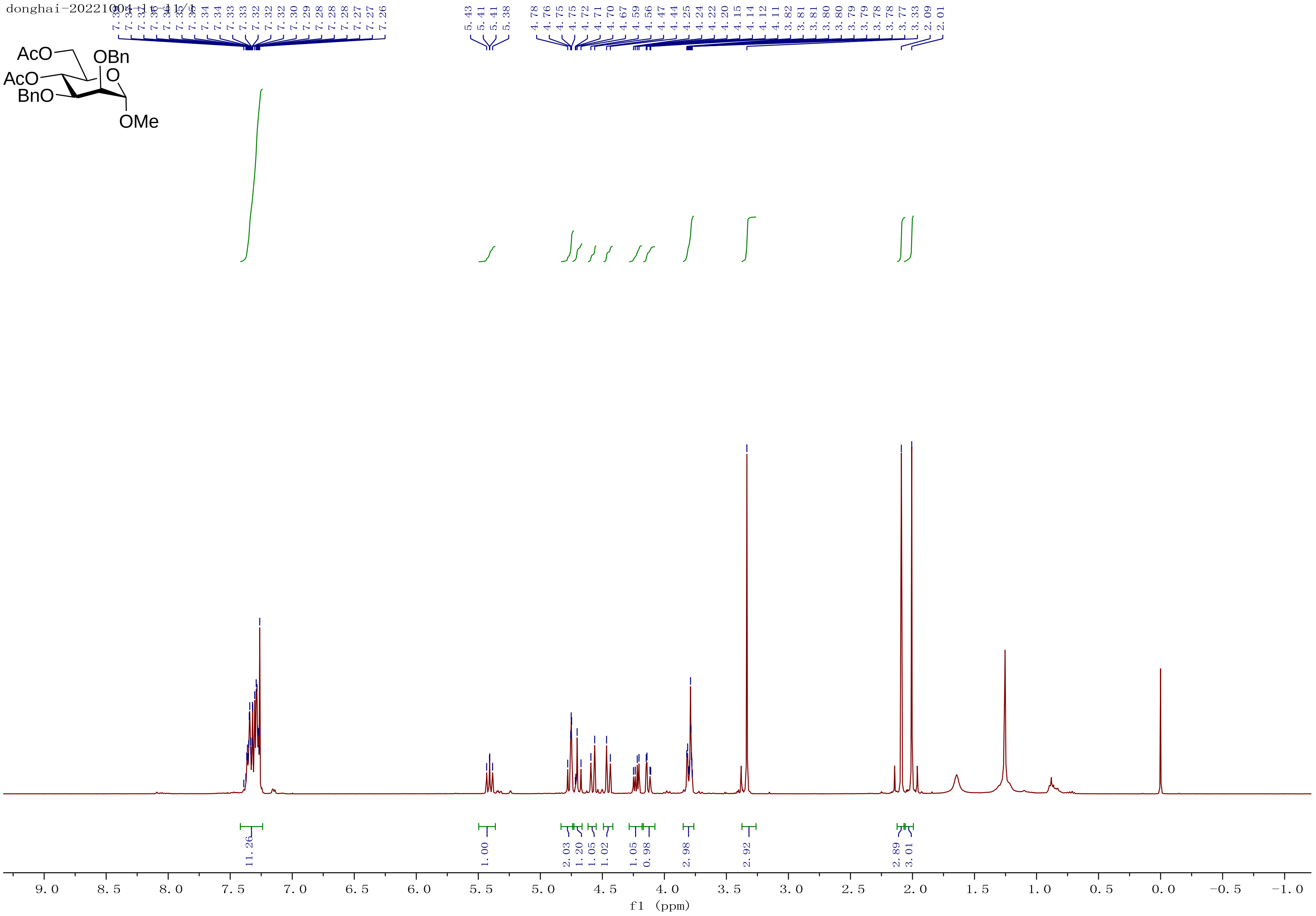

Methyl 2,3-di-*O*-benzyl-4,6-di-*O*-acetyl-*α*-D-mannopyranoside 42

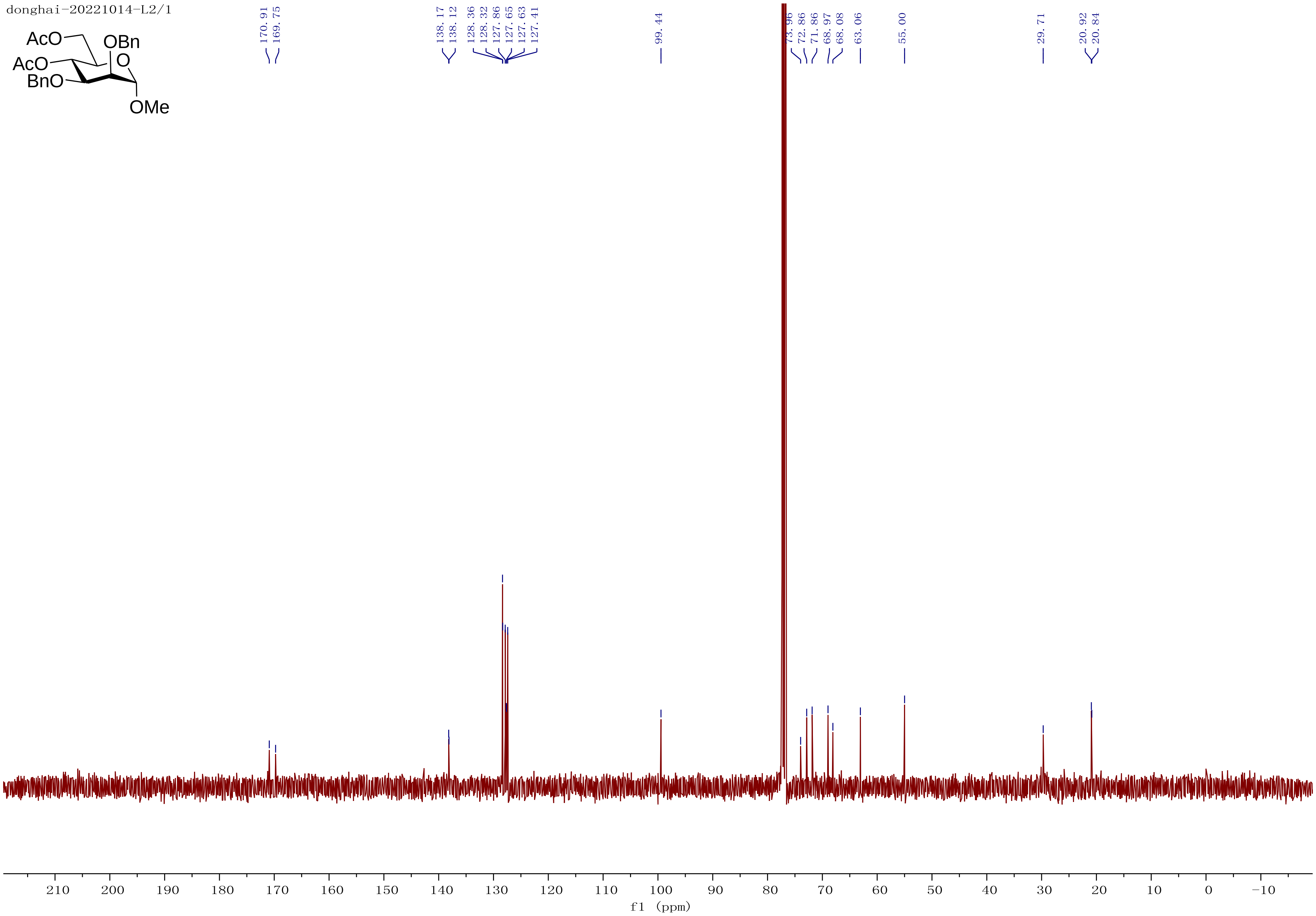

Methyl 2,3-di-*O*-benzyl-4,6-di-*O*-acetyl-*β*-D-glucopyranoside 43

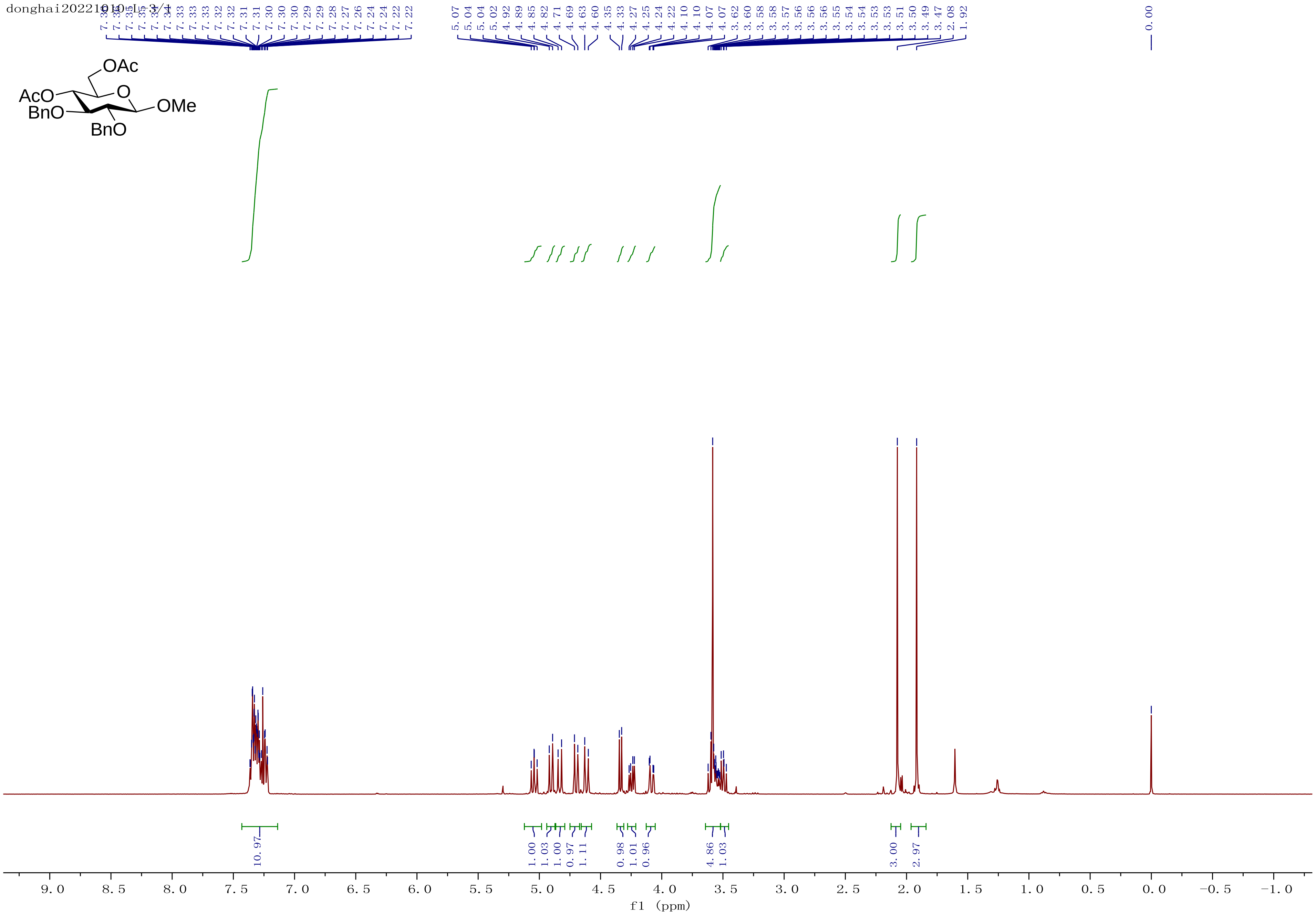

Methyl 2,3-di-*O*-benzyl-4,6-di-*O*-acetyl-*β*-D-glucopyranoside 43

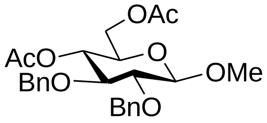

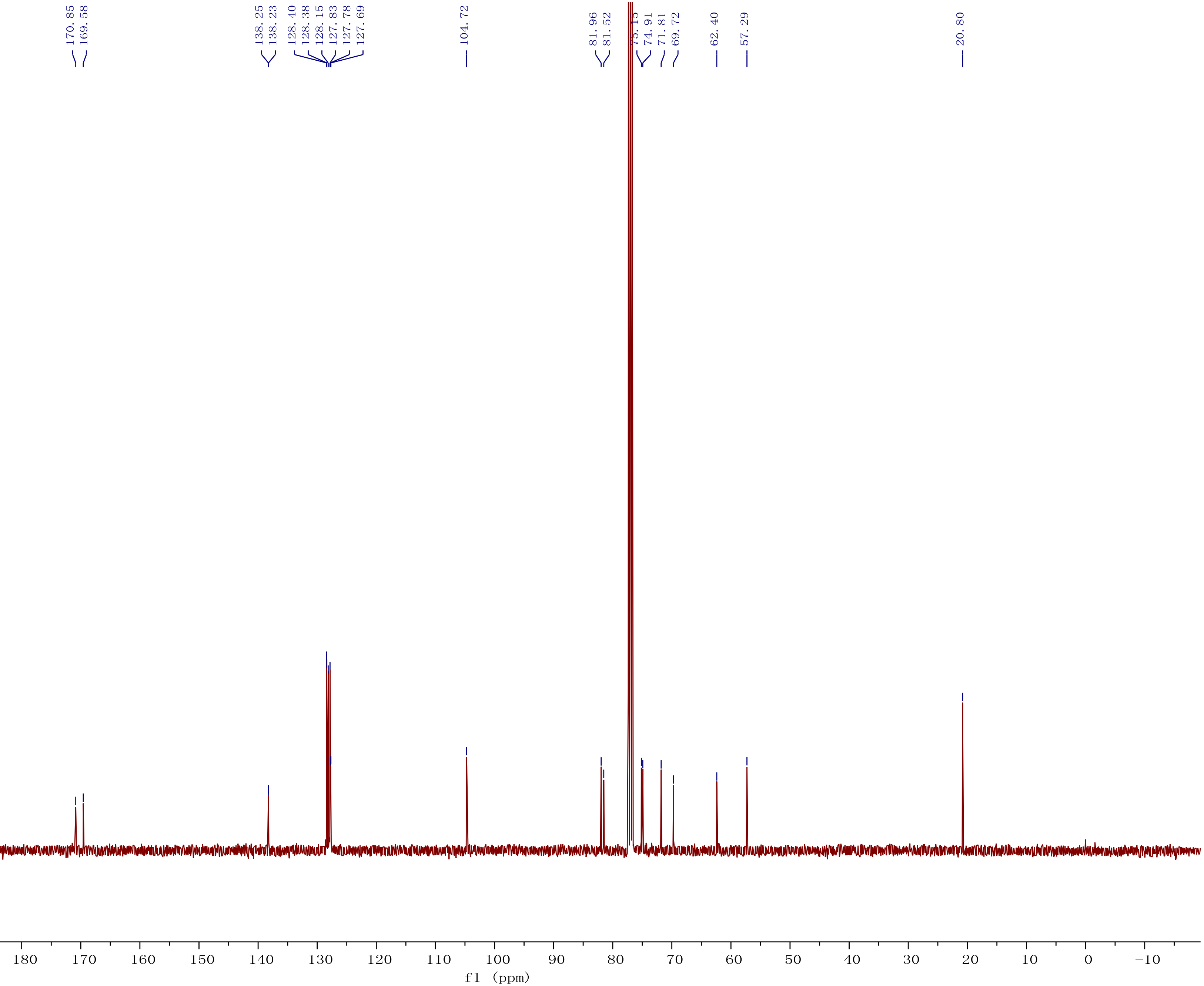

Methyl 2,3-di-*O*-benzyl-6-acetyl-*α*-D-glucopyranoside 44

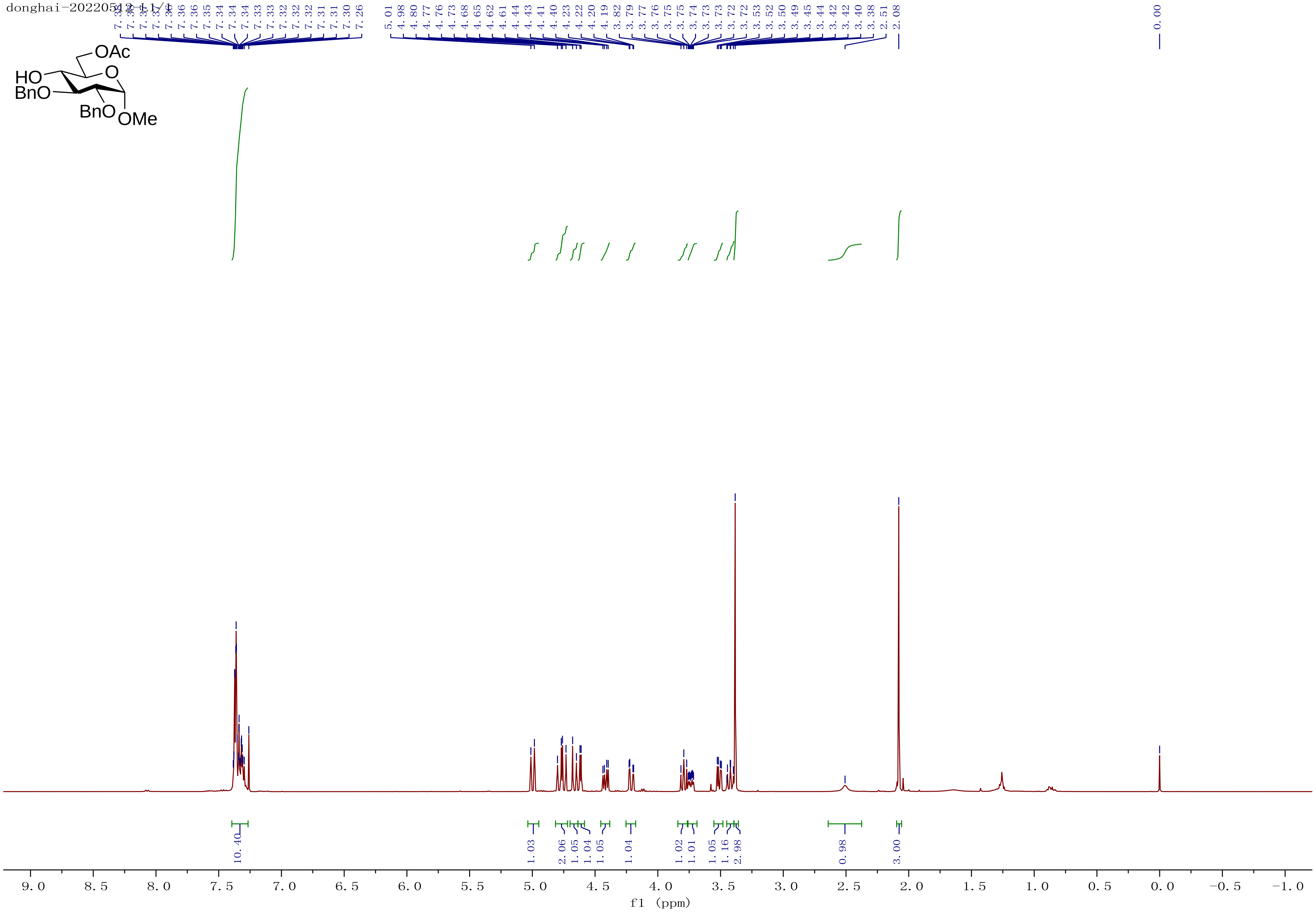

Methyl 2,3-di-*O*-benzyl-6-acetyl-*α*-D-galactopyranoside 45

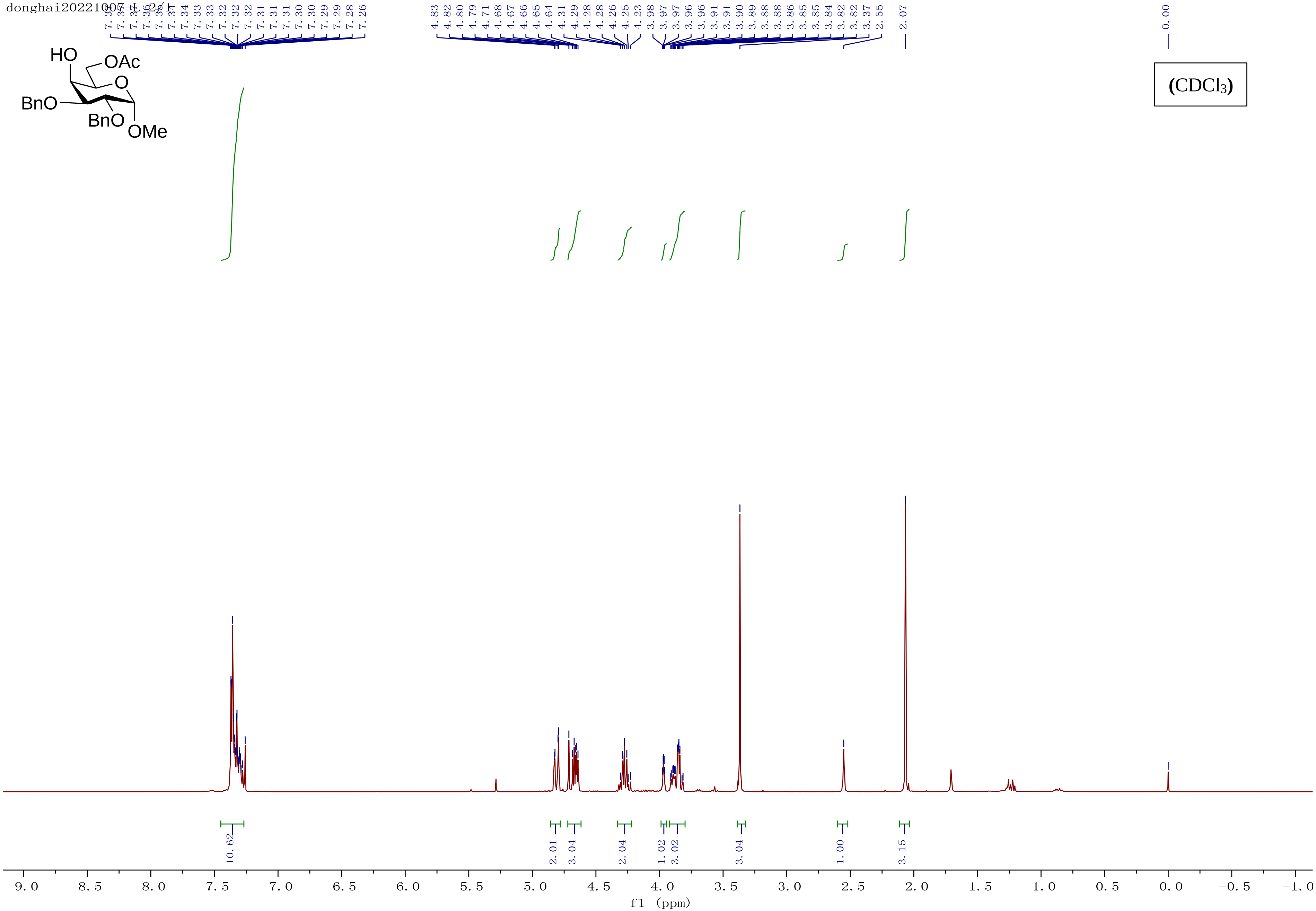

Methyl 2,3-di-*O*-benzyl-6-acetyl-*α*-D-galactopyranoside 45

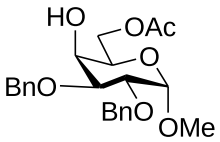

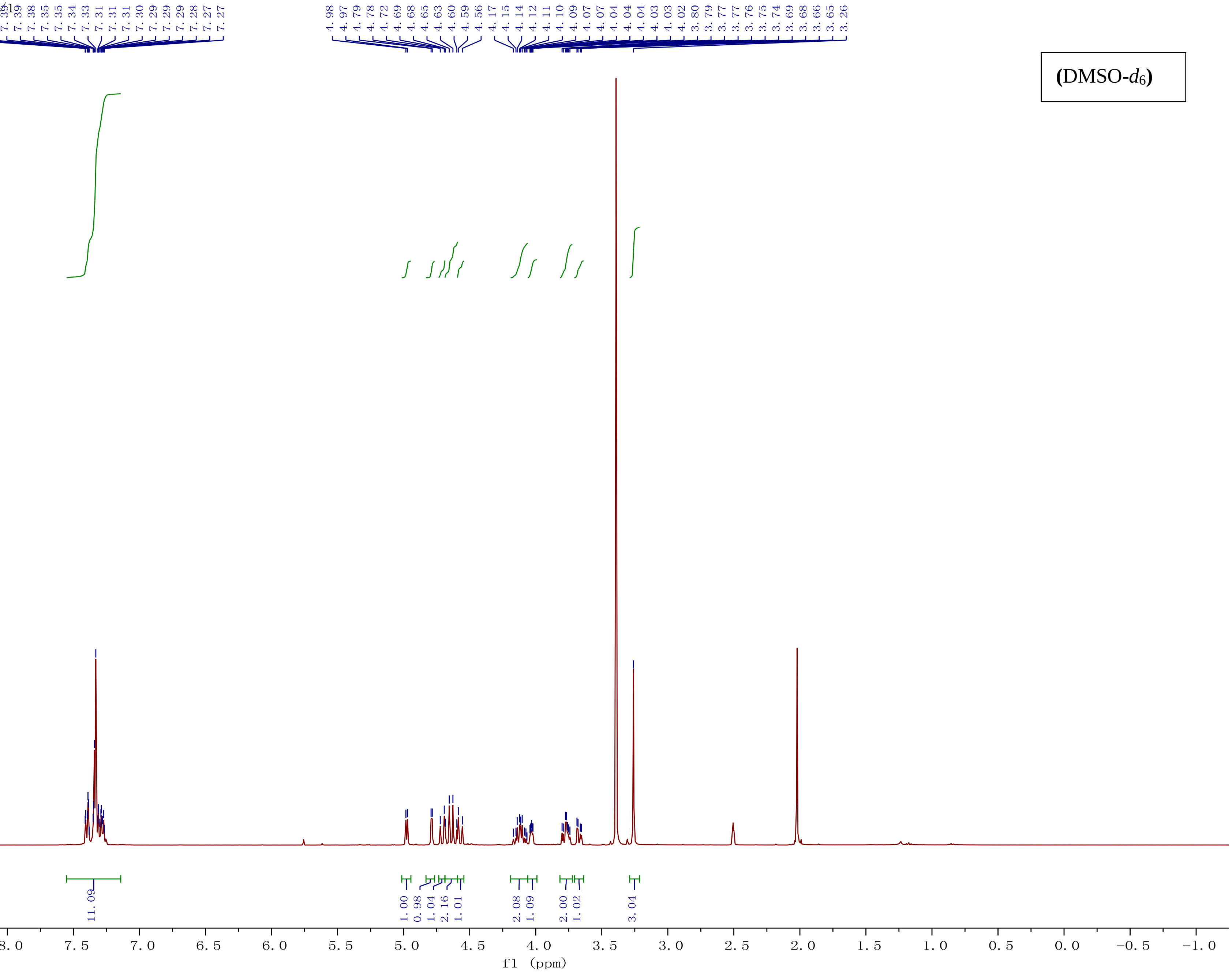

Methyl 2,3-di-*O*-benzyl-6-acetyl-*α*-D-mannopyranoside 46

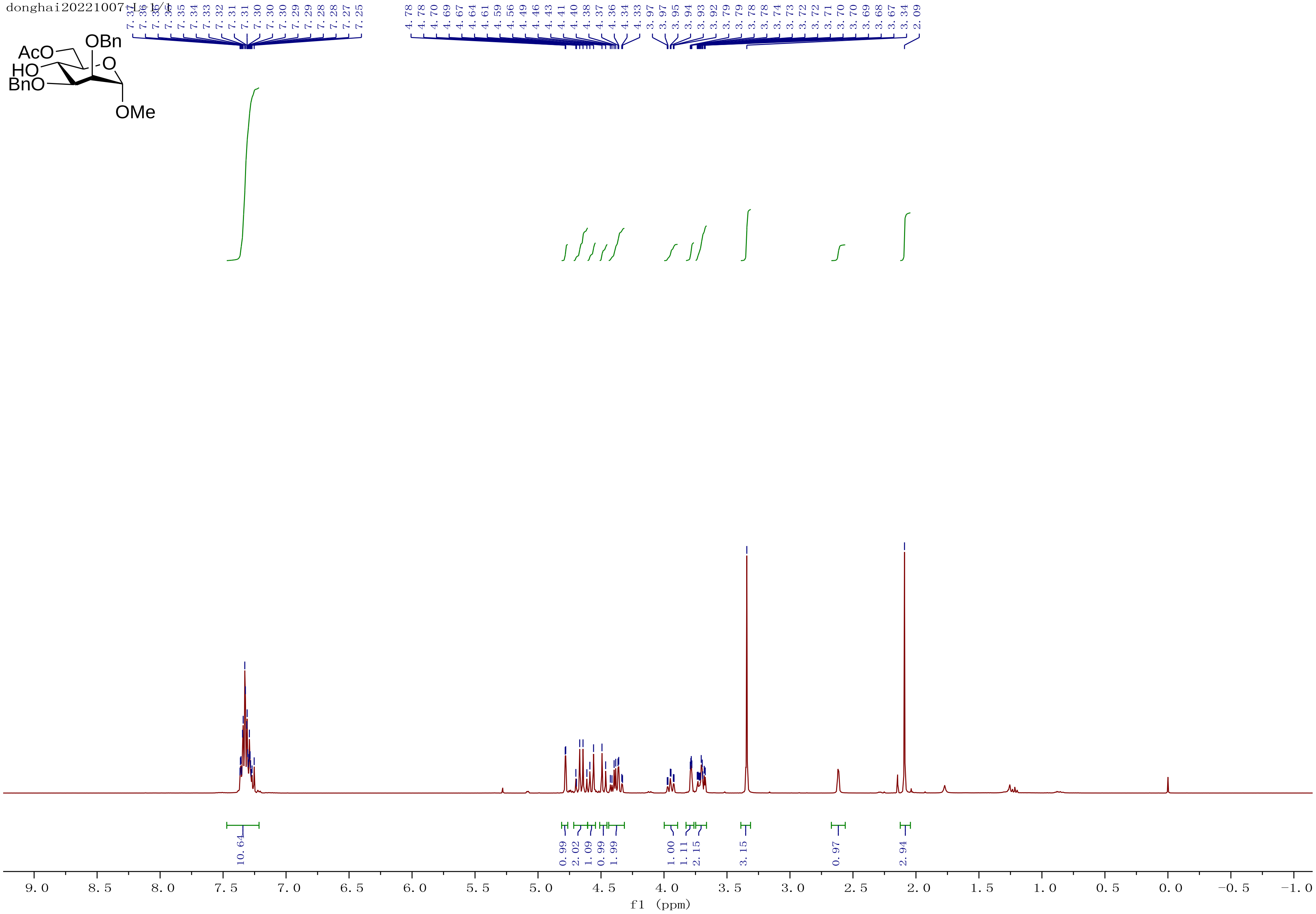

Methyl 2,3-di-*O*-benzyl-4,6-di-*O*-acetyl-*β*-D-glucopyranoside 47

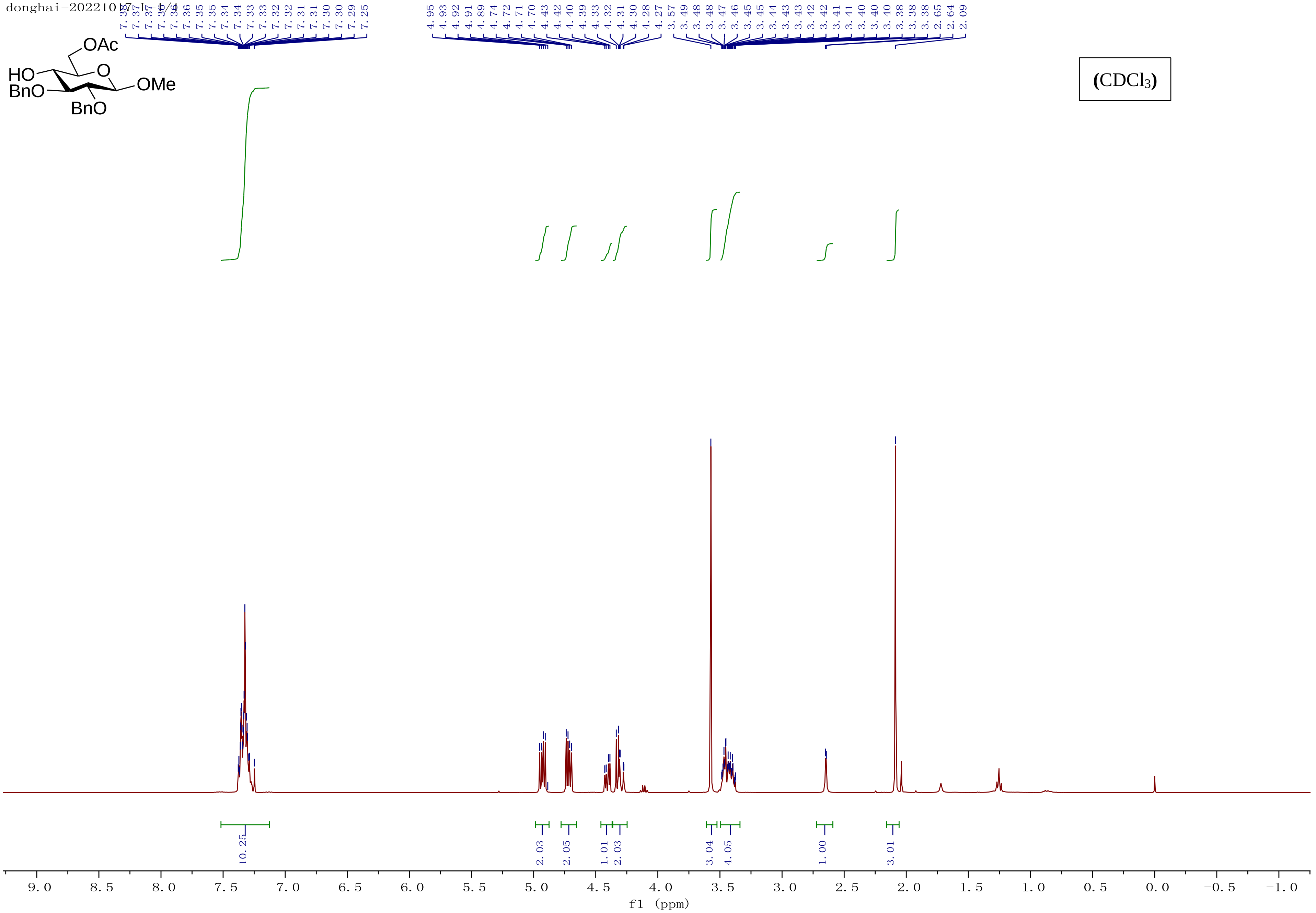

Methyl 2,3-di-*O*-benzyl-4,6-di-*O*-acetyl-*β*-D-glucopyranoside 47

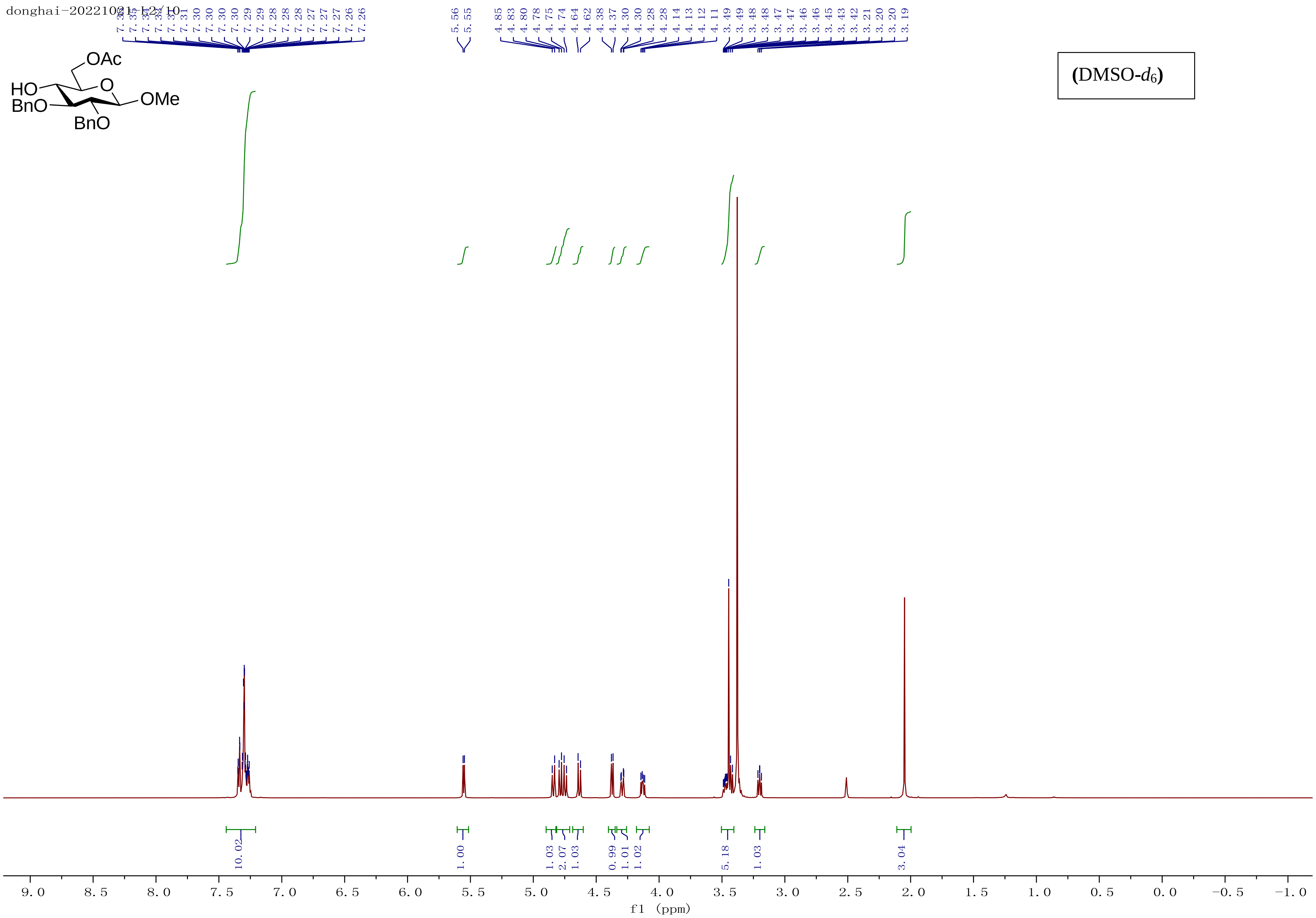

Supplement: Supplementary file 1 [file molecules-27-08258-s001.zip › molecules-2025547-supplementary.pdf]
